# Chromosome-scale assemblies of three *Ormosia* species: repetitive sequences distribution and structural rearrangement

**DOI:** 10.1093/gigascience/giaf047

**Published:** 2025-05-16

**Authors:** Zheng-Feng Wang, En-Ping Yu, Lin Fu, Hua-Ge Deng, Wei-Guang Zhu, Feng-Xia Xu, Hong-Lin Cao

**Affiliations:** Guangdong Provincial Key Laboratory of Applied Botany, South China Botanical Garden, Chinese Academy of Sciences, Guangzhou 510650, China; Key Laboratory of Vegetation Restoration and Management of Degraded Ecosystems, South China Botanical Garden, Chinese Academy of Sciences, Guangzhou 510650, China; Key Laboratory of National Forestry and Grassland Administration on Plant Conservation and Utilization in Southern China, South China Botanical Garden, Chinese Academy of Sciences, Guangzhou 510650, China; South China National Botanical Garden, Guangzhou 510650, China; Guangdong Provincial Key Laboratory of Applied Botany, South China Botanical Garden, Chinese Academy of Sciences, Guangzhou 510650, China; Key Laboratory of Vegetation Restoration and Management of Degraded Ecosystems, South China Botanical Garden, Chinese Academy of Sciences, Guangzhou 510650, China; Key Laboratory of National Forestry and Grassland Administration on Plant Conservation and Utilization in Southern China, South China Botanical Garden, Chinese Academy of Sciences, Guangzhou 510650, China; South China National Botanical Garden, Guangzhou 510650, China; University of Chinese Academy of Sciences, Beijing 100049, China; Guangdong Provincial Key Laboratory of Applied Botany, South China Botanical Garden, Chinese Academy of Sciences, Guangzhou 510650, China; Key Laboratory of National Forestry and Grassland Administration on Plant Conservation and Utilization in Southern China, South China Botanical Garden, Chinese Academy of Sciences, Guangzhou 510650, China; South China National Botanical Garden, Guangzhou 510650, China; Key Laboratory of Plant Resources Conservation and Sustainable Utilization, South China Botanical Garden, Chinese Academy of Sciences, Guangzhou 510650, China; Management Office of Guangdong Luofushan Provincial Nature Reserve, Huizhou 516133, China; Guangdong Provincial Key Laboratory of Applied Botany, South China Botanical Garden, Chinese Academy of Sciences, Guangzhou 510650, China; Key Laboratory of Vegetation Restoration and Management of Degraded Ecosystems, South China Botanical Garden, Chinese Academy of Sciences, Guangzhou 510650, China; Key Laboratory of National Forestry and Grassland Administration on Plant Conservation and Utilization in Southern China, South China Botanical Garden, Chinese Academy of Sciences, Guangzhou 510650, China; South China National Botanical Garden, Guangzhou 510650, China; Guangdong Provincial Key Laboratory of Applied Botany, South China Botanical Garden, Chinese Academy of Sciences, Guangzhou 510650, China; Key Laboratory of National Forestry and Grassland Administration on Plant Conservation and Utilization in Southern China, South China Botanical Garden, Chinese Academy of Sciences, Guangzhou 510650, China; South China National Botanical Garden, Guangzhou 510650, China; Key Laboratory of Plant Resources Conservation and Sustainable Utilization, South China Botanical Garden, Chinese Academy of Sciences, Guangzhou 510650, China; Guangdong Provincial Key Laboratory of Applied Botany, South China Botanical Garden, Chinese Academy of Sciences, Guangzhou 510650, China; Key Laboratory of Vegetation Restoration and Management of Degraded Ecosystems, South China Botanical Garden, Chinese Academy of Sciences, Guangzhou 510650, China; Key Laboratory of National Forestry and Grassland Administration on Plant Conservation and Utilization in Southern China, South China Botanical Garden, Chinese Academy of Sciences, Guangzhou 510650, China; South China National Botanical Garden, Guangzhou 510650, China

**Keywords:** comparative genomics, gene families, gene duplication, genetic diversity, outlier loci, population genetics, RNA-seq, repeat-mediated chromosome architectures, SNP calling, structure variation

## Abstract

**Background:**

The genus *Ormosia* belongs to the Fabaceae family; almost all *Ormosia* species are endemic to China, which is considered one of the centers of this genus. Thus, genomic studies on the genus are needed to better understand species evolution and ensure the conservation and utilization of these species. We performed a chromosome-scale assembly of *O. purpureiflora* and updated the chromosome-scale assemblies of *O. emarginata* and *O. semicastrata* for comparative genomics.

**Findings:**

The genome assembly sizes of the 3 species ranged from 1.42 to 1.58 Gb, with *O. purpureiflora* being the largest. Repetitive sequences accounted for 74.0–76.3% of the genomes, and the predicted gene counts ranged from 50,517 to 55,061. Benchmarking Universal Single-Copy Orthologs (BUSCO) analysis indicated 97.0–98.4% genome completeness, whereas the long terminal repeat (LTR) assembly index values ranged from 13.66 to 17.56, meeting the “reference genome” quality standard. Gene completeness, assessed using BUSCO and OMArk, ranged from 95.1% to 96.3% and from 97.1% to 98.1%, respectively.

Characterizing genome architectures further revealed that inversions were the main structural rearrangements in *Ormosia*. In numbers, density distributions of repetitive elements revealed the types of Helitron and terminal inverted repeat (TIR) elements and the types of *Gypsy* and unknown LTR retrotransposons (LTR-RTs) concentrated in different regions on the chromosomes, whereas *Copia* LTR-RTs were generally evenly distributed along the chromosomes in *Ormosia*.

Compared with the sister species *Lupinus albus, Ormosia* species had lower numbers and percentages of resistance (*R*) genes and transcription factor genes. Genes related to alkaloid, terpene, and flavonoid biosynthesis were found to be duplicated through tandem or proximal duplications. Notably, some genes associated with growth and defense were absent in *O. purpureiflora*.

By resequencing 153 genotypes (∼30 Gb of data per sample) from 6 *O. purpureiflora* (sub)populations, we identified 40,146 single nucleotide polymorphisms. Corresponding to its very small populations, *O. purpureiflora* exhibited low genetic diversity.

**Conclusions:**

The *Ormosia* genome assemblies provide valuable resources for studying the evolution, conservation, and potential utility of both *Ormosia* and Fabaceae species.

## Data description

### Context

The genus *Ormosia* Jackson, belonging to the Fabaceae family, comprises approximately 130–150 species [1, 2]. These species are trees and shrubs that thrive in warm climates. Fossil records suggest that *Ormosia* species were originally distributed in northern regions of the Northern Hemisphere and migrated southwards during the Paleogene or Neogene period due to climate cooling [3]. Today, their distributions span tropical America, Southeast Asia, and northern Australia [1–3], following a typical Asian–American tropical disjunction pattern [1]. Continental Asia is widely considered the center of origin for the genus.

**Figure 1:**
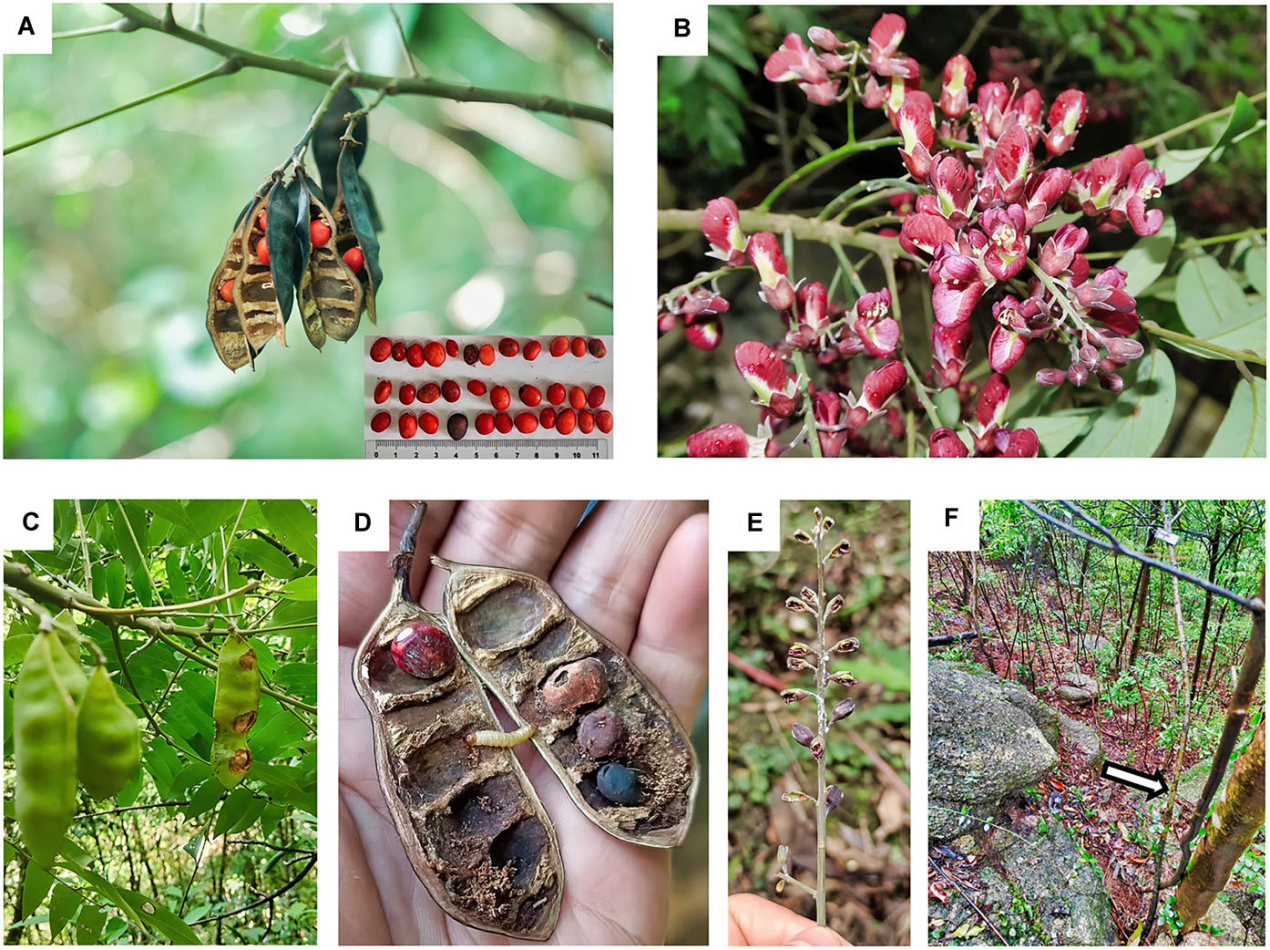
Photographs of *Ormosia purpureiflora*. (A) *O. purpureiflora* seeds. Seed sizes measured using a ruler are illustrated in the lower right panel. (B) *O. purpureiflora* flowers. (C) *O. purpureiflora* fruit in the distance showing a diseased state or insect invasion. (D) *O. purpureiflora* seeds showing invasion by worms/insects or a diseased state. (E) *O. purpureiflora* flowers in a diseased state or under insect invasion. (F) *O. purpureiflora* natural habitat; the arrow shows the sampled individual (a small shrub) used for genome assembly.

One of the most distinctive features of *Ormosia* species are their brightly colored seeds, including red, orange, bicolored red/orange, or black (Fig. 1A), These seeds are commonly used in ethnic jewelry and other decorative applications [1]. In addition, certain *Ormosia* species have high-value timber, and are cultivated as ornamental landscape trees [4, 5]. Extracts from their seeds, roots, stems, bark, and leaves have medicinal applications [6, 7] due to containing bioactive compounds such as alkaloids, flavonoids, isoflavones, terpenes, and lignans [6–8]. Metabolomic and transcriptomic analyses have further revealed that transcription factors play a key role in the regulation of flavonoid and terpenoid biosynthesis in *Ormosia* species [9, 10].

In China, approximately 37 species of *Ormosia* exist, and 34 of them are endemic [2]. *O. purpureiflora* is one such endemic species. Unlike most *Ormosia* species, which typically have white or yellow flowers, *O. purpureiflora* (NCBI:txid2866711) is characterized by its purple flowers (Fig. 1B), as reflected in its species name (*purpureiflora*). This species has been identified at only two locations in southeast China: the Guangdong Longmen Nankunshan (NKS) Provincial Natural Reserve and the Guangdong Luofushan (LFS) Provincial Natural Reserve. Field investigations have revealed approximately 2,000 individuals across these two sites [11]. A detailed survey of five plots (each 20 m × 20 m, 4 in LFS and 1 in NKS) recorded a total of 1,468 individuals. *O. purpureiflora* is a small shrub, with a diameter at breast height not exceeding 5 cm (average, 1.74 cm) and a height of no more than 4.5 m (average, 1.02 m). The species produces bisexual flowers in panicles or racemes and it is primarily insect pollinated, with bees as the main pollinators. Although it exhibits typical outcrossing, self-pollination is also possible, as confirmed by bagging experiments [11]. Root-derived clonal reproduction has also been observed in the field [11]. The species is subject to severe pest and disease attacks, affecting both flowers and fruit (Fig. 1C–E), resulting in low seed yields in its natural habitat.

Advancements of high-throughput sequencing technologies have enabled the assembly of full genome information in diversified species, facilitating their conservation, restoration, management, and utilization. In this study, we generated a high-quality genome of *O. purpureiflora* by using a combination of long- and short-read whole genome sequencing (WGS), high-throughput chromosome conformation capture (Hi-C) sequencing, and RNA sequencing (RNA-Seq) of different tissues for annotation. In addition, we examined genetic diversity and conducted population genetics analyses for *O. purpureiflora* by resequencing 153 samples collected from 2 sites.

Genomic studies in *Ormosia* have primarily focused on its chloroplasts, with approximately 15 species, including *O. purpureiflora*, having been studied [12, 13]. To date, only 1 mitochondrial genome has been reported, which is from *O. boluoensis* [14]. Moreover, draft nuclear genomes have been reported for only 2 species, *O. emarginata* (NCBI:txid53908) and *O. semicastrata* (NCBI:txid499992) [15]. According to phylogenetic studies in Fabaceae [16], *Ormosia* belongs to the Genistoid lineage, where it is most closely related to *Hovea* and *Poecilanthe*. However, no genomes have been reported for these two genera.

For comparative genomics, we updated the genome assemblies of previously published *O. emarginata* and *O. semicastrata* genomes [15] by using Hi-C data to generate chromosome-scale assemblies. Compared with *O. purpureiflora*, both *O. emarginata* and *O. semicastrata* are widespread species in southern and southeastern China, with *O. emarginata* extending into Vietnam. Unlike the small shrub *O. purpureiflora*, both *O. emarginata* and *O. semicastrata* are large trees. Phylogenetic analyses conducted by Torke et al. [1] placed *O. emarginata* and *O. semicastrata* in different clades, with *O. emarginata* belonging to the Old World *Ormosia* clade 1 and *O. semicastrata* in the Old World *Ormosia* clade 2. However, *O. purpureiflora* was not included in these phylogenies.

## Methods

### Chromosome number observation

The individual used for chromosome number observation in *O. purpureiflora* was regenerated from seeds collected at LFS. Its root tips were pretreated with 0.002 M 8-hydroxyquinoline for 6 h and then fixed in a 3 : 1 (v:v) solution of absolute ethanol and glacial acetic acid for 24 h at room temperature. After fixation, the root tips were transferred to 70% ethanol and stored at −4°C until chromosome counts were performed. For chromosome counting, the fixed root tips were hydrolyzed in a 1 : 1 (v:v) solution of 1 M absolute ethanol and hydrochloric acid at room temperature for 7 min, rinsed with water, and then stained with carbol fuchsin for 4 min. Meristems were then excised and squashed for microscope observation. Photographs were taken using an Olympus BX-43 microscope (Olympus Corporation, TN, USA) at 100× magnification with an Olympus DP26 camera (Olympus Corporation, TN, USA).

### Sample collection and sequencing

An *O. purpureiflora* individual (Fig. 1F) collected from LFS was used for genome assembly. Genomic DNA was isolated from its leaf tissues and multiple libraries were constructed, including long- and short-read WGS and Hi-C libraries. For gene annotation, RNA was extracted from the same individual used for genome assembly, specifically from its leaves, flowers, seeds, and fruit. RNA-seq libraries were then constructed for these tissues. Long-read WGS was performed using the Oxford Nanopore Technologies (ONT) PromethION sequencer (Oxford Nanopore Technologies plc, Oxford, UK). Both long-read and ultra-long-read (50 kb) sequencing libraries were generated on the ONT platform. Short-read WGS, Hi-C, and RNA-seq were conducted using an MGI DNBSEQ-T7 (MGI Tech Co., Ltd., Shenzhen, China) sequencer with a 150 bp paired-end sequencing strategy (insert size, 300 bp). Given the relatively high error rate of ONT reads, the error profile of the ONT data was estimated using SeqFaiLR (Tools To Analyse Long Reads Sequencing Error Profile) [17].

**Figure 2:**
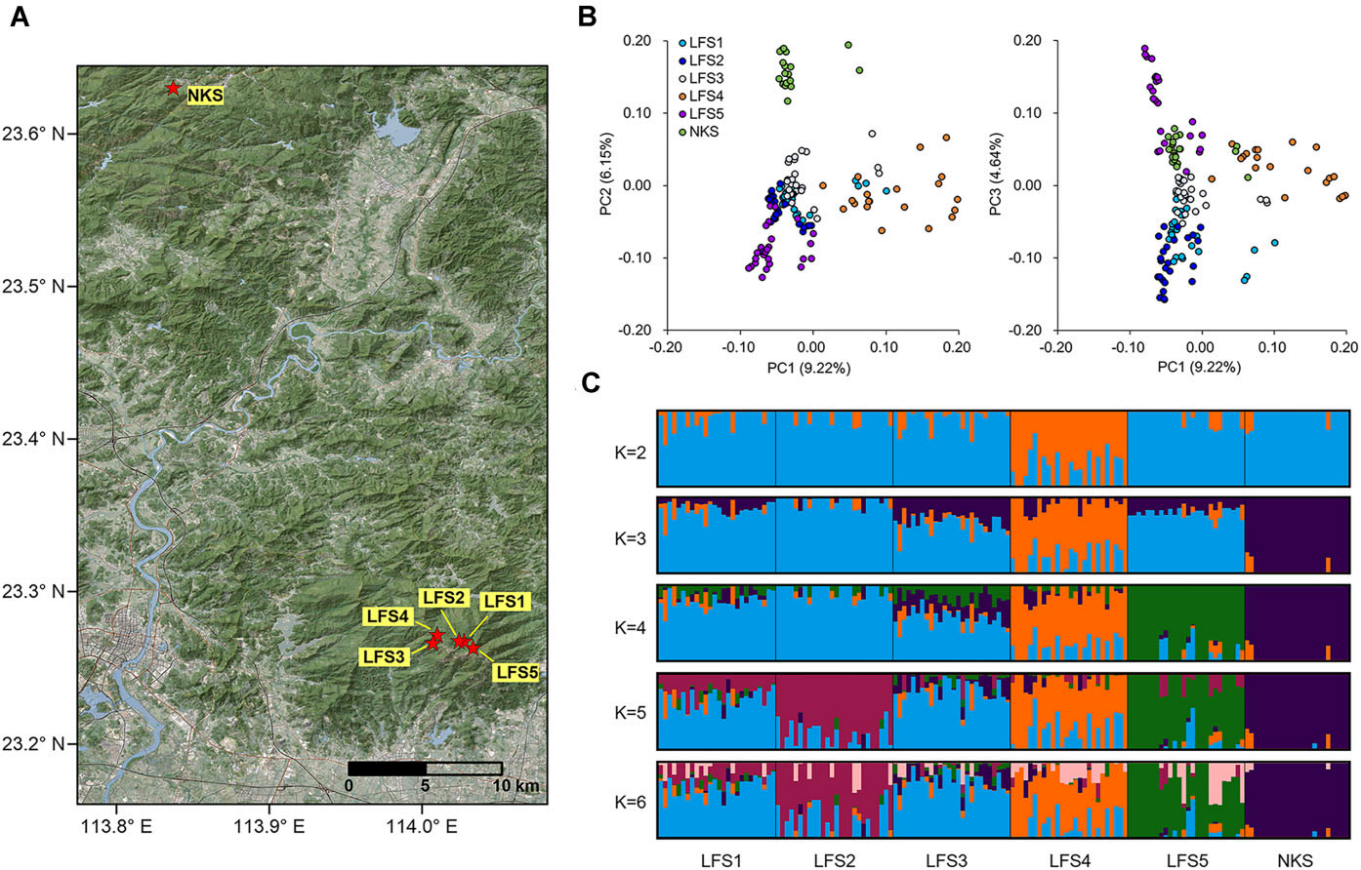
Sampled (sub)populations and population genetics of *Ormosia purpureiflora*. (A) Map showing (sub)populations sampled for *Ormosia purpureiflora*. (B) Principal component analysis (PCA) results showing the first 3 PCs (PC1 versus PC2; PC1 versus PC3) for *O. purpureiflora* individuals sampled from different (sub)populations. (C) Admixture results representing data for *K* = 2–6 clusters.

For population genetic studies on *O. purpureiflora*, leaf samples were collected from 153 individuals representing 6 (sub)populations in LFS and NKS (Table 1, Fig. 2A). These individuals were randomly selected to ensure that their distribution covered the entire range of the two sites based on a thorough field investigation [11]. The geographical positions of the sampled individuals were recorded using a handheld GPS. Leaves from each individual were immediately placed into sealed plastic bags containing silica gel for preservation. Whole-genome resequencing was conducted using an MGI DNBSEQ-T7 sequencer with a PE-150 bp model, generating approximately 30 Gb of data per sample.

**Table 1:** Six sampled (sub)populations and their genetic diversities in *Ormosia purpureiflora*

(Sub)population	Sample size	*Ho*	*He*	*Fis*	*π*
LFS1	26	0.171	0.148	-0.122	0.148
LFS2	26	0.154	0.130	0.000	0.128
LFS3	26	0.165	0.146	-0.083	0.147
LFS4	26	0.196	0.154	-0.268	0.152
LFS5	26	0.146	0.122	0.058	0.121
NKS	23	0.153	0.122	0.011	0.121

Leaf and flower RNA-seq libraries were constructed and sequenced by Annoroad Gene Technology (AGT, Beijing, China), and the remaining libraries were constructed and sequenced by GrandOmics Biosciences (GB, Wuhan, China).

For *O. emarginata* and *O. semicastrata*, Hi-C libraries were constructed using leaf samples from the same individuals used in their primary genome assembies [15], and sequencing was performed by GrandOmics Biosciences.

Detailed sequencing information, including DNA/RNA preparation and library construction (except for ONT ultra-long WGS sequencing), has been reported in our previous studies [18, 19]. Specifically, ONT long-read WGS sequencing was performed using the protocol described by Wang et al. [18], whereas short-read WGS, Hi-C, and RNA-seq sequencing were conducted using protocols described by Wang et al. [19]. For *O. purpureiflora* ONT ultra-long WGS sequencing, genomic DNA was extracted, and approximately 8–10 µg of DNA fragments longer than 50 kb were selected using the SageHLS HMW library system (Sage Science, Inc., MA, USA). The size-selected DNA was repaired using the NEBNext FFPE DNA Repair Mix (catalog no. M6630, New England Biolabs, MA, USA) in accordance with the manufacturer's instructions. End-repair and dA-tailing were then performed using the NEBNext Ultra II End-Repair/dA-tailing Module (catalog no. E7546, New England Biolabs). Ligation of adaptors was performed by adding Adaptor Mix (SQK-LSK114, Oxford Nanopore Technologies, Oxford, UK). The adaptor-ligated DNA was cleaned and quantified using fluorometry (Qubit 3.0, Thermo Fisher Scientific Inc., MA, USA) before library construction. The final library was sequenced on the Nanopore PromethION platform using an R10.4.1 flowcell (Oxford Nanopore Technologies). Basecalling was performed using Dorado (RRID) v. 0.3.4 [20].

For genome assembly, annotation, and comparative genome analysis, default parameters were used in all programs unless otherwise specified.

### Date preprocessing

Short WGS reads of *O. purpureiflora* and Hi-C reads from all 3 *Ormosia* species were quality-trimmed using Sickle v. 1.33 (RRID:SCR_006800) [21]. Reads with base quality values below 30 or lengths shorter than 80 bp were removed. The WGS reads of *O. purpureiflora* were further error corrected using RECKONER v. 1.1 [22]. Based on the error-corrected reads, 21-mer frequencies were generated using Jellyfish 2.3.0 (RRID:SCR_005491) [23], and the results were analyzed using GenomeScope 2.0 (RRID:SCR_017014) [24] to estimate the genome size, heterozygosity, and repetitiveness of *O. purpureiflora*. The ploidy level of *O. purpureiflora* was determined using nQuire with the “lrdmodel” function [25]. For ONT (ultra-)long WGS reads of *O. purpureiflora*, adapters were removed using Porchop 0.2.4 [26]. ONT reads larger than 20 kb were then extracted from the full dataset and defined as the 20 kb ONT read set, which was subsequently used for *O. purpureiflora* genome assembly.

### Genome assembly

Using the 20 kb ONT read set, the *O. purpureiflora* genome was assembled using NextDenovo 2.3.1 [27]. After assembly, Pseudohaploid [28] and Purge_Dups v. 1.2.6 (RRID:SCR_021173) [29] were used to identify and remove duplications resulting from heterozygosity. The assembly was then polished sequentially by Racon v. 1.5.0 (RRID:SCR_017642) [30] (run twice), Hapo-G v. 1.3.2 [31] (run twice), and Polypolish v. 0.5.0 [32]. Depthcharge v. 0.2.0 [33] was applied to correct potential misassemblies, and contigs shorter than 1,000 bp were removed. The corrected assembly was scaffolded using Hi-C reads with Scaffhic v. 1.1 [34], the Juicer pipeline 1.6 (RRID:SCR_017226) [35], and 3d-dna 201,008 (RRID:SCR_017227) [36]. Gaps in the scaffolded assembly were closed with TGS-GapCloser v. 1.2.1 (RRID:SCR_017633) [37]. The gap-closed assembly was polished again using Racon, Hapo-G, and Polypolish. Redundans 0.14a [38] was used to remove redundant sequences unanchored to chromosomes. The assembly was then uploaded to GenBank to check for possible contamination. Sequences identified as bacterial and fungal contaminants were removed. Subsequently, telomeric repeats at each chromosome ends were identified (with the parameter of “–motifs TTTAGGG –matchAny”) and recovered using Teloclip v. 0.0.3 [39]. The assembly was then polished by Racon, Hapo-G, and Polypolish to produce a complete genome assembly. To evaluate the assembly completeness, Benchmarking Universal Single-Copy Orthologs (BUSCO) v. 5.5.0 (RRID:SCR_015008) [40] was applied using the eudicots_odb10.2020–09-10 database, which contains 2,326 conserved eudicot core genes. Assembly quality was further assessed using AssemblyQC v. 2.1.1 [41] and GAEP v. 1.2.3 [42]. AssemblyQC provided metrics such as the long terminal repeat (LTR) Assembly Index (LAI) [43], which evaluates contiguity based on repetitive sequences, as well as *k*-mer-based assembly completeness [44]. GAEP offered mapping-based evaluations, reporting read mapping ratios for various read types (long WGS, short WGS, and RNA-seq reads) and a consensus quality value (QV) for overall mapping accuracy.

For *O. emarginata* and *O. semicastrata*, the primary assemblies [15] were upgraded to chromosome-scale assemblies following the *O. purpureiflora* procedures, starting from Hi-C scaffolding. The final assemblies were evaluated for quality by using the same methods applied to *O. purpureiflora*.

### Repeat sequence and gene prediction

The repeat sequences in 3 *Ormosia* chromosome-scale assemblies were identified using both EDTA v. 2.1.0 (RRID:SCR_022063) [45] and RED v. 2.0 [46]. The results from both programs for each assembly were combined and used to soft-mask the corresponding assembly with Bedtools v. 2.29.2 (RRID:SCR_006646) [47] using the commands “merge” and “maskfasta.” An explanation of the merging procedure is shown in Supplementary Fig. S1. The densities of repetitive elements measured by length proportion (percentage of sequence coverage on chromosome) and number per 10^5^ or 10^6^ bp on the chromosomes were then calculated with Circlize v. 0.4.15 [48] under the parameter of “overlap=FALSE.”

The soft-masked *Ormosia* assemblies were annotated using BRAKER2 v. 2.0 [49] and the Funannotate pipeline v. 1.8.16 [50]. BRAKER2 utilized RNA-seq reads and reference proteins from eight species (Supplementary Table S1) for transcriptome- and homology-based annotation, except for ab initio-based gene prediction. The results from BRAKER2 were integrated using Funannotate to generate consensus gene sets. Gene prediction in Funannotate followed 3 steps: “train,” “predict,” and “update.” For the “predict” and “update” steps, the parameters “-max_intronlen 100,000 -busco_db embryophyta -organism other” were applied. Function annotation of predicted genes in *Ormosia* species was performed using Funannotate with the “annotate” command. The annotation databases included dbCAN v. 10.0 (RRID:SCR_013208) [51], EggNOG v. 5.0.2 (RRID:SCR_002456) [52], Gene Ontology (GO, RRID:SCR_002811) [53, 54], Kyoto Encyclopedia of Genes and Genomes (KEGG, RRID:SCR_012773) [55], InterPro v. 5.62-94.0 (RRID:SCR_006695) [56], MEROPS v. 12.0 [57] (RRID:SCR_007777), Pfam v. 35.0 [58] (RRID:SCR_004726), SignalP 5.0b (RRID:SCR_015644) [59], and UniProt v. 2023_02 (RRID:SCR_002380) [60].

The completeness of the predicted genes was initially evaluated using BUSCO with the eudicots_odb10.2020-09-10 database, analyzing the longest transcripts from each *Ormosia* assembly. In addition, prediction quality was assessed using the online tool OMArk v. 0.3.0 [61]. Unlike BUSCO, which focuses solely on conserved single-copy genes, OMArk evaluates completeness based on conserved genes in both single and multiple copies. It also examines the consistency of the predicted genes relative to closely related species (e.g., the proportion of genes in the same lineage) and identifies potential contamination events. Finally, the completeness of the predicted genes was examined against 15,345 representative gene models from 12 Fabaceae species [62]. For this analysis, the gene models of each comparative species (Supplementary Table S2) were matched to the representative genes by using blastp (RRID:SCR_004870) v. 2.13.0 [63] with the parameters “-evalue 1e-2 -outfmt 6 -num_threads 96 -max_hsps 5 -max_target_seqs 5.”

For the comparative genomic analyses, only the longest transcript for each gene across all species was used, unless stated otherwise. Additionally, for genome comparisons, the protein-coding genes of all other species used in our phylogenetic analysis (see below) were functionally annotated following the same procedures applied to the *Ormosia* species.

### Gene family and comparative genomics

Orthologous groups (gene families) in *Ormosia* were identified using OrthoFinder 3.0.0 (RRID:SCR_017118) [64, 65], with protein-coding gene sequences from 17 other species (Supplementary Table S2) as inputs. Phylogenetic analysis was subsequently performed using 1,131 single-copy orthologs inferred using OrthoFinder, employing STAG [66] and STRIDE [67], which are integrated within OrthoFinder. The gene family file generated using OrthoFinder was further analyzed to assess gene family expansion or contraction using CAFE v. 5 (RRID:SCR_018924) [68]. The species tree, along with the divergence time required for CAFE analysis, was constructed using MCMCTree [69], with 12 calibration points from the TimeTree database (Supplementary Table S3) for calibration. Following the CAFE analysis, GO and KEGG enrichment analyses were performed on the significantly expanded and contracted gene families in *O. purpureiflora* using TBtools v. 2.030 [70].

### Gene duplications, synteny, and structural variation analysis

Ancient whole genome duplication (WGD) events in *Ormosia* and their sister species *Lupinus albus* (see Results in "Gene family" section) were identified using wgd v. 1.1.2 [71]. Gene duplications in *Ormosia* were analyzed using Doubletrouble v. 0.99.1 [72], which classified the duplication origin into categories including WGD, tandem duplications, proximal duplications, transposed duplications, and dispersed duplications [73]. In this analysis *L. albus* was used as an outgroup species. For genes resulting from WGD, tandem, and proximal duplications in *O. purpureiflora*, GO and KEGG enrichment analyses were performed using TBtools.

Syntenic regions within and between *Ormosia* and *L. albus* genome assemblies were identified using MCScanX [74] and visualized using Shinycircos [75] or SynVisio [76]. The parameter of “-s 30” (MATCH_SIZE) was used for synteny analysis in MCScanX. Structural variations were identified using chromeister v. 1.5.a [77] and plotsr v. 1.1.0 [78].

### Identification of nucleotide-binding leucine-rich repeats and other resistance genes

Nucleotide binding leucine-rich repeats (NLR) genes are the primary plant resistance (*R*) genes that protect against viruses, bacteria, nematodes, fungi, oomycetes, and insects [79, 80]. These genes typically consist of 3 canonical domains: a variable N-terminal domain, a central nucleotide-binding domain (NB-ARC), and a C-terminal domain composed of leucine-rich repeats (LRRs) [81]. At the N terminus, 3 types have been identified: Toll/interleukin-1 receptor (TIR), coiled-coil (CC), and resistance to powdery mildew8 (RPW8) [82]. The InterPro/Pfam entries associated with these domains include NB-ARC (IPR002182/PF00931), TIR (IPR000157/PF01582/PF13676), CC (IPR038005), RPW8 (IPR008808/PF05659), and LRR (IPR001611/PF00560/, IPR013101/PF07723, IPR011713/PF07725, IPR025875/PF12799, IPR026906/PF13306, IPR001611/PF13516/PF13855, PF14580 and IPR032675). In addition to the NLR genes, other *R* genes were identified based on their InterPro entries, as described by De-la-Cruz et al. [83]. Using gene annotation results from all species (3 *Ormosia* species and the 17 comparative species listed in Supplementary Table S2), obtained using the “annotate” command from the Funannotate pipeline, the InterPro/Pfam entries of their genes were matched to the corresponding *R* gene entries. The types and statistics of *R* genes were subsequently categorized for each species.

Because the above searches were mainly based on the InterPro and Pfam databases, both were generalized domain annotation tools and might provide overlapping or fragmented annotations, leading to inaccurate results in *R* gene identification. Therefore, NLR genes, in *Ormosia* and the other compared species in our phylogenic analysis, were also identified by Resistify v. 1.1.5 [84], which could accurately and extensively identify and classify them by integrating different programs and more filtering steps.

### Transcription factor

Transcription factor (TF) genes in the genomes of *Ormoisa* species and the other species in our phylogenic analysis were identified by the TF prediction online tool PlantTFDB v. 5.0 [85]. TF genes were also predicted by TransFacPred [86], which combined alignment-free (machine learning method) and alignment-based (BLAST method) methods to achieve high accuracy.

### Single-nucleotide polymorphism calling

Single-nucleotide polymorphisms (SNPs) in 153 *O. purpureiflora* individuals were identified using NGSEP (RRID:SCR_012827) v. 5.0.0 [87] with the *O. purpureiflora* genome assembled in this study serving as the reference. The mapping results used for this procedure were from BWA v. 0.7.17-r1188 [88]. For NGSEP, the parameters -h 0.00952 –maxAlnsPerStartPos 2 were used, with all other settings remaining at their defaults. The -h parameter specifies the heterozygosity rate, which was derived from the GenomeScope results (see Results in "Genome assembly" section). The raw SNPs called by NGSEP were quality-filtered using VCFtools (RRID) v. 0.1.17 [89], with the parameters of “–max-missing 0.95 –maf 0.05 –recode –recode-INFO-all –min-meanDP 20 –mac 3 –minQ 30 –non-ref-af 0.001 –max-non-ref-af 0.9999.” Filtered SNPs were further processed to remove the SNPs deviating from Hardy–Weinberg equilibrium (HWE) and the InDels. Departure from HWE can cause genotyping errors due to the presence of null alleles, sequence duplication, copy number variation, and other sequencing problems related to read depth. The HWE filtering was performed using the script “filter_hwe_by_pop.pl” from the “SNP Filtering Tutorial” [90] with the parameter “-c 0.”

To accurately infer population genetic diversity and structure, SNPs called by NGSEP were further filtered to remove those in linkage disequilibrium (LD) by using Plink (RRID) v. 1.90p [91–93]. Specifically, SNP loci with an LD association coefficient (*r*²) greater than 0.2 were excluded. Finally, outlier SNPs (potentially under selection) were identified and removed from the dataset used for genetic diversity and structure inferences. These SNPs were detected using PCAdapt v. 4.3.5 [94, 95] and BayPass v. 2.4 [96].

For PCAdapt, a principal component analysis (PCA) was first performed, and a Scree plot was used to determine the optimal number of PCs for regression with each SNP. Following regression analysis, SNPs with a *q* value (adjusted *P* value) of <0.01 were considered outliers. For BayPass, the core model with default parameters was applied. This model estimated an *F*_ST_-like XtX statistic while accounting for the variance–covariance structure. To determine significance, a calibrated threshold (99%) was established by simulating pseudo-observed datasets (100,000 SNPs). SNPs falling within the 99.9% quantile of the pseudo-observed XTX distribution were considered outliers. Outlier SNPs were identified as those occurring in both PCAdapt and BayPass results.

### Genetic diversity and genetic structure

Genetic diversity parameters, including observed heterozygosity (*Ho*), expected heterozygosity (*He*), and inbreeding coefficient (*Fis*), were estimated by VCFtools. Nucleotide diversity within populations (*π*), nucleotide divergence between populations (*dxy*), and pairwise genetic differentiation (*Fst*) were calculated using pixy v. 1.2.7.beta1 [97].

The genetic structure of *O. purpureiflora* was inferred through PCA and ADMIXTURE (RRID:SCR_001263) [98]. PCA was performed using SNPRelate v. 1.36.0 [99], and ADMIXTURE was conducted using the AdmixPipe v. 3.2 pipeline [100]. In AdmixPipe, the number of potential genetic groups (*K*) was tested from 1 to 6, with 20 replicates for each *K* value. The best *K* value was determined based on cross-validation (CV) errors. For the inferred *K*, CLUMPAK v. 1.1 [101] was used to estimate the mean membership coefficients for individuals across the 20 replicates.

## Results

### Chromosome number

The ploidy level estimated using nQuire indicated that the *O. purpureiflora* genome is diploid because the diploid model showed a lower delta likelihood than the free model (diploid delta likelihood: 1,609,982.99; triploid delta likelihood: 2,029,931.52; tetraploid delta likelihood: 2,270,455.24) (Supplementary Table S4). Similar estimations for *O. emarginata* and *O. semicastrata* confirmed that these species also have diploid genomes.

The chromosome number of *O. purpureiflora* was determined to be 2*n* = 16 (Supplementary Fig. S2), consistent with the number reported previously in *O. macrocalyx* [102] and *O. arborea* [103].

### Genome sequencing

For *O. purpureiflora*, the ONT sequencing platform generated approximately 181.6 Gb of WGS reads, including 51.3 Gb of ultra-long reads. The short sequencing platform produced approximately 139.3 Gb WGS reads and 146.8 Gb Hi-C reads. RNA-seq data amounted to approximately 20.4 Gb, 21.9 Gb, 23.3 Gb, and 25.3 Gb for leaf, flower, fruit, and seed samples, respectively. For *O. emarginata* and *O. semicastrata*, 148.7 Gb and 123.6 Gb Hi-C reads were generated, respectively.

### Genome assembly

**Figure 3:**
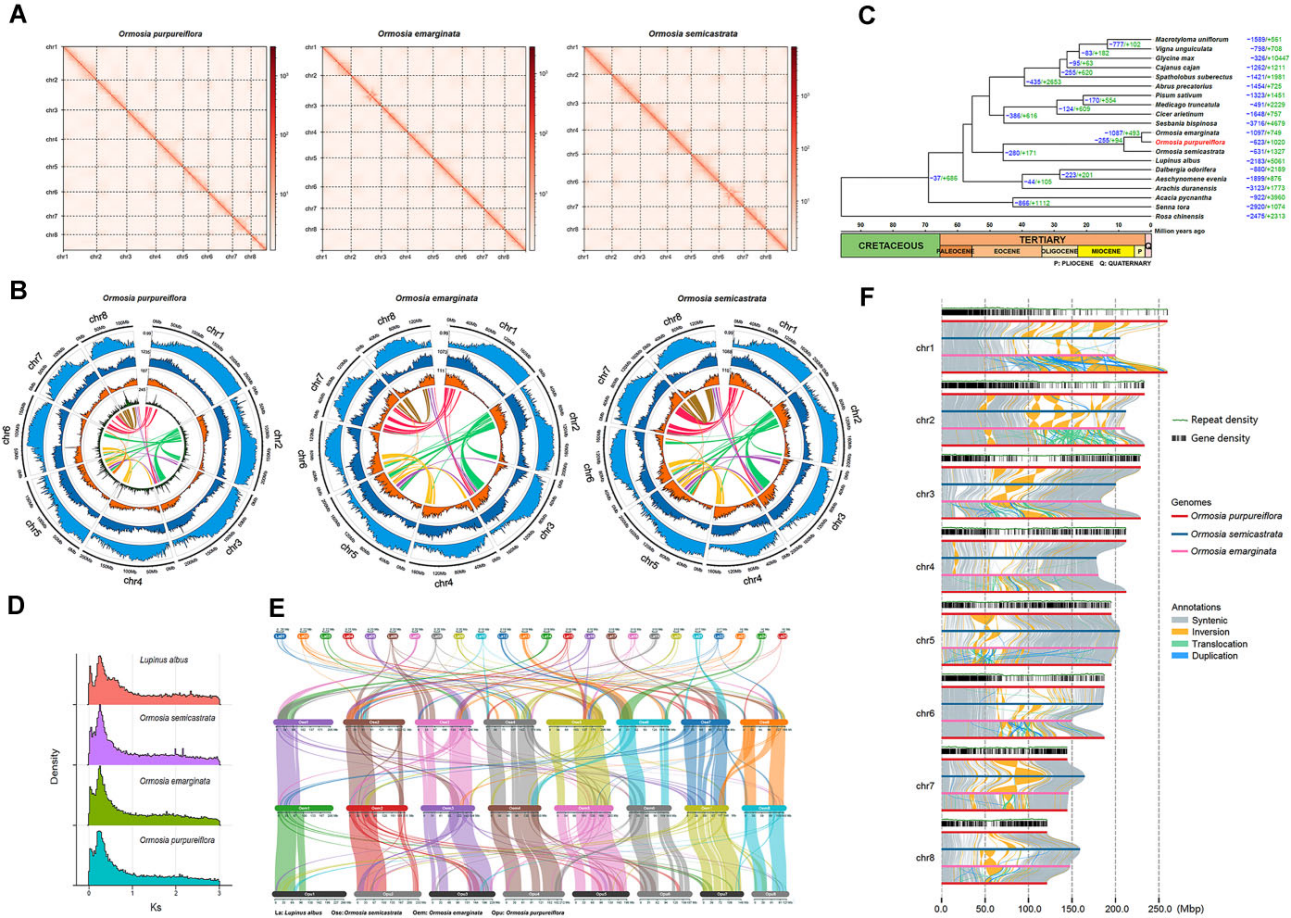
*Ormosia* genomes and comparative genomics. (A) Hi-C interaction heat maps (bin length, 100,000 bp) for the genome assemblies of three *Ormosia* species. (B) Circos plot showing the genome features (chromosome, repeat density in length proportions, repeat density in numbers, gene density, and syntenic blocks from outer to inner) across chromosomes of the genome assemblies of three *Ormosia* species. Repeat densities in each Circos plot was quantified by all repetitive elements. For *O. purpureiflora*, the Circos plot also includes SNP density results between the results of gene density and syntenic blocks. All densities were estimated using a 1 Mbp sliding window. (C) The inferred phylogenetic tree, divergence time, and contracted (–) and expanded (+) gene families in *O. purpureiflora* and other species. (D) The density distribution of synonymous nucleotide substitutions (*Ks*) in the whole genome duplication analysis for *Ormosia* species and their sister species, *L. albus*. (E) Syntenic blocks among *Ormosia* species and *L. albus*. (F) Intrachromosomal structural variations observed among the three *Ormosia* species.

For *O. purpureiflora*, the genome size estimated using GenomeScope was 1,503,292,231 bp, with repetitive sequences accounting for 66.6% of the genome and a heterozygosity rate of 0.952% (Supplementary Fig. S3). The initial genome assembly size was 1,811,176,403 bp, comprising 313 contigs with an N50 of 50,908,349 bp. After redundancy removal, Hi-C scaffolding and gap closing, the final assembly measured 1,584,128,722, with 1,583,483,254 bp (99.96%) anchored to 8 chromosomes (Table 2, Fig. 3A), consistent with chromosome number observation (Supplementary Fig. S2). The longest chromosome was 259,935,025 bp long, and the shortest was 121,398,155 bp.

**Table 2:** Statistics and evaluations of genome assemblies for three *Ormosia* species

Species	*O. purpureiflora*	*O. emarginata* ^a^	*O. semicastrata* ^a^
Initial assembly statistic (bp)			
N10	122,192,683	81,285,628	89,031,100
N20	120,000,233	63,464,384	79,796,434
N30	75,858,835	43,593,171	73,253,298
N40	61,354,201	37,463,220	56,807,054
N50	50,908,349	28,195,512	48,976,089
N60	45,450,924	25,800,464	45,239,136
N70	36,587,725	20,527,781	31,722,207
N80	15,728,371	13,438,452	22,051,163
N90	3,163,854	7,895,810	12,933,450
N100	34,487	173,104	128,272
Total length	1,811,176,403	1,420,917,605	1,511,766,959
Average length	5,786,506.08	15,787,973.39	23,996,300.94
Largest length	142,757,542	84,853,091	144,833,628
Minimum length	34,487	173,104	128,272
Number of contigs	313	90	63
Assembly after applying Hi-C data (bp)			
chr1	259,935,025	199,918,031	205,218,018
chr2	233,292,245	210,768,611	211,883,283
chr3	229,093,642	183,696,964	200,464,886
chr4	212,222,348	180,298,008	178,099,194
chr5	195,349,128	202,609,791	205,007,630
chr6	187,433,795	149,243,870	185,806,757
chr7	144,758,916	145,867,561	164,432,676
chr8	121,398,155	147,815,325	159,254,978
Unanchored to chromosome	645,468	35,505	519,897
Total length	1,584,128,722	1,420,253,666	1,510,687,319
Assembly quality assessed by AssemblyQC			
LAI	16.08	13.66	17.56
*k*-mer based assessment			
Completeness	88.36%	78.04%	81.15%
QV	28.83	27.02	28.34
Assembly quality assessed by GAEP			
GC content	35.06%	34.53%	34.63%
Mapping based assessment			
QV	39.74	37.38	38.46
Long WGS reads mapping ratio	97.32%^b^	99.72%^c^	99.59%^c^
Short WGS read mapping ratio^d^	99.76%	98.31%	98.43%
RNA-seq mapping ratio^e^			
Leaf	95.77%	93.52%	95.24%
Flower	91.15%	—	—
Fruit	95.96%	—	—
Seed	92.23%	—	—

a From Liu et al. [15].

b With reads longer than 20 kb

c With reads longer than 10 kb.

d Trimmed and error-corrected.

e Trimmed using trimmomatic (RRID:SCR_011848) v. 0.39 [121] with parameter “SLIDINGWINDOW:4:5 LEADING:5 TRAILING:5 MINLEN:25.”

The initial assemblies for *O. emarginata* and *O. semicastrata* were 1,420,917,605 bp and 1,511,766,959 bp, respectively [15]. GenomeScope estimations using a *k*-mer size of 21 revealed repeat contents of 65.5% and 63.4%, and heterozygosity rates of 2.29% and 2.05% for *O. emarginata* and *O. semicastrata*, respectively. Both species displayed higher heterozygosity than *O. purpureiflora*, although the repeat content was similar across the 3 species. After incorporating Hi-C data, the assembly sizes of *O. emarginata* and *O. semicastrata* were refined to 1,420,253,666 and 1,510,687,319 bp, respectively (Table 2). Each assembly achieved 8 chromosome-level scaffolds, accounting for 99.99% and 99.97% of the total in *O. emarginata* and *O. semicastrata*, respectively.

For *O. purpureiflora*, BUSCO evaluation revealed 98.3% complete BUSCOs, with 89.4% of them being single-copy BUSCOs and 8.9% being duplicated BUSCOs. In addition, 0.3% BUSCOs were fragmented, and 1.4% were missing. For *O. emarginata*, the complete BUSCO score was 97.0%, including 89.4% complete and single-copy BUSCOs and 7.6% complete but duplicated BUSCOs, with 0.5% fragmented and 2.5% missing. Similarly, *O. semicastrata* achieved a complete BUSCO score of 98.4%, consisting of 90.4% complete and single-copy BUSCOs and 8.0% complete but duplicated BUSCOs, with fragmented and missing BUSCOs constituting 0.1% and 1.5%, respectively.

The LAI values for the 3 *Ormosia* assemblies were all above 10, ranging from 13.66 to 17.56 (Table 2), meeting the quality standard for a “reference genome.” Mapping-based evaluations indicated that all types of reads achieved high mapping ratios, exceeding 91%. However, the *k*-mer and mapping-based quality value (QV) scores in all 3 *Ormosia* assemblies were below 40, a threshold that corresponds to 99.99% base accuracy and is considered high quality for genome assemblies [42, 44]. In addition, *k*-mer-based completeness was below 90% across all *Ormosia* assemblies, with *O. purpureiflora* having the highest completeness at 88.36% and *O. emarginata* having the lowest at 78.04%. These assessments suggest that further improvements in the assemblies are warranted.

Assembling genomes with a large size (>1 Gb), high repeat content (>50%), and elevated heterozygosity (>0.5%) presents significant challenges [104], and the species examined in this study exhibited all these features. To address these issues, we used the Nanopore sequencing platform, which generates reads that are longer in length than those produced by the PacBio sequencing platform, particularly in Hi-Fi sequencing mode [105]. For *O. purpureiflora*, we included ultra-long reads (>50 kb) to enhance assembly continuity. In the assembly process, reads longer than 20 kb were used for assembling the *O. purpureiflora* genome, whereas reads longer than 10 kb were used for assembling the genomes of *O. emarginata* and *O. semicastrata*. Programs such as Pseudohaploid and Purge_Dups were used to remove heterozygous contigs and regions, effectively mitigating challenges associated with high repeat content and heterozygosity in these genomes. However, ONT reads generally have high sequencing error rates, ranging from 5% to 20% [105–107]. For *O. purpureiflora*, the error rate of ONT reads was 15.18%, as assessed using the 20 kb ONT read set used for genome assembly (see Methods in "Genome assembly" section). Similarly, error rates of 17.75% and 16.82% were observed in the 10 kb ONT read sets of *O. emarginata* and *O. semicastrata*, respectively. Given these limitations, haplotype-resolved de novo genome assembly was not performed for the 3 *Ormosia* species. Future studies should incorporate highly accurate Hi-Fi long-read sequencing technology and phasing steps to optimize the current assemblies and improve their overall quality.

### Repeat and gene annotation

RED analyses identified 1,037,006,095 bp (65.5%), 885,912,252 bp (62.4%), and 968,176,023 bp (64.1%) of repetitive sequences in *O. purpureiflora, O. emarginata*, and *O. semicastrata*, respectively. EDTA analyses revealed higher percentages, that is, 1,139,417,595 bp (71.9%), 989,514,254 bp (69.6%), and 1,074,353,470 bp (71.1%) of repetitive sequences in *O. purpureiflora, O. emarginata*, and *O. semicastrata*, respectively (Supplementary Table S5). After combining the results from RED and EDTA, the total repetitive components were found to be 1,209,324,791 bp (76.3%) in *O. purpureiflora*, 1,051,218,280 bp (74.0%) in *O. emarginata*, and 1,135,447,010 bp (75.2%) in *O. semicastrata*. According to EDTA analyses, the *Gypsy*-like long terminal repeat retrotransposon (LTR-RT) family represents the most abundant repetitive sequence, comprising 33.51%, 35.45%, and 27.73% of the genome assemblies for *O. purpureiflora, O. emarginata*, and *O. semicastrata*, respectively.

**Figure 4:**
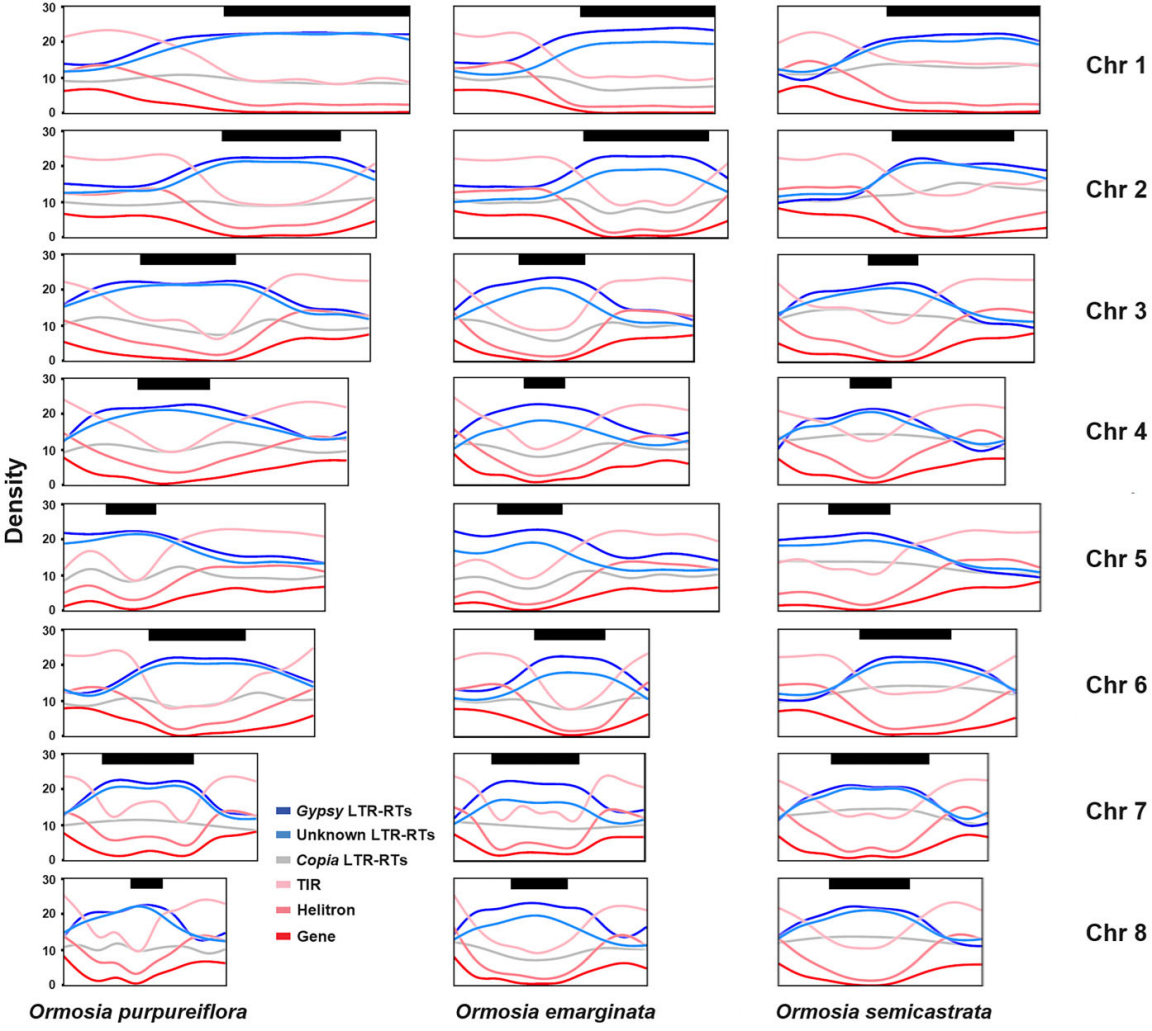
Smoothing lines for gene and repeat density distributions (bin size, 100,000 bp) along chromosomes in *Ormosia* species. The repeat densities were measured by the number of each repetitive element. Scatterplots for the gene density and repeat distribution are presented in Supplementary Fig. S4. Box sizes correspond to chromosome sizes in *Ormosia* species, whereas the black bars on the upper part of each box (chromosome) represent the hot structural rearrangement region in the chromosomes.

Overall, a “complementary” pattern was observed in the density distributions of repetitive elements between their length proportions and numbers on the chromosomes in *Ormosia*, namely two types of distribution concentrated on different parts of the same chromosomes (Fig. 3B). In numbers, a closer examination revealed that the types of Helitrons and terminal inverted repeats (TIRs) generally distributed disparately from the types of *Gypsy* and unknown LTR-RTs (Fig. 4 and Supplementary Fig. S4), whereas the other LTR-RT type, *Copia*, was generally evenly distributed along the chromosomes in *Ormosia*.

Gene prediction identified 55,061 genes encoding 59,809 proteins in *O. purpureiflora*. For *O. emarginata* and *O. semicastrata*, the predictions revealed 50,517 and 51,220 genes encoding 54,456 and 55,363 proteins, respectively (Table 3). Table 3 also provides statistics on various gene features in the 3 species’ assemblies. Overall, *O. purpureiflora* exhibited the lowest average number of exons and introns per gene as well as the shortest average gene and CDS lengths. Approximately 70.81%, 76.43%, and 72.43% of protein-coding genes in *O. purpureiflora, O. emarginata*, and *O. semicastrata*, respectively, were functionally annotated in at least one database (Table 3). Comparatively low annotation rates were also observed in other genomes, such as *Senna tora* (67.16%), *Pisum sativum* (72.70%), and *Sesbania bispinosa* (78.15%; Supplementary Table S6). By contrast, higher annotation rates were reported in agriculturally important species such as *Glycine max* (99.03%), *Cajanus cajan* (98.97%), *Cicer arietinum* (98.84%), and *Vigna unguiculata* (98.55%), whose genomes have received greater research attention, contributing to more functional information in annotation databases. The low annotation rates in *Ormosia* species may be attributed to the presence of novel genes with unknown functions, which are less represented in the current annotation databases.

**Table 3:** Statistics of predicted genes for three *Ormosia* species^a^

Species	*O. purpureiflora*	*O. emarginata*	*O. semicastrata*
Predicted gene information			
No. of protein-coding genes	55,061	50,517	51,220
No. of mRNAs	59,809	54,456	55,363
No. of exons	254,087	241,766	245,306
No. of CDSs	242,624	232,457	235,681
No. of 5′ UTRs	32,588	25,531	27,197
No. of introns	193,432	186,540	189,213
No. of 3′ UTRs	30,110	23,826	25,220
Genes			
Average gene length (bp)	2,991.06	3,277.60	3,356.76
Largest length of genes (bp)	423,361	494,687	267,325
Minimum length of genes (bp)	141	153	153
50% cumulative length of genes (bp)	1,467	1,636	1,710
90% cumulative length of genes (bp)	7,312	7,422	7,533
Exons in genes			
Average exons per gene	3.81	4.03	4.03
Average exon length (bp)	226.87	218.64	222.94
Largest length of exons (bp)	8,728	7,959	7,959
Minimum length of exons (bp)	3	3	3
50% cumulative length of exons (bp)	159	149	151
90% cumulative length of exons (bp)	658	581	603
Introns in genes			
Average introns per gene	2.81	3.03	3.03
Average intro length (bp)	757.16	790.41	811.46
Largest length of introns (bp)	422,767	783,472	318,269
Minimum length of introns (bp)	11	11	11
50% cumulative length of introns (bp)	229	234	246
90% cumulative length of introns (bp)	1,375	1,419	1,463
CDS in genes			
Average CDS length (bp)	864.15	881.45	898.37
Largest length of CDSs (bp)	16,359	15,351	16,323
Minimum length of CDSs (bp)	141	150	144
50% cumulative length of CDSs (bp)	609	621	642
90% cumulative length of CDSs (bp)	1941	1953	1917
Gene functional annotations using different databases			
dbCAN	1,671	1,538	1,596
EggNOG	41,143	38,192	38,955
KEGG	20,284	19,163	19,545
GO	29,006	27,167	27,824
InterPro	35,255	32,777	33,548
MEROPS	1,335	1,269	1,292
Pfam	28,007	26,150	27,091
SignalP	4,143	3,778	3,952
UniProt	9,517	8,988	9,234
Total	42,348	39,147	40,100

a Using all transcripts.

Gene prediction completeness, as assessed using BUSCO, indicated a completeness score of 96.1% in *O. purpureiflora* (88.8% complete and single-copy, 7.3% complete but duplicated), with 1.5% fragmented and 2.4% missing genes. For *O. emarginata*, the BUSCO analysis revealed 95.1% completeness (88.6% complete and single-copy, 6.5% complete but duplicated), with 1.5% fragmented and 3.4% missing genes. In *O. semicastrata*, the completeness score was 96.3% (89.6% complete and single-copy, 6.7% complete but duplicated), with 1.4% fragmented and 2.3% missing genes.

OMArk evaluations reported a completeness score of 97.9% for *O. purpureiflora* (67.0% single-copy, 30.0% duplicated), with 2.1% missing genes. Of the predicted genes, 60.4% were consistent, 3.2% were inconsistent, and 36.39% are unknown (Supplementary Table S7). For *O. emarginata*, OMArk indicated 97.1% completeness (67.0% single-copy, 30.0% duplicated), with 2.9% missing; 62.8% consistent, 2.5% inconsistent, and 34.66% unknown genes. For *O. semicastrata*, OMArk reported 98.1% completeness (67.8% single-copy, 30.3% duplicated), with 1.9% missing, 62.8% consistent, 2.8% inconsistent, and 34.37% unknown genes. No contamination was detected in the gene sets of any of the 3 *Ormosia* species. Compared with other species, *Ormosia* exhibited a higher proportion of duplicated and unknown genes and lower consistency. Similarly high levels of duplicated genes were observed in *Ormosia*’s sister species, *L. albus* (37.77%), which may be attributed to lineage-specific WGD events (see below the results in "Gene duplications, synteny, and structural variation analysis" section). The low consistency scores are likely linked to the high proportion of unknown genes. The high proportion of unknown genes in *Ormosia* may result from newly identified genes that lack homologs in OMArk's reference databases, reflecting the limited genomic information available for this lineage. A similar trend of high unknown gene rates (39.07%) and low consistency (56.99%) in *S. tora* may also be explained by the same factor.

The *Ormosia* genes showed high matching rates with Fabaceae representative genes (Supplementary Table S8), ranging from 73.41% to 73.91%. These rates were only slightly lower than those observed for *Medicago truncatula* (75.68%) and *Pisum sativum* (75.36%), supporting the completeness of the predicted *Ormosia* genes.

According to InterPro functional annotation, we found that some photosynthesis-related genes were not annotated in *O. purpureiflora* when compared with *O. emarginata* and *O. semicastrata* (Supplementary Table S9). Specifically, the number of genes associated with Photosystem I PsaA/PsaB (IPR001280) in *O. purpureiflora* was 3, which was lower than the number of genes in *O. emarginata* (8) and *O. semicastrata* (9). In addition, the InterPro database showed the absence of annotation in several genes related to plant–pathogen interaction (EDS1-like, IPR044214), plant reproduction (DBP10, C-terminal, IPR012541), pyrimidine/nucleotide metabolism (deoxyuridine triphosphate nucleotidohydrolase, IPR008181; dUTPase-like, IPR029054/IPR036157), regeneration (Thioredoxin DCC1, IPR044691), seed maturation protein 1 (SMP1, IPR044984), and nodulin (IPR003387) in *O. purpureiflora*.

### Gene family

A total of 47,608 gene families were identified using OrthoFinder. In *O. purpureiflora*, 50,275 genes (91.3%) were assigned to 27,347 gene families. Among these, 454 families were specific to *O. purpureiflora* (Supplementary Table S10). The genes in these families were mainly enriched in processes such as endoplasmic reticulum to Golgi vesicle-mediated transport and non-membrane-bounded organelle assembly in GO’s BP category (Supplementary Table S11) and ribosome biogenesis in eukaryotes in the KEGG analysis (Supplementary Table S12).

The phylogenetic tree (Fig. 3C) indicated that *O. purpureiflora* was sister to *O. emarginata* and that *Ormosia* was sister to *L. albus*. The estimated divergence time between *O. purpureiflora* and *O. emarginata* was approximately 2.94 million years ago (95% CI: 1.19–5.00), whereas the divergence time between *Ormosia* and *Lupinus* was 45.90 million years ago (95% CI: 32.66, 56.44). In *O. purpureiflora*, 1,020 gene families were expanded and 623 were contracted. Among these, the expansion and contraction were significant in 205 and 84 gene families (*P* < 0.05). Significantly expanded gene families were mainly enriched in DNA integration and regulation of amino acid transmembrane transport in GO’s biological process (BP) category (Supplementary Table S13) and alkaloid, polyketide, and zeatin biosynthesis in the KEGG analysis (Supplementary Table S14). The significantly contracted gene families were mainly associated with transcription by lipid transport and lipid localization in the GO’s BP category (Supplementary Table S15) and with terpenoid biosynthesis in the KEGG analysis (Supplementary Table S16).

The genes in the contracted gene families related to terpenoid biosynthesis were primarily cytochrome P450 (CYP450) genes, which are responsible for downstream activities in the final terpenoid products [108, 109]. However, terpenoids were mostly represented by two conserved domains with Pfam IDs of PF01397 and PF03936 [110]. A comparison showed that the *O. purpureiflora* assembly annotated 23 and 25 of these genes, slightly fewer than those in *O. emarginata* (31 and 26) and *O. semicastrata* (26 and 28). Nevertheless, the number of genes in *Ormosia* species was much higher than that in their sister species, *L. albus* (8 and 10).

### Gene duplications, synteny, and structural variation analysis

WGD analysis indicated that *O. purpureiflora* has undergone 1 WGD event (Fig. 3D), which was shared with the other 2 *Ormosia* species and *L. albus*. Therefore, this WGD event is not specific to *Ormosia* but instead may be specific to the Genistoid lineage in Fabales [16, 111]. Future studies, including newly published Fabaceae genomes, will help confirm this hypothesis.

Gene duplication analysis revealed that the 3 *Ormosia* species exhibited similar numbers of genes across different duplication types (Supplementary Table S17). In *O. purpureiflora*, enrichment analysis showed that WGD-duplicated genes were primarily associated with the processes related to calcium ion, blue light, flower, and development, and cytokinin biosynthetic process in the GO’s BP category (Supplementary Table S18). In KEGG analysis, these genes were linked to signaling proteins, glycosylphosphatidylinositol (GPI)-anchored proteins, GTP-binding proteins, and SNARE interactions in vesicular transport (Supplementary Table S19). Tandem-duplicated genes were mainly associated with phloem development, glutathione metabolic process, and the biosynthesis of monoterpenoid, anthocyanin, zeatin, and flavonoid (Supplementary Tables S20 and S21). Proximal-duplicated genes were predominantly involved in diterpenoid and triterpenoid biosynthetic process, arginine biosynthetic process, phloem development, and flavone and flavone biosynthesis (Supplementary Tables S22 and S23). These results were consistent with those of a previous study on *O. emarginata* and *O. semicastrata* by Liu et al. [15], which showed that tandem and proximal duplicated genes were relevant to various (secondary) biosynthetic and metabolic processes, including the biosynthesis of alkaloid, flavonoid, and terpenoid.

Synteny analysis within *Ormosia* revealed 48, 42, and 45 syntenic blocks in *O. purpureiflora, O. emarginata*, and *O. semicastrata*, respectively (Supplementary Table S24). The longest syntenic blocks identified in these species were between chromosomes 2 and 3. These blocks measured 39,614,256 bp and contained 427 gene pairs in *O. purpureiflora*, 33,895,706 bp with 383 gene pairs in *O. emarginata*, and 36,266,649 bp with 424 gene pairs in *O. semicastrata*. The syntenic relationships were illustrated in a Circos plot (Fig. 3B).

Overall, the *O. purpureiflora* genome exhibited highly syntenic relationships with the other two *Ormosia* genomes, as shown by both synteny analysis (Fig. 3E) and dot plots (Supplementary Fig. S5). However, further genetic variation analysis revealed extensive intrachromosomal rearrangements among the *Ormosia* species (Fig. 3F). These rearrangements were primarily concentrated in specific “hot” chromosomal regions, where the gene density was low, indicating unstable genome architecture in these regions, while gene-rich regions maintained a more conserved genome structure in *Ormosia*.

*O. semicastrata* exhibited greater divergence from *O. purpureiflora* and *O. emarginata*, as evidenced by the high unaligned proportions (52.36% unalignment with *O. purpureiflora* assembly and 50.54% unalignment with *O. emarginata* assembly) compared with lower unaligned proportions between *O. emarginata* and *O. purpureiflora* (24.88% and 28.51%, respectively, Supplementary Table S25). Furthermore, *O. semicastrata* exhibited fewer translocations and duplications than *O. purpureiflora* and *O. emarginata*. These findings align with those of our phylogeny analysis (Fig. 3C) as well as previous results, which have reported that *O. emarginata* and *O. semicastrata* belong to different clades [1]. Although *O. purpureiflora* was sister to *O. emarginata* and thus in the same clade, the structural rearrangements observed between *O. emarginata* and *O. semicastrata* were not preserved in *O. purpureiflora*.

*O. purpureiflora* and *O. emarginata* exhibited the largest inversion on Chromosome 1, spanning from 166,804,741 to 222,962,103 bp in *O. purpureiflora* and from 127,118,909 to 186,491,244 bp in *O. emarginata*. Extensive duplications were also observed on the same chromosome. Notably, a *O. purpureiflora*-specific inverted region was identified on Chromosome 2, spanning from 52,506,652 to 61,757,520 bp (Fig. 3F and Supplementary Fig. S5), which was located away from the rearrangement hot regions. This inverted region was 9,250,868 bp in length and contained 577 genes. The enrichment analysis of these genes revealed their involvement in osmotic stress and temperature regulation (Supplementary Table S26), which may contribute to *O. purpureiflora*’s adaptation to rocky environments (Fig. 1F), thin soil layers (with low soil moisture content), and relatively high elevation (400–750 m in altitude) [11].

### Identification of nucleotide-binding leucine-rich repeat and other *R* genes

Compared with other species in our phylogenetic analysis (Fig. 3C), *Ormosia* species were found to have a higher number of *R* genes (Supplementary Table S27). For the nucleotide-binding leucine-rich repeat (NLR) genes, their numbers were 1,269–1,346 in 3 *Ormosia* species by the Intrepro and Pfam databases searching, and 276–298 by the Resistify program. The results were higher than those of 10–12 compared species. However, when looking at the percentages of the NLR genes in *Ormosia*, they were not high in both databases searching and Resistify identifying results.

Specifically, the number and percentage of NLR genes in the *Ormosia* species were higher than those in the sister species *L. albus*. However, in terms of the other *R* genes, *L. albus* displayed a higher number and percentage than the *Ormosia* species. Among the *Ormosia* species, *O. purpureiflora* had a higher number and percentage of other *R* genes than *O. emarginata* and *O. semicastrata*. The distribution of *R* genes across the chromosomes of each *Ormosia* species is shown in Supplementary Fig. S6. *R* genes were spread across all 8 chromosomes, following a distribution pattern consistent with the overall gene distribution in *Ormosia* species.

### Transcription factor

Identified with PlantTFDB*, Ormosia* species had a higher number of TF genes than all the other species in our phylogenetic analysis (Fig. 3C), except for *Glycine max, Sesbania bispinosa, Acacia pycnantha*, and their sister species *L. albus*. However, the percentage of TF genes in *Ormosia* species was relatively low when compared with the species in the phylogeny, particularly in *O. purpureiflora* (3.96%, the lowest one; Supplementary Table S28). Detection with TransFacPred confirmed the high numbers but low percentage of TF genes in *Ormosia*.

### SNP calling

The raw SNPs called by NGSEP identified 37,875,127 loci, comprising 24,941,612 SNPs and 12,933,515 InDels. After quality filtering and InDel removal, 358,992 SNPs were retained. In total, 272,941 loci were identified as deviations from HWE and removed. Further LD filtering retained 40,146 loci. PCAdapt analysis indicated that five main components were suitable to account for population structure, as shown in the Scree plot (Supplementary Fig. S7). PCAdapt identified 5,814 outlier SNPs, whereas BayPass revealed 1,097 candidate SNPs. Across both analyses, 109 SNPs were identified as outliers. By removing these outlier SNPs, 40,037 SNPs remained as neutral loci for population genetics analyses.

### Genetic diversity and genetic structure

Genetic diversity analyses in *O. purpureiflora* (sub)populations indicated that all (sub)populations displayed similar levels of genetic diversity, and LFS4 exhibited the highest genetic diversity for 3 parameters (*Ho, He*, and *π*) (Table 1). Four (sub)populations showed close to zero *Fis*, indicating random mating status in them. Two (sub)populations, LFS1 and LFS4, displayed low negative *Fis* values, indicating excess heterozygosity. The overall *Fst* was 0.107, suggesting relatively high population differentiation. Compared with *O. henryi*, a species more widely distributed in southern China, *O. purpureiflora* displayed lower genetic diversity in the values of *Ho* and *He*, but not in *π*. For *O. henryi*, the genetic diversity measures were *Ho*: 0.228–0.287, *He*: 0.237–0.290, *π*: 0.122–0.143, and *Fis*: −0.023–0.022 in) [112].

Heterozygosity excess in plants may be attributed to several factors, including polyploidy, reproduction mode (such as outcrossing, self-incompatibility systems, and clonal growth), demographic history (such as population bottlenecks), and natural selection (e.g., the overdominant phenomenon, where heterozygous individuals have high survival rates) [113–120]. *O. purpureiflora* is a diploid species, as mentioned earlier. Its flowers are insect-pollinated, exhibiting a typical outcrossing reproductive system. Given the small size of its populations, the limited reproduction among individuals reduces the likelihood of inbreeding and leads to a decrease in inbred offspring. Therefore, the reproductive system may favor heterozygous individuals in the population. *O. purpureiflora* also reproduces asexually through suckering [11], which contributes to the observed heterozygosity excess in some (sub)populations. However, the effects of other factors, such as a historical bottleneck, cannot be overlooked and warrant further investigation.

PCA revealed that the first principal component generally divided LFS4 from the other (sub)populations (Fig. 2B). The second principal component further separated NKS from the others. The third principal component indicated the divergence in some LFS5 individuals. In the ADMIXTURE analysis, the cross-validation (CV) error decreased consistently from *K* = 1 to *K* = 6 (Supplementary Fig. S8), but from *K* = 4 onward, the decrease slowed down. Therefore, *K* = 4 was identified as the optimal number of genetic groups. Given the limited number of (sub)populations in *O. purpureiflora*, the present study reports the results for *K* = 2 to *K* = 6 (Fig. 2C). When *K* = 2, individuals in LFS4 were separated from the others. At *K* = 3, individuals in NKS were further separated. At *K* = 4, LFS5 was separated as a distinct group. When *K* was increased further, LFS2 was separated from the others and extensive admixture was observed in all LFS (sub)populations. Both PCA and ADMIXTURE analyses highlighted the distinctiveness of LFS4, although the reasons for this distinctiveness remain unclear.

## Conclusion

Fabaceae play a crucial role in biological nitrogen fixation and serve as a source of nutrition for wild fauna, contributing to the health and balance of ecosystems. The same holds true for *Ormosia* species. Previous studies have shown that *Ormosia* species are rich in secondary metabolites, including alkaloids, terpenes, and flavonoids, which warrant further exploration, particularly from a genomic perspective. The genomes of the two previous *Ormosia* species and the current *O. purpureiflora* genome indicate that genes involved in the biosynthesis of these metabolites are often found in tandem duplications, proximal duplications, or are expanded. The association between gene distribution and repeats suggests that these repeats play a role in gene duplication, highlighting the need for future research on this topic. Thus, the high-quality *Ormosia* genomes serve as a valuable resource for understanding the efficiency of metabolite biosynthesis and identifying potentially useful chromosome regions (such as syntenic regions and structural rearrangements) for future study.

## Supplementary Material

giaf047_Supplemental_Files

giaf047_Authors_Response_To_Reviewer_Comments_Original_Submission

giaf047_Authors_Response_To_Reviewer_Comments_Revision_1

giaf047_Authors_Response_To_Reviewer_Comments_Revision_2

giaf047_GIGA-D-24-00350_Original_Submission

giaf047_GIGA-D-24-00350_Revision_1

giaf047_GIGA-D-24-00350_Revision_2

giaf047_GIGA-D-24-00350_Revision_3

giaf047_Reviewer_1_Report_Original_Submission Jorge Duitama -- 9/20/2024

giaf047_Reviewer_1_Report_Revision_1 Jorge Duitama -- 12/30/2024

giaf047_Reviewer_1_Report_Revision_2 Jorge Duitama -- 2/12/2025

giaf047_Reviewer_2_Report_Original_Submission Heena Ambreen -- 9/30/2024

giaf047_Reviewer_2_Report_Revision_1 Heena Ambreen -- 1/12/2025

## Data Availability

Raw sequenced reads have been uploaded to the NCBI Sequence Read Archive under accession numbers SRR24060960 for short WGS reads, SRR24061088 and SRR24061087 for long WGS reads, SRR24085385 for ultra-long WGS reads, SRR24112497 for Hi-C reads, SRR24044811 for fruit RNA-seq reads, SRR24044812 for seed RNA-seq reads, SRR24085891 for leaf RNA-seq reads, SRR24085890 for flower RNA-seq reads in *O. purpureiflora*; SRR25460826 for Hi-C reads of *O. emarginata*; SRR25460825 for Hi-C reads for *O. semicastrata*; SRR29820911–SRR29820936 for resequencing reads of LFS1, SRR29824870–SRR29824895 for resequencing reads of LFS2, SRR29837260–SRR29837285 for resequencing reads of LFS3, SRR29856316–SRR29856341 for resequencing reads of LFS4, SRR29887191–SRR29887216 for resequencing reads of LFS5, SRR29761002–SRR29761004, SRR29761010–SRR29761017, SRR29761028–SRR29761030, SRR29761107, SRR29761108, SRR29761114, SRR29761115, SRR29761118, SRR29761123, SRR29761124, SRR29761126, SRR29761139 for resequencing reads of NKS in *O. purpureiflora*. Assembled genomes are available under accession numbers GCA_040955955.1 for *O. purpureiflora*, GCA_029884595.2 for *O. semicastrata*, and GCA_029884605.2 for *O. emarginata*. Annotations, SNPs, and the other files have been submitted to figshare [122]. All additional supporting data are available in the *GigaScience* repository, GigaDB [123], with separate datasets for *O. purpureiflora* [124], *O. semicastrata* [125], and *O. emarginata* [126].

## Additional Files

**Supplementary Table S1:** Protein sequences of the species used for gene prediction.

**Supplementary Table S2:** Species used for comparative genomics.

**Supplementary Table S3:** Species pairs and their estimated divergence times used for time calibration points to infer time-calibrated phylogeny of *O. purpureiflora*.

**Supplementary Table S4:** nQuire results for the ploidy level assessment in *Ormosia* species.

**Supplementary Table S5:** Repeat contents in *Ormosia* assemblies.

**Supplementary Table S6:** Summary of gene functional annotations of the *O. purpureiflora* assembly performed using different databases.

**Supplementary Table S7:** OMArk gene quality assessment.

**Supplementary Table S8:** Number of the genes matching to representative genes in Fabaceae.

**Supplementary Table S9:** Comparison of gene number in part of the InterPro accesssion among 3 *Ormosia* species.

**Supplementary Table S10:** Summary of gene families.

**Supplementary Table S11:** GO enrichment results for specific gene families in *O. purpureiflora*.

**Supplementary Table S12:** KEGG enrichment results for specific gene families in *O. purpureiflora*.

**Supplementary Table S13:** GO enrichment results for significantly expanded gene families in *O. purpureiflora*.

**Supplementary Table S14:** KEGG enrichment results for significantly expanded gene families in *O. purpureiflora*.

**Supplementary Table S15:** GO enrichment results for siginficantly contracted gene families in *O. purpureiflora*.

**Supplementary Table S16:** KEGG enrichment results for significantly contracted gene families in *O. purpureiflora*.

**Supplementary Table S17:** Number of different gene duplication in *Ormosia*.

**Supplementary Table S18:** GO enrichment results for *O. purpureiflora* WGD genes.

**Supplementary Table S19:** KEGG enrichment results for *O. purpureiflora* WGD genes.

**Supplementary Table S20:** GO enrichment results for tandem-duplicated genes in *O. purpureiflora*.

**Supplementary Table S21:** KEGG enrichment results for tandem-duplicated genes in *O. purpureiflora*.

**Supplementary Table S22:** GO enrichment results for proximal-duplicated genes in *O. purpureiflora*.

**Supplementary Table S23:** KEGG enrichment results for proximal-duplicated genes in *O. purpureiflora*.

**Supplementary Table S24:** Syntenic block analysis results for *Ormosia* species.

**Supplementary Table S25:** Structural variations between the *Ormosia* species.

**Supplementary Table S26:** GO enrichment results for *O. purpureiflora* specific inversion on Chromosome 2.

**Supplementary Table S27:** *R* genes in the species.

**Supplementary Table S28:** Summary of transcription factor genes in *Ormosia* species relative to those in the other species.

**Supplementary Figure S1:** Schematic showing the steps of merging repeats measured in RED and EDTA.

**Supplementary Figure S2:** Chromosome numbers observed in *O. purpureiflora* (scale bar, 10 μm).

**Supplementary Figure S3:** Genome size estimation using GenomeScope.

**Supplementary Figure S4:** Gene and repeat number density (bin size, 100,000 bp) along chromosomes in *Ormosia* species. The smoothing line is added for each density distribution by the geom_smooth() function in the ggplot2 program of the R package. Box sizes correspond to chromosome sizes in the *Ormosia* species.

**Supplementary Figure S5:** Dot plots among 3 *Ormosia* species. The red arrow indicates a specific inversion on Chromosome 2 of *O. purpureiflora*.

**Supplementary Figure S6:** Distribution of *R* genes on each chromosome in the 3 *Ormosia* species.

**Supplementary Figure S7:** Scree plot from PCAdapt.

**Supplementary Figure S8:** Cross-validation error plot of admixture analysis.
