# Supplementary figures and images for: Chromosome-scale assemblies of three Ormosia species: repetitive sequences distribution and structural rearrangement
Source: Gigascience. 2025 May 16;14:giaf047. doi: 10.1093/gigascience/giaf047 (PMC12083454; doi:10.1093/gigascience/giaf047)

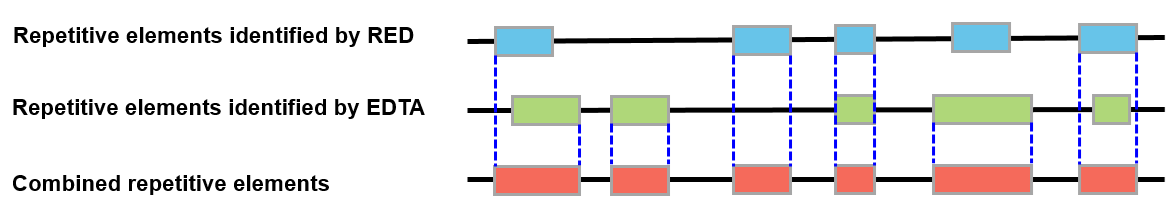

Supplement: giaf047_Supplemental_Files [file giaf047_supplemental_files.zip › Figure_S1.tif]

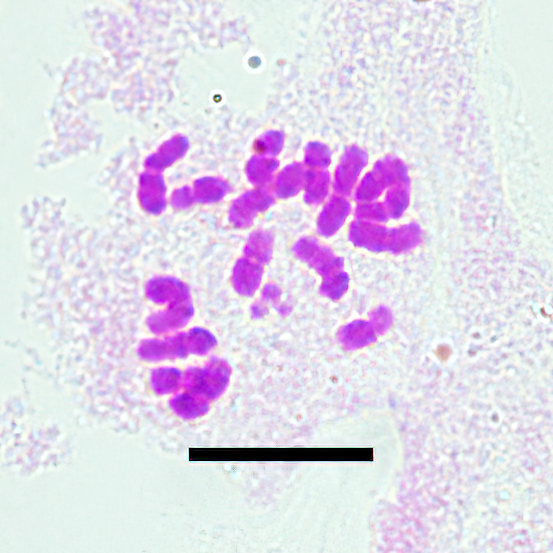

Supplement: giaf047_Supplemental_Files [file giaf047_supplemental_files.zip › Figure_S2.tiff]

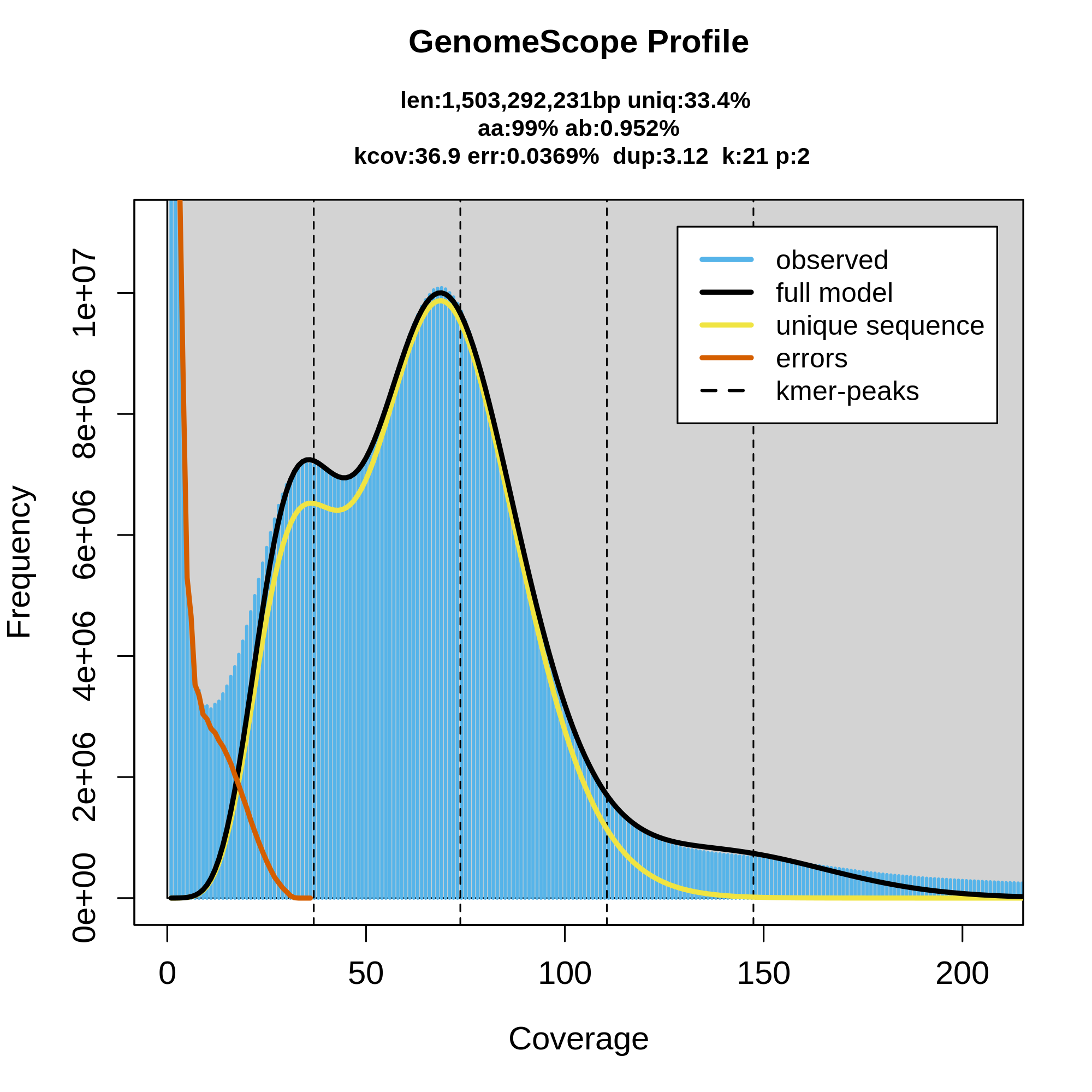

Supplement: giaf047_Supplemental_Files [file giaf047_supplemental_files.zip › Figure_S3.png]

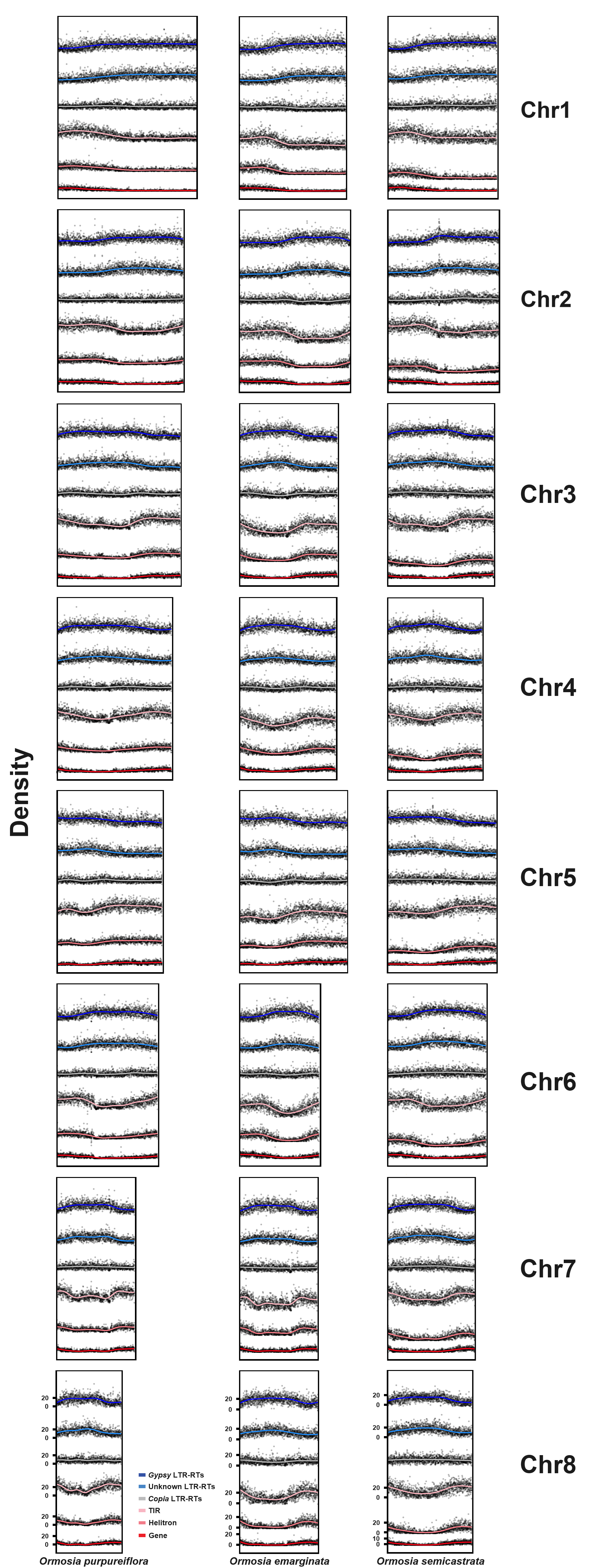

Supplement: giaf047_Supplemental_Files [file giaf047_supplemental_files.zip › Figure_S4.tif]

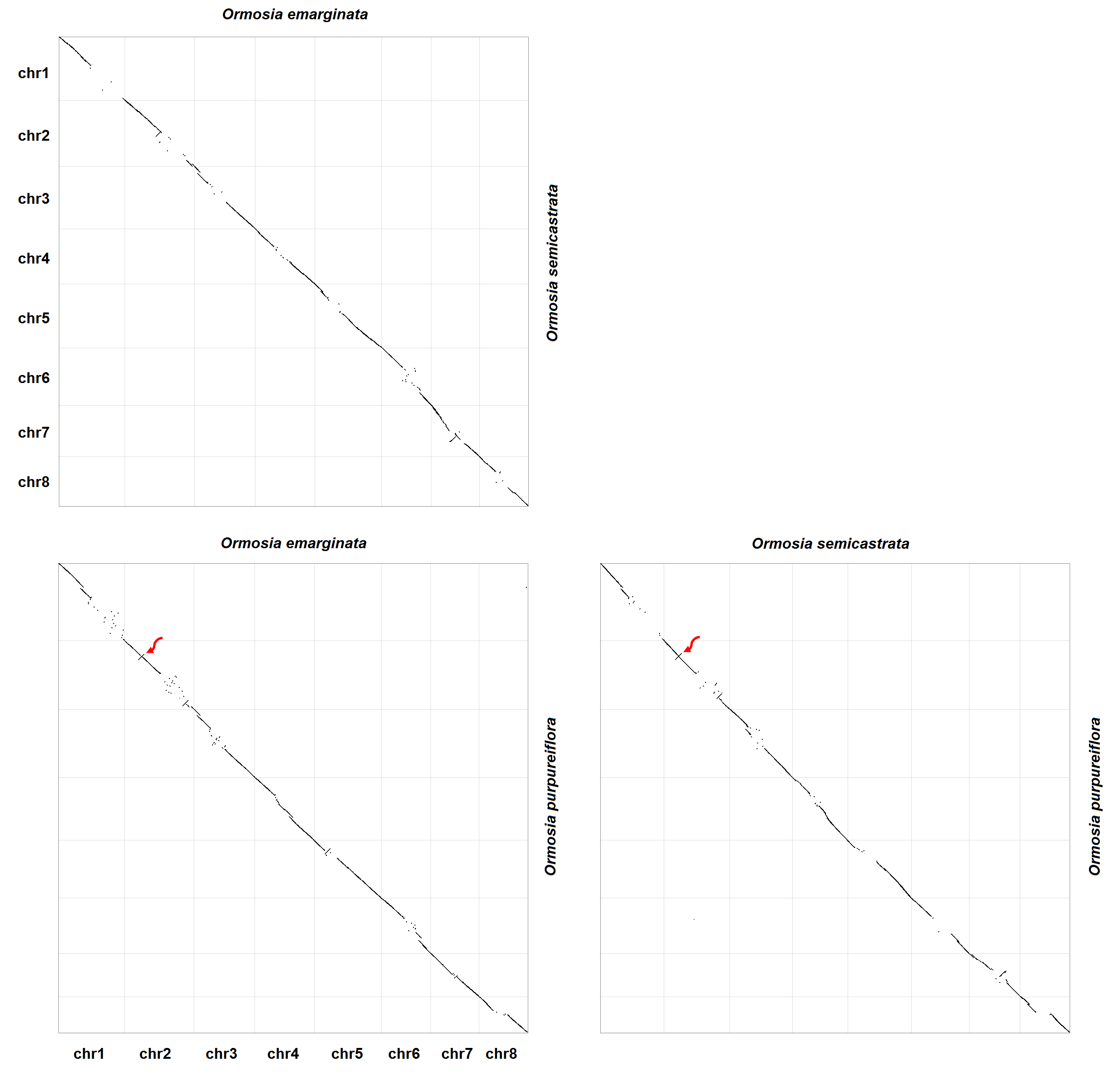

Supplement: giaf047_Supplemental_Files [file giaf047_supplemental_files.zip › Figure_S5.tif]

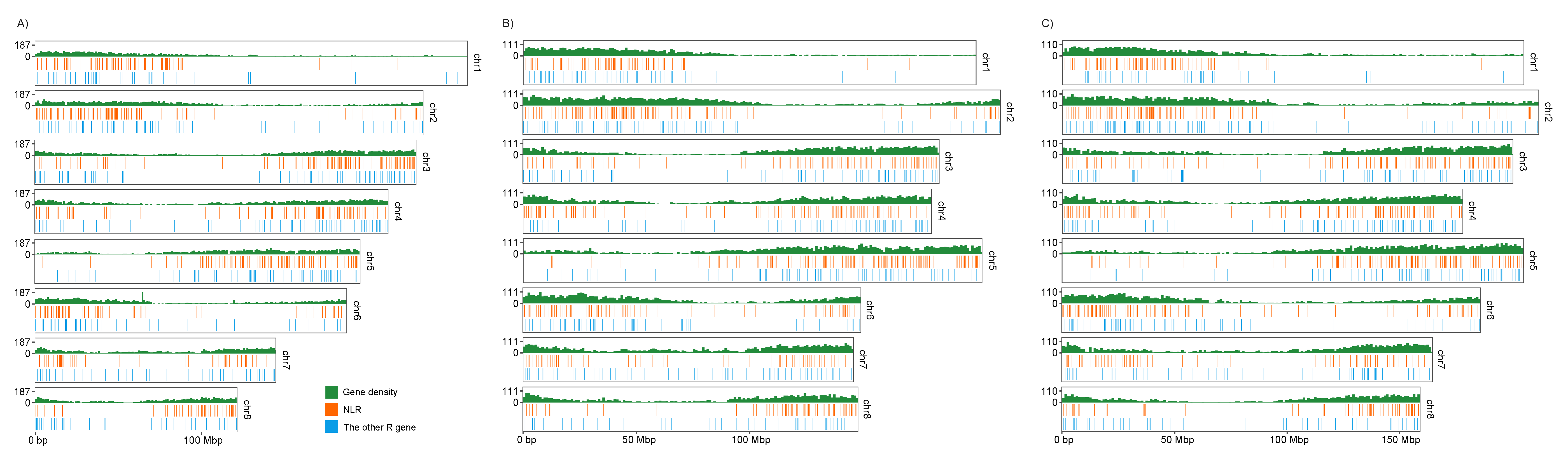

Supplement: giaf047_Supplemental_Files [file giaf047_supplemental_files.zip › Figure_S6.tif]

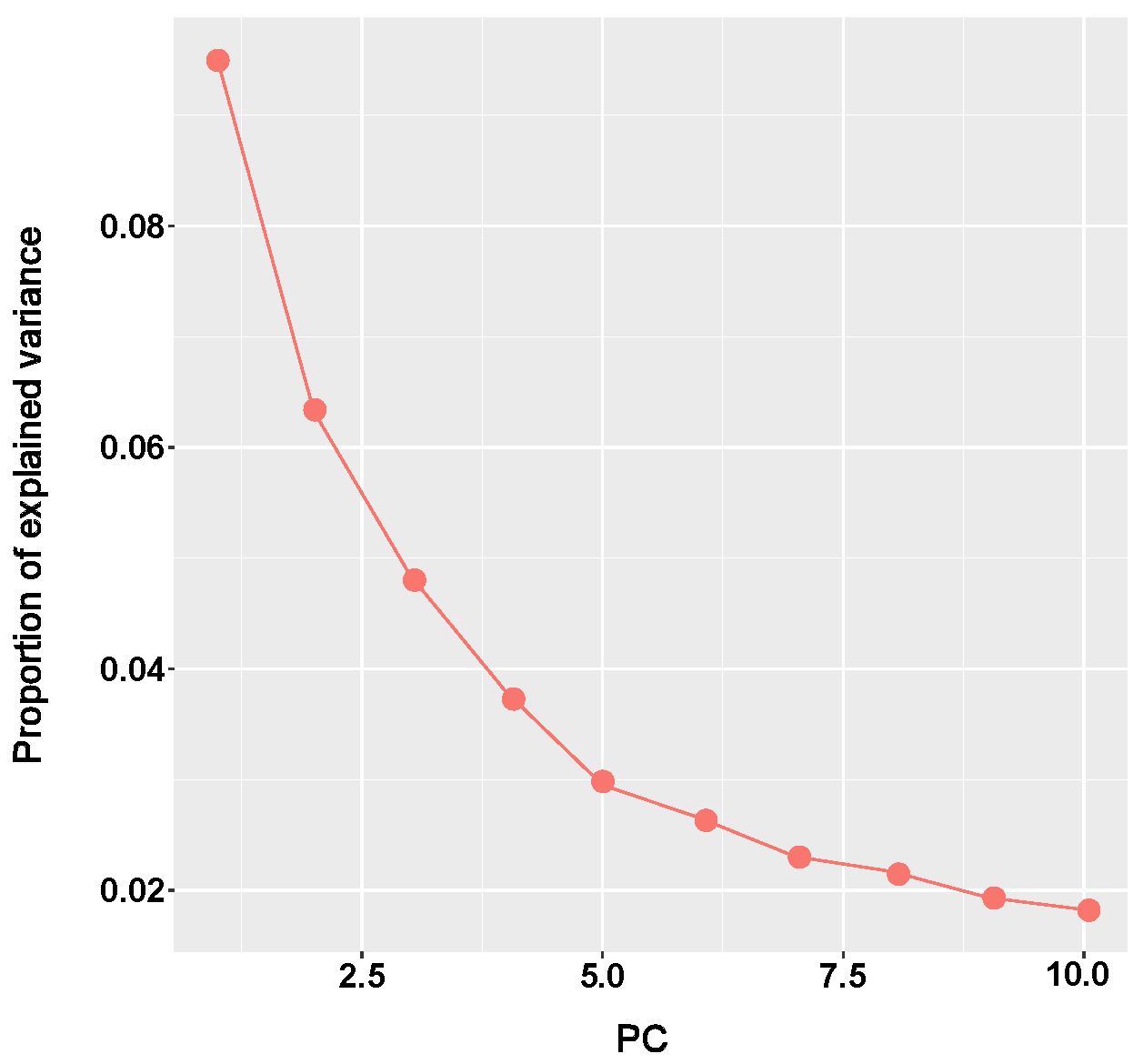

Supplement: giaf047_Supplemental_Files [file giaf047_supplemental_files.zip › Figure_S7.tif]

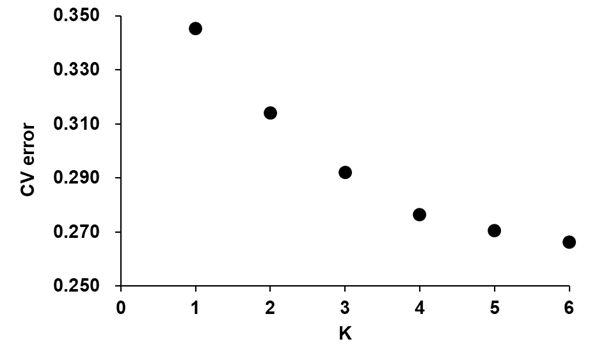

Supplement: giaf047_Supplemental_Files [file giaf047_supplemental_files.zip › Figure_S8.tif]
