# Supplementary material for: Chromosome-scale assemblies of three Ormosia species: repetitive sequences distribution and structural rearrangement
Source: Gigascience. 2025 May 16;14:giaf047. doi: 10.1093/gigascience/giaf047 (PMC12083454; doi:10.1093/gigascience/giaf047)
Supplement: giaf047_GIGA-D-24-00350_Original_Submission [file giaf047_giga-d-24-00350_original_submission.pdf]

GigaScience

Chromosome-scale assemblies of three Ormosia species: Gene-repeat association architecture, structural rearrangement and balancing selection

--Manuscript Draft--

**Manuscript Number:**	GIGA-D-24-00350
**Full Title:**	Chromosome-scale assemblies of three Ormosia species: Gene-repeat association architecture, structural rearrangement and balancing selection
**Article Type:**	Data Note
**Funding Information:**	
**Abstract:**	Background:<br>The genus Ormosia is in Fabaceae family. China is one of its centers with almost all of its species endemic, suggesting need for genomic studies to understand its evolution and help its conservation and usage. Therefore, the chromosome-scale assembly of O. purpureiflora was performed, and that for O. emarginata and O. semicastrata were updated.<br><br>Findings:<br>The genome assembly sizes of the three species varied from 1.42 to 1.58 Gb, and O. purpureiflora was the largest. Repeats accounted for 74.0–76.3% of sequences in the assemblies; the predicted genes varied from 50,517 to 55,061.<br>Repeats contributed largely to the Ormosia chromosome architectures, in which Helitron and Terminal Inverted Repeat (TIR) were associated with gene distribution, while Gypsy and unknown LTR were related to structural rearrangements.<br>Ormosia contained substantial resistance (R) genes but fewer transcription factor genes. Alkaloid-, terpene- and flavonoid-related genes were found with tandem or proximal duplications. Some growth- and defense-related genes were missed in O. purpureiflora.<br>By resequencing 153 genotypes (~30 Gb data each) in 6 O. purpureiflora (sub)populations, 276,854 high-quality single nucleotide polymorphisms (SNPs) were identified, in which 2632 were presumably adaptive SNPs, and their function was mainly related to carbohydrate metabolism. High genetic diversity in O. purpureiflora was revealed even though it had very small populations. A weak and absent spatial genetic structures indicating high pollen flows within/among the (sub)populations. An approximately 30-Mb region on chromosome 5 was found to likely be under balancing selection.<br><br>Conclusions:<br>The Ormosia assemblies provide valuable resources for evolutionary study, conservation and utility in both Ormosia and Fabaceae.
**Corresponding Author:**	Zheng-Feng Wang<br>South China Botanical Garden, Chinese Academy of Sciences<br>CHINA
**Corresponding Author Secondary Information:**	
**Corresponding Author's Institution:**	South China Botanical Garden, Chinese Academy of Sciences
**Corresponding Author's Secondary Institution:**	
**First Author:**	Zheng-Feng Wang
**First Author Secondary Information:**	
**Order of Authors:**	Zheng-Feng Wang
	En-Ping Yu
	Lin Fu
	Hua-Ge Deng

Powered by Editorial Manager® and ProduXion Manager® from Aries Systems Corporation

	Wei-Guang Zhu
	Feng-Xia Xu
	Hong-Lin Cao
**Order of Authors Secondary Information:**	
**Additional Information:**	
**Question**	**Response**
Are you submitting this manuscript to a special series or article collection?	No
**Experimental design and statistics**<br><br>Full details of the experimental design and statistical methods used should be given in the Methods section, as detailed in our Minimum Standards Reporting Checklist. Information essential to interpreting the data presented should be made available in the figure legends.<br><br>Have you included all the information requested in your manuscript?	Yes
**Resources**<br><br>A description of all resources used, including antibodies, cell lines, animals and software tools, with enough information to allow them to be uniquely identified, should be included in the Methods section. Authors are strongly encouraged to cite Research Resource Identifiers (RRIDs) for antibodies, model organisms and tools, where possible.<br><br>Have you included the information requested as detailed in our Minimum Standards Reporting Checklist?	Yes
**Availability of data and materials**<br><br>All datasets and code on which the conclusions of the paper rely must be either included in your submission or deposited in publicly available repositories (where available and ethically	Yes

Powered by Editorial Manager® and ProduXion Manager® from Aries Systems Corporation

appropriate), referencing such data using a unique identifier in the references and in the “Availability of Data and Materials” section of your manuscript.<br><br>Have you have met the above requirement as detailed in our Minimum Standards Reporting Checklist?	

Powered by Editorial Manager® and ProduXion Manager® from Aries Systems Corporation

Manuscript Click here to access/download;Manuscript;final_MS.docx

Date note

Chromosome-scale assemblies of three *Ormosia* species: Gene-repeat association architecture, structural rearrangement and balancing selection

Zheng-Feng Wang[1, 2, 3, 4], En-Ping Yu[1, 2, 3, 4, 5], Lin Fu[1, 3, 4, 6], Hua-Ge Deng[7], Wei-Guang Zhu[1, 2, 3, 4], Feng-Xia Xu[1, 3, 4, 6], Hong-Lin Cao[1, 2, 3, 4]

[1] Guangdong Provincial Key Laboratory of Applied Botany, South China Botanical Garden, Guangzhou, 510650, China

[2] Key Laboratory of Vegetation Restoration and Management of Degraded Ecosystems, South China Botanical Garden, Chinese Academy of Sciences, Guangzhou 510650, China

[3] Key Laboratory of National Forestry and Grassland Administration on Plant Conservation and Utilization in Southern China, South China Botanical Garden, Chinese Academy of Sciences, Guangzhou 510650, China

[4] South China National Botanical Garden, Guangzhou 510650, China

[5] University of Chinese Academy of Sciences, Beijing 100049, China

[6] Key Laboratory of Plant Resources Conservation and Sustainable Utilization, South China Botanical Garden, Chinese Academy of Sciences, Guangzhou 510650, China

[7] Management Office of Guangdong Luofushan Provincial Nature Reserve, Huizhou, 516133, China

Joint first authors:

Zheng-Feng Wang, En-Ping Yu, Lin Fu

Corresponding authors:

Zheng-Feng Wang (wzf@scib.ac.cn); Hong-Lin Cao (caohl@scib.ac.cn)

Abstract

Background: The genus *Ormosia* is in Fabaceae family. China is one of its centers with almost all of its species endemic, suggesting need for genomic studies to understand its evolution and help its conservation and usage. Therefore, the chromosome-scale assembly of *O. purpureiflora* was performed, and that for *O. emarginata* and *O. semicastrata* were updated.

Findings: The genome assembly sizes of the three species varied from 1.42 to 1.58 Gb, and *O. purpureiflora* was the largest. Repeats accounted for 74.0–76.3% of sequences in the assemblies; the predicted genes varied from 50,517 to 55,061.

Repeats contributed largely to the *Ormosia* chromosome architectures, in which Helitron and Terminal Inverted Repeat (TIR) were associated with gene distribution, while *Gypsy* and unknown LTR were related to structural rearrangements.

Ormosia contained substantial resistance (*R*) genes but fewer transcription factor genes. Alkaloid-, terpene- and flavonoid-related genes were found with tandem or proximal duplications. Some growth- and defense-related genes were missed in *O. purpureiflora*.

By resequencing 153 genotypes (~30 Gb data each) in 6 *O. purpureiflora* (sub)populations, 276,854 high-quality single nucleotide polymorphisms (SNPs) were identified, in which 2632 were presumably adaptive SNPs, and their function was mainly related to carbohydrate metabolism. High genetic diversity in *O. purpureiflora* was revealed even though it had very small populations. A weak and absent spatial genetic structures indicating high pollen flows within/among the (sub)populations. An approximately 30-Mb region on chromosome 5 was found to likely be under balancing selection.

Conclusions: The *Ormosia* assemblies provide valuable resources for evolutionary study, conservation and utility in both *Ormosia* and Fabaceae.

Keywords: Adaptive loci; Comparative genomics; Gene families; Gene duplication; Genetic diversity; Population genetics; RNA-seq; Repeat-mediated chromosome architectures; SNP calling; Structure variation

Data Description

Context

The genus *Ormosia* Jackson comprises ca. 130–150 species in the Fabaceae family [1, 2]. They are trees and shrubs that prefer a warm climate. According to fossil records, they were distributed in northern regions in the north hemisphere and migrated southwards during the Paleogene or Neogene due to climate cooling [3]. Their current distributions include tropical America, southeast Asia and north Australia [1–3], displaying a typical Asian–American Tropical Disjunction Pattern [1], and continental Asia is assumed to be the center of origin.

The most distinctive characteristics of *Ormosia* are their brightly colored seeds that are red, orange, bicolored red/orange, and black (Fig. 1A), which are often used in ethnic jewelry and other decorative purposes [1]. Some *Ormosia* species have a high timber value, and some are used as landscape trees [4, 5]. In addition, extracts from their seeds, roots, stems, bark and leaves can be used as medicines [6, 7]. The substances in the extracts are mainly alkaloids, flavonoids, isoflavones, terpenes and lignan [6–8]. Metabolome and transcriptome analyses have indicated that transcription factors play an important role in the regulation of flavonoid/terpenoid biosynthesis in *Ormosia* species [9, 10].

In China, there are about 37 species, 34 of which are endemic [2]. *Ormosia purpureiflora* is such an endemic one. Unlike most *Ormosia* with white or yellow flowers, the flower color of *O. purpureiflora* is purple (Fig. 1B), as shown in its species name (*purpureiflora*). The species has only been found at two sites in southeast China (Fig. 2): Guangdong Longmen Nankunshan (NKS) Provincial Natural Reserve and Guangdong Luofushan (LFS) Provincial Natural Reserve. Field investigations have indicated that there are only approximately 2000 individuals at the two sites (E-P Yu, personal observation). It also suffers from severe pest/disease attacks on both flowers and fruit (Fig. 1C–E), causing a low seed yield in the field.

With the development of high-throughput sequencing technologies, full genome information has been performed using genome assembly in diversified species, enabling their conservation, restoration, management and utilization. Therefore, our work aimed to provide a high-quality genome of *O. purpureiflora* using long- and short-read whole genome sequencing (WGS), high-throughput chromosome conformation capture (Hi-C) sequencing, and annotation with RNA

sequencing (RNA-Seq) of different tissues. Based on the genome, we also detected genetic diversity and performed population genetics in *O. purpureiflora* by resequencing 153 samples from two sites.

For comparative genomics, we also updated the assemblies of previously published *O. emarginata* and *O. semicastrata* genomes [11] with Hi-C data to obtain their chromosome-scale genome assemblies. Compared to *O. purpureiflora*, both *O. emarginata* and *O. semicastrata* are widespread species in southern and southeast China, but *O. emarginata* extends to Vietnam. Both *O. emarginata* and *O. semicastrata* are large trees, while *O. purpureiflora* is a small shrub (Fig. 1). Phylogenetic studies by Torke et al. [1] showed that *O. emarginata* and *O. semicastrata* were in different clades, with *O. emarginata* in Old World *Ormosia* clade 1 and *O. semicastrata* in Old World *Ormosia* clade 2, while *O. purpureiflora* was not included in their phylogenies.

Methods

Chromosome number observation

The individual used for chromosome number observation in *O. purpureiflora* was regenerated from seeds collected from LFS. Its root tips were pretreated with 0.002 M 8-hydroxyquinoline for 6 h at room temperature, fixed in 3 : 1 (v:v) absolute ethanol:glacial acetic acid for 24 h, transferred to 70% ethanol and stored at −4°C. For chromosome counts, fixed root tips were hydrolyzed in 1 : 1 (v:v) 1M absolute ethanol:hydrochloric acid at room temperature for 7 min, rinsed in water and stained with carbol fuchsin for 4 min. Meristems were subsequently excised and squashed for microscope observation. Photographs were taken using an Olympus BX-43 microscope at 100x magnification with an Olympus DP26 camera.

Sample collection and sequencing

One *O. purpureiflora* individual (Fig. 1F) used for genome assembly was collected from LFS. For genome assembly, genomic DNA was isolated from its leaf tissues and multiple libraries, including long- and short-read WGS and Hi-C libraries, were constructed. For gene annotation, RNA was isolated from its leaves, flowers, seeds and fruit, and RNA-seq libraries from different tissues were then constructed. Long-read WGS was applied using an Oxford Nanopore Technologies (ONT) PromethION sequencer. Under the ONT platform, both long and ultra-long (50 kb)

sequencing libraries were generated. Short-read WGS, Hi-C and RNA-seq were performed using an MGI DNBSEQ-T7 sequencer with a 150-bp paired-end (insert size 300 bp) sequencing strategy.

To perform population genetic studies on *O. purpureiflora*, leaf samples from 153 individuals were collected from 6 (sub)populations from LFS and NKS (Table 1, Fig. 2A). Their positions were recorded using handheld GPS. Leaves collected from each individual were immediately placed into a sealed plastic bag with silica gel. Resequencing was then performed using a MGI DNBSEQ-T7 sequencer with PE-150bp model. Each sample yielded about 30 Gb of data.

Leaf and flower RNA-seq libraries were constructed and sequenced by Annoroad Gene Technology (AGT, Beijing, China), and the remaining including 153 resequencing samples were evaluated by GrandOmics Biosciences (GB, Wuhan, China).

For *O. emarginata* and *O. semicastrata*, their Hi-C libraries were constructed using leaf samples from the same individuals used for primary genome assembly [11], and then sequencing was performed by GrandOmics Biosciences.

Date pre-processing

Short WGS reads of *O. purpureiflora* and Hi-C reads of all three *Ormosia* species were quality trimmed using Sickle v1.33 (RRID:SCR_006800) [12] by removing the reads with base quality values less than 30 and lengths shorter than 80 bp. The WGS reads of *O. purpureiflora* were further error corrected using RECKONER v1.1 [13]. Using the error-corrected reads of *O. purpureiflora*, the 21-mer frequencies were generated using Jellyfish 2.3.0 (RRID:SCR_005491) [14], and its results were inputted into GenomeScope 2.0 (RRID:SCR_017014) [15] for *O. purpureiflora* genome size estimation. The adapters of ONT long WGS reads of *O. purpureiflora* were removed using Porchop 0.2.4 [16]. ONT reads larger than 20 kb were further extracted from full ONT reads and defined as a 20 kb ONT read set, which was used for *O. purpureiflora* genome assembly.

Genome assembly

Using a 20 kb ONT read set, *O. purpureiflora* genomes were assembled using nextDenovo 2.3.1 [17]. After assembly, Pseudohaploid [18] and Purge_Dups v1.2.6 (RRID:SCR_021173) [19] were applied to examine and remove duplications caused by heterozygosity in the assemblies. The

assembles were then polished by racon v1.5.0 (RRID:SCR_017642) [20] (run twice), hapo-G v1.3.2 [21] (run twice) and polypolish v0.5.0 [22], and possible misassembly was further corrected by Depthcharge v0.2.0 [23]. Contigs with a length smaller than 1000 bp were subsequently removed. The corrected assembly was scaffolded by Hi-C reads using Scaffhic v1.1 [24], Juicer pipeline 1.6 (RRID:SCR_017226) [25] and 3d-dna 201008 (RRID:SCR_017227) [26] and gap closed using TGS-GapCloser v1.2.1 (RRID:SCR_017633) [27]. The gap-closed assembly was further polished by racon, hapo-G and polypolish. After Hi-C scaffolding and gap closing, we ran Redundans 0.14a [28] to remove redundant sequences not unanchored to the chromosomes. The assembly was then uploaded to GenBank to check for possible contamination. The sequences that gave hits to bacterial/fungal sequences were removed from the assembly. Subsequently, the telomeric repeats at each chromosome end were identified (with the parameter of "--motifs TTTAGGG –matchAny") and recovered by Teloclip v0.0.3 [29]. The assembly was then polished by racon, hapo-G and polypolish to obtain complete assembly. To evaluate the completeness of the assembly, Benchmarking Universal Single-Copy Orthologs (BUSCO) v5.5.0 (RRID:SCR_015008)) [30] with the eudicots_odb10.2020-09-10 database was used. The database contains 2326 conserved core genes of eudicots.

For *O. emarginata* and *O. semicastrata*, using their primary assembly [11], their chromosome-scale assembly followed the above *O. purpureiflora* genome assembly procedures from the step using Hi-C reads.

Repeat and gene prediction

The repeat sequences in three *Ormosia* chromosome-scale assemblies were estimated using both EDTA v2.1.0 (RRID:SCR_022063) [31] and RED v2.0 [32]. The repeat results of the two programs in each assembly were subsequently combined and used to soft-mask its corresponding assembly with bedtools v2.29.2 (RRID:SCR_006646) [33].

The soft-masked *Ormosia* assemblies were annotated using BRAKER2 v.2.0 [34] and Funannotate pipeline v1.8.16 [35]. BRAKER2 incorporated both RNA-seq reads and reference proteins of eight species (Table S1) for transcriptome- and homology-based annotation, except for *ab initio*-based gene prediction. Funannotate was used to integrate the BRAKER2 results to obtain

consensus gene sets. Gene prediction in Funannotate included "train", "predict" and "update" steps. In the latter two steps, the following parameters were used: "-max_intronlen 100,000 -busco_db embryophyta -organism other". For predicted genes in *Ormosia* species, function annotations were performed using Funannotate under the command "annotate". The annotation databases were dbCAN v10.0 (RRID:SCR_013208) [36], EggNOG v5.0.2 (RRID:SCR_002456) [37], Gene Ontology (GO, RRID:SCR_002811) [38, 39], Kyoto Encyclopedia of Genes and Genomes (KEGG, RRID:SCR_012773) [40], InterPro v5.62-94.0 (RRID:SCR_006695) [41], MEROPS v12.0 [42] (RRID:SCR_007777), Pfam v35.0 [43] (RRID:SCR_004726), SignalP 5.0b (RRID:SCR_015644) [44] and UniProt v2023_02 (RRID:SCR_002380) [45]. Protein sequences were also annotated with the online tool KAAS [46].

For the following comparative genomic analyses, only the longest transcript for each gene in all species was used unless mentioned otherwise. In addition, for genome comparison, the protein-coding genes of all other species used in our phylogenetic analysis (see below) were also functionally annotated using the same procedures used for *Ormosia* species.

Gene family and comparative genomics

Orthologous groups (gene families) in *Ormosia* were identified by OrthoFinder 3.0.0 (RRID:SCR_017118) [47, 48], using the protein-coding gene sequences of the other 17 species (Table S2) as inputs. After orthologous group inferences, phylogenetic analysis using 1131 single-copy orthologs was performed with STAG [49] and STRIDE [50], which were included in OrthoFinder. The gene family file produced by Orthofinder was further used to analyze gene family expansion or contraction with CAFE v5 (RRID:SCR_018924) [51]. The species tree with divergence time used in CAFE was obtained using MCMCTree [52], in which 12 calibration time points from the TimeTree database (http://timetree.org/, Table S3) were used to calibrate the species tree. After CAFE analysis, for the significantly expanded and contracted gene families in *O. purpureiflora*, GO and KEGG enrichment analyses were conducted using TBtools v2.030 [53].

Gene duplications, synteny and structural variations analysis

The identification of ancient whole genome duplication (WGD) events in *Ormosia* and their

sister species *Lupinus albus* (see results) was carried out using wgd v1.1.2 [54]. Gene duplication in *Ormosia* was examined by Doubletrouble v0.99.1 [55], which classified the duplication origin into WGD, tandem duplications (TD), proximal duplications (PD), transposed duplications (TRD) and dispersed duplications (DD) [56]. *Lupinus albus* was used as an outgroup species in the analysis. For WGD, TD and PD genes in *O. purpureiflora*, GO and KEGG enrichment analysis were performed using TBtools.

Syntenic regions within and between *Ormosia* and *L. albus* genome assemblies were identified with MCScanX [57] and visualized in Shinycircos [58] or SynVisio [59]. The "-s 30" (MATCH_SIZE) parameter of synteny analysis in MCScanX was used. The structural variations were identified using chromeister v1.5.a [60] and plotsr v1.1.0 [61].

RNA-seq analysis of tissues

The assembled *O. purpureiflora* genome and its predicted protein-coding genes were used for RNA-seq analyses for four tissues (leaf, flower, fruit and seed) with nf-core/rnaseq 3.14.0 [62]. Differential gene expression among tissues was estimated with nf-core/differentialabundance 1.4.0 [63].

Nucleotide binding leucine-rich repeats (NLR) and the other resistance (*R*) gene identification

NLR genes are the main plant *R* genes against viruses, bacteria, nematodes, fungi, oomycetes and insects [64, 65]. These genes consist of three canonical domains: a variable N-terminal domain, a central nucleotide-binding domain (NB-ARC) and a C-terminal domain comprising leucine-rich repeat (LRR) [66]. At the N terminus, three types were identified, namely Toll/interleukin-1 receptor (TIR), coiled-coil (CC) and resistance to powdery mildew8 (RPW8) [67]. The InterPro/Pfam entries for these genes were NB-ARC (IPR002182/PF00931), TIR (IPR000157/PF01582/PF13676), CC (IPR038005), RPW8 (IPR008808/PF05659) and LRR (IPR001611/PF00560/, IPR013101/PF07723, IPR011713/PF07725, IPR025875/PF12799, IPR026906/PF13306, IPR001611/PF13516/PF13855, PF14580 and IPR032675). Except for NLR genes, the other *R* genes were identified according to De-la-Cruz et al. [68] using their InterPro entries.

Transcription factor

Transcription factor (TF) genes in *Ormisa* and the other genomes were identified by TF prediction online tools (https://planttfdb.gao-lab.org/prediction.php) [69].

SNP calling and genetic structure

Single nucleotide polymorphisms (SNPs) in 153 *O. purpureiflora* individuals were called by modified dDocent v2.7.6 [70, 71], using the *O. purpureiflora* genome assembled in this study as a reference. After SNP calling, indels and SNPs that showed low quality and deviated from Hardy–Weinberg equilibrium (HWE) and linkage disequilibrium (LD) were removed as described in the "SNP Filtering Tutorial" of dDocent (http://www.ddocent.com/filtering/) and by Liu et al. [71]. Finally, we retained only the biallelic SNPs and those that were not missing across all genotyped individuals with vcftools v0.1.16 (RRID:SCR_001235) [72].

Before population genetic structure inferences, PCAdapt v4.3.5 [73, 74] and BayPass v2.4 [75] were applied to detect SNPs under selection (i.e., adaptive SNPs). PCAdapt first performed a principal component analysis (PCA) and then applied a Scree plot to help identify suitable principal components that were used for regression with each SNP. After regression analysis, a *q*-value (i.e., adjusted *P*) < 0.01 was implemented as the threshold for determining outliers (SNPs probably under selection). For BayPass, a core model with the default parameters was used. It estimated the F_{ST} alike XtX statistic to account for the variance–covariance structure. To determine the significance in the XtX statistic, a calibrated threshold (99%) calculated by simulating pseudo-observed datasets (100,000 SNPs) was provided. The SNPs that fell within the 99.9% quantile of the pseudo-observed XTX distribution were considered candidates putatively under selection.

Adaptive SNPs were determined by those occurring in both PCAdapt and BayPass results and subsequently removed from the whole set of above filtered SNPs. Therefore, only "neutral" SNPs were used for genetic structure and genetic diversity inferences, which were performed via PCA and ADMIXTURE (RRID:SCR_001263) [76]. PCA analysis was performed using SNPRelate v1.36.0 [77], and ADMIXTURE was performed via the AdmixPipe v3.2 pipeline [78]. In AdmixPipe, the possible genetic group (*K*) was estimated from 1 to 6 and the number of replicates for each *K* value was set as 20. After AdmixPipe was run, the best *K* value was determined with cross-validation (CV)

errors. Then, for the inferred *K*, CLUMPAK v1.1 [79] was applied to estimate the mean membership coefficient of 20 replicates per individual.

Genetic diversity

Genetic diversity parameters, namely observed heterozygosity (*Ho*), expected heterozygosity (*He*) and inbreeding coefficient (*Fis*), were estimated by vcftools, and the nucleotide diversity within (π) and between (*dxy*) populations and pairwise genetic differentiation (*Fst*) were calculated using pixy v1.2.7.beta1 [80].

Genetic relatedness and fine-scale spatial genetic structure (SGS)

The pairwise relatedness coefficients of Lynch and Ritland [81] were calculated among individuals in each (sub)population using PopCluster v1.3.1.0 [82]. Fine-scale SGS was then analyzed by regressing pairwise relatedness coefficients on geographical distance using GenAlEx 6.51b2 [83].

Results

Genome sequencing

For *O. purpureiflora*, the ONT sequencing platform generated approximately 181.6 Gb WGS reads, including 51.3 Gb ultra-long reads. The short sequencing platform generated approximately 139.3 Gb WGS reads and 146.8 Gb Hi-C reads. RNA-seq was approximately 20.4, 21.9, 23.3 and 25.3 Gb for leaf, flower, fruit and seed samples, respectively. For *O. emarginata* and *O. semicastrata*, 148.7 and 123.6 Gb Hi-C reads, respectively, were generated.

Genome assembly

For *O. purpureiflora*, the genome size estimated by GenomeScope was 1,503,292,231 bp, with 66.6% as repeats and 0.952% heterozygosity (Fig. S1). The initial genome assembly size was 1,811,176,403 bp, with 313 contigs and an N50 of 50,908,349 bp. After redundancy removal, Hi-C scaffolding and gap closing, the final assembly was 1,584,128,722 bp in length with 1,583,483,254 bp (99.96%) anchored within 8 chromosomes (Table 2, Fig. 3A), which was consistent with

chromosome number observation (Fig. 1A). The longest chromosome was 259,935,025 bp long, and the shortest was 121,398,155 bp.

The assembly sizes of *O. emarginata* and *O. semicastrata* were 1,420,253,666 and 1,510,687,319 bp, respectively (Table 1). They each obtained 8 chromosome-level scaffolds, accounting for 99.99 and 99.97% of their assemblies.

For *O. purpureiflora*, BUSCO evaluation revealed 98.3% complete BUSCOs (including 89.4% complete and single-copy, and 8.9% complete but duplicated), whereas 0.3% fragmented and 1.4% missing BUSCOs. For *O. emarginata*, the complete BUSCO score was 97.0% (including 89.4% complete and single-copy, and 7.6% complete but duplicated) with 0.5% fragmented and 2.5% missing; for *O. semicastrata*, the complete BUSCO score was 98.4% (including 90.4% complete and single-copy, and 8.0% complete but duplicated) with 0.1% fragmented and 1.5% missing.

Repeat and gene annotation

RED analyses revealed 1,037,006,095 (65.5%), 885,912,252 (62.4%) and 968,176,023 bp (64.1%) repetitive sequences in *O. purpureiflora*, *O. emarginata* and *O. semicastrata*, respectively. EDTA analyses revealed 1,139,417,595 (71.9%), 989,514,254 (69.6%) and 1,074,353,470 bp (71.1%) repetitive sequences in *O. purpureiflora*, *O. emarginata* and *O. semicastrata*, respectively (Table S4). After combining RED and EDTA results, 1,209,324,791 (76.3%), 1,051,218,280 (74.0%) and 1,135,447,010 bp (75.2%) repetitive components were identified in *O. purpureiflora*, *O. emarginata* and *O. semicastrata*, respectively. According to EDTA, the *Gypsy*-like long terminal repeat retrotransposon (LTR-RT) family was the most abundant repetitive sequence, accounting for 33.51, 35.45 and 27.73% of the *O. purpureiflora*, *O. emarginata* and *O. semicastrata* assemblies, respectively.

Gene prediction resulted in 55,061 genes coding for 59,809 proteins in *O. purpureiflora*. In *O. emarginata* and *O. semicastrata*, the predictions resulted in 50,517 and 51,220 genes encoding 54,456 and 55,363 proteins, respectively. Approximately 70.8, 71.9 and 72.4% of the protein-coding genes were functionally annotated on at least 1 database in *O. purpureiflora*, *O. emarginata* and *O. semicastrata*, respectively (Table S5).

Gene density distribution in *Ormosia* showed that they were associated with repeat density

distribution on each chromosome (Fig. 3B), indicating possible repeat-mediated gene formation. In chromosomes 3, 4, 6, 7 and 8, the low repeat and gene density generally appeared in the middle of the chromosome, while for chromosomes 1, 2 and 5, their densities were generally high on one arm side but low on the other side. Compared to their sister species *L. albus* [84], no such gene-repeat density correlation was observed, indicating that the correlations could be *Ormosia* specific. Close examination of different repeat types indicated that Helitron and Terminal Inverted Repeats (TIRs) were associated with gene distribution in the *Ormosia* assemblies (Fig. 4 and Fig. S2).

After retrieving both up- and downstream 1000-bp sequences in Helitron/TIR repeats in *Ormosia purpureiflora*, there were 21,641/9,363 genes in these sequences. By also removing possible LTR-mediated genes and reciprocally excluding Helitron- or TIR-mediated genes, we finally retained 7216 possible Helitron-mediated genes and 2728 possible TIR-mediated genes. Enrichment analysis indicated that Helitron-mediated genes were mainly related to C-terminal protein amino acid modification and defense response to bacterium in GO's biological process (BP) category (Table S6) and anthocyanin and carotenoid biosynthesis in KEGG (Table S7). TIR-mediated genes were mainly related to xyloglucan metabolic process and disaccharide biosynthetic process in GO's BP category (Table S8), and carbon fixation in photosynthetic organisms and plant hormone signal transduction in KEGG (Table S9). However, these enrichment analyses were not significant after p value correction, suggesting that the genes mediated by these two transposable elements were diverse.

Repeat sequences are an important driver for gene formation and species evolution. In maize (*Zea mays* ssp. *mays*), Lai et al. [85] found that the locations of transposable elements (DNA TEs and *Copia*-superfamily retrotransposons) were correlated with gene density distribution, influencing gene regulation and conveying adaption to a new environment. Further exploitation of the correlation between the genes and repeats was required to delineate its evolutionary adaption in *Ormosia.*

According to InterPro function annotation, we found that some photosynthesis-related genes were not annotated in *O. purpureiflora* by comparison to *O. emarginata* and *O. semicastrata* (Table S10). In addition, the number of genes with accession of IPR001280 (Photosystem I PsaA/PsaB) in *O. purpureiflora* was 3, which was lower than that in *O. emarginata* (8) and *O. semicastrata* (9).

Moreover, the genes related to plant–pathogen interaction (EDS1-like, IPR044214), plant reproduction (DBP10, C-terminal, IPR012541), pyrimidine/nucleotide metabolism (deoxyuridine triphosphate nucleotidohydrolase, IPR008181; dUTPase-like, IPR029054/IPR036157), regeneration (Thioredoxin DCC1, IPR044691), SEED MATURATION PROTEIN1 (SMP1, IPR044984) and nodulin (IPR003387) are missing (Table S10).

In the plant–pathogen interaction pathway, EDS1 is responsible for defense amplification and programmed cell death (Fig. S3). DBP10-type genes are related to maturation of both male and female gametophytes in *Arabidopsis thaliana* [86]. dUTPase is an enzyme that hydrolyzes dUTP to dUMP and pyrophosphate. This reaction can control the dUTP concentration and provide a precursor (dUMP) for the thymine nucleotide synthesis. Excess dUTP causes extensive excision repair, leading to DNA breakage and cell death [87, 88]. In *A. thaliana*, thioredoxin DCC1 influences shoot regeneration. This gene loss inhibits shoot regeneration [89]. SMP1 protects seeds under heat stress [90]. Nodulins are involved in the nodulation of legume roots after rhizobium infection. They take part in the transport of various substances (such as nutrients, amino acids and hormones) for plant development [91]. These missing genes might be related to some mal-adaptation in the nature of *O. purpureiflora*, such as low growth rate, high death rate and disease susceptibility (E-P Yu, personal observation).

Gene family

A total of 47,608 gene families were identified by Orthofinder. In *O. purpureiflora*, 50,275 genes (91.3%) were assigned to 27,347 gene families. Among these families, 454 were *O. purpureiflora* specific (Table S11), and the genes in these families were mainly enriched in endoplasmic reticulum to Golgi vesicle-mediated transport and non-membrane-bounded organelle assembly in GO's BP category (Table S12) and ribosome biogenesis in eukaryotes in KEGG (Table S13).

The phylogenetic tree (Fig. 3C) showed that *O. purpureiflora* was sister to *O. emarginata* and that *Ormosia* was sister to *L. albus*. The estimated divergence time between *O. purpureiflora* and *O. emarginata* was about 2.94 million years ago (95% CI: 1.19–5.00), while the divergence time between *Ormosia* and *Lupinus* was 45.90 million years ago (95% CI: 32.66, 56.44). In *O.*

purpureiflora, 1020 families were expanded and 623 families were contracted. Among these gene families, 205 were significantly ($P<0.05$) expanded, and 84 were significantly contracted. Significantly expanded gene families were mainly enriched in DNA integration and regulation of amino acid transmembrane transport in GO's BP category (Table S14) and alkaloids, polyketide and zeatin biosynthesis in KEGG (Table S15). The significantly contracted gene families were mainly enriched in transcription by lipid transport and lipid localization in GO's BP category (Table S16) and terpenoid biosynthesis in KEGG (Table S17).

For the genes in the contracted gene families in terpenoid biosynthase, they mainly belonged to Cytochrome P450 (CYP450) genes. CYP450s carry out downstream activity for the final terpenoid products [92, 93]. However, terpenoids were mainly represented by 2 conserved domains with Pfam ID PF01397 and PF03936 [71]. The comparison indicated that *O. purpureiflora* assembly annotated 23 and 25 of these genes, which were slightly lower than those in *O. emarginata* (31 and 26) and *O. semicastrata* (26 and 28). Nevertheless, the number of genes in *Ormosia* was much higher than that in their sister species, *L. albus* (8 and 10).

Gene duplications, synteny and structural variations analysis

WGD analysis indicated that *O. purpureiflora* underwent one recent ancient WGD event (Fig. 3D), which was shared with the other two *Ormosia* species and *L. albus*.

Gene duplication analysis indicated that the three *Ormosia* species generally displayed similar gene numbers in different duplication types (Table S18). In *O. purpureiflora*, enrichment analysis indicated WGD duplicated genes were mainly related to calcium ion, blue light, flower and development, and cytokinin biosynthetic process in GO's BP category (Table S19) and signaling proteins, glycosylphosphatidylinositol (GPI)-anchored proteins, GTP-binding proteins and SNARE interactions in vesicular transport in KEGG (Table S20). The TD-type genes were mainly related to phloem development, glutathione metabolic process and monoterpenoid/anthocyanin/zeatin biosynthesis/flavonoid biosynthesis (Table S21 and S22). The PD-type genes were mainly related to diterpenoid and triterpenoid biosynthetic process, arginine biosynthetic process, phloem development, and flavone and flavone biosynthesis (Table S23 and S24). These results generally agree with the previous study on *O. emarginata* and *O. semicastrata* studies by Liu et al. [11], which

showed that TD and PD genes were relevant to various (secondary) biosynthetic/metabolic processes, including alkaloid/flavonoid/terpenoid biosynthesis.

Within *Ormosia*, synteny analysis revealed 48, 42 and 45 syntenic blocks in *O. purpureiflora*, *O. emarginata* and *O. semicastrata*, respectively (Table S25). The longest syntenic blocks detected in these *Ormosia* species were between chr2 and chr3, with a size of 39,614,256 bp containing 427 gene pairs in *O. purpureiflora*, 33,895,706 bp containing 383 gene pairs in *O. emarginata*, and 36,266,649 bp containing 424 gene pairs in *O. semicastrata*. A syntenic relationship was illustrated in the CIRCOS plot (Fig. 3B).

Overall, *O. purpureiflora* exhibited highly syntenic relationships with the other two *Ormosia* genomes by both synteny analysis (Fig. 3E) and dot plots (Fig. S4). However, further genetic variation analysis revealed extensive intra-chromosomal rearrangement among *Ormosia* species (Fig. 3F). Clearly, these arrangements generally happened in specific hot chromosomal regions, where the gene density was low, indicating unstable genome architecture in these hot regions but conserved genome architecture in gene-rich regions in *Ormosia*.

Particularly, *O. semicastrata* showed more divergence from *O. purpureiflora* and *O. emarginata*, i.e., high unaligned proportion (52.36% unaligned with *O. purpureiflora* assembly, 50.54% unaligned with *O. emarginata* assembly, while only 24.88/28.51% unaligned with *O. emarginata* and *O. purpureiflora*, Table S26) and less translocation and duplication rearrangements with them, which agree with our (Fig. 3C) and previous phylogeny results [1]. *Ormosia emarginata* and *O. semicastrata* were in different clades [1]. However, although *O. purpureiflora* was sister to *O. emarginata* and therefore in the same clade as *O. emarginata*, it seemed that the structural rearrangements between *O. emarginata* and *O. semicastrata* were not maintained in *O. purpureiflora*.

Ormosia purpureiflora and *O. emarginata* displayed the largest inversion on chromosome 1, from 166,804,741 to 222,962,103 bp in *O. purpureiflora* and from 127,118,909 to 186,491,244 bp in *O. emarginata*. On the same chromosome, extensive duplications occurred. On chromosome 2, it exhibited extensive translocations. On this chromosome, an *O. purpureiflora*-specific inverted region was identified from 52,506,652 to 61,757,520 bp (Fig. 3F and Fig. 3S), which was far from the rearrangement hot region. The inverted region was 9,250,868 bp in length, including 577 genes.

Enrichment analysis indicated that these genes were related to osmotic stress and temperature (Table S27), which may facilitate its adaptation in rocky (Fig. 1F), thin soil layer (low soil moisture content) and relatively high elevation (400–750 m in altitude) environments (E-P Yu, personal observation).

Interestingly, hot structural rearrangement regions on the chromosomes of *Ormosia* were clearly correlated to the highest repeat density areas of *Gypsy* and unknown LTR-RTs (Fig. 4), indicating repeat-mediated rearrangements in *Ormosia*. Since three *Ormosia* species displayed similar patterns on the chromosomes, such rearrangements may have been inherited from their ancient ancestors. The other LTR-TR types of *Copia* were generally evenly distributed along the chromosomes in *Ormosia*. Enrichment analysis indicated *Copia*-mediated genes mainly related to mitosis, mismatch repair and secondary metabolite biosynthetic process in GO's BP category (Table S28) and glutathione metabolism, transcription machinery, replication and repair, and chromosome and associated proteins in KEGG (Table S29), although these results were not significant after *p*-value correction procedures.

Genomic rearrangements are an important force of evolution [94]. However, they have been less investigated in Fabaceae [95]. In *Eucalyptus*, duplications and translocations contribute the most to genome divergence [96]. Mechanisms causing the rearrangement may include recombination, repair or replication and are closely related to repetitive elements [97, 98]. Clearly, hot rearrangement regions and their relationship with LTR-RTs in *Ormosia* have suggested that they can be chosen as a model system for such mechanism studies in the future.

RNA-seq analysis of tissues

RNA-seq analysis showed that 28,504 protein-coding genes were expressed in all examined tissues (Fig. S5). Flower tissues contained the most abundant specifically expressed genes (5104) and the leaf tissue followed with 3275.

Compared to the other three tissues, 266 genes were highly significantly (adjusted $P<0.05$) expressed in leaf tissue, and 1388 showed significantly less expression (Table S30). Enrichment analysis indicated the highly expressed genes were mainly related to photosynthesis in GO's BP category and KEGG (Table S31 and S32), and low expressed ones were related to lipid storage and cell wall in GO's BP category (Table S33) and carbohydrate metabolism, structural proteins,

anthocyanin biosynthesis, glycosylphosphatidylinositol (GPI)-anchored proteins, and cutin, suberine and wax biosynthesis in KEGG (Table S34).

In flower tissue, the number of highly expressed genes was 146 (Table S35), and they were mainly related to cell wall and pentose and glucuronate interconversions (Table S36 and S37). The number of lowly expressed genes was 155, which were mainly related to lipid storage and glycolysis/gluconeogenesis (Table S38 and S39).

In fruit tissue, 130 genes were highly significantly expressed (Table S40), and their main functions were related to abscisic acid and phenylpropanoid biosynthesis (Table S41 and S42). A total of 1275 genes were lowly significantly expressed, and their functions were mainly related to cell growth and autophagy in GO's BP category (Table S43) and protein families of genetic information processing in KEGG (Table S44).

There were 630 genes highly significantly expressed in seed tissue (Table 45), which were mainly related to lipid storage and transcription factors (Table S46 and S47). There were 1454 genes lowly significantly expressed in seed tissue, and they were mainly related to transcription factor activity regulation and ammonium transmembrane transport in GO's BP category (Table S48) and polyketide biosynthesis proteins and flavone and flavonol biosynthesis (Table S49).

Nucleotide binding leucine-rich repeats (NLR) and the other resistance (*R*) gene identification

Compared to the other species, *Ormosia* species contained a substantially high number of *R* genes (Table S50). *Ormosia* species contained more, in numbers and percentage, NLR genes than in the sister species of *L. albus*. For the other *R* genes, *L. albus* showed a higher number/percentage than *Ormosia* species. *Ormosia purpureiflora* contained a higher number/percentage of the other *R* genes than *O. emarginata* and *O. semicastrata*. The distribution of *R* genes in the chromosomes in each *Ormosia* species is shown in Fig. S6. *R* genes were distributed across all eight chromosomes, matching their whole gene distributions in *Ormosia* species.

The highly and lowly expressed *R* gene numbers in fruit tissues were 14 and 81, which were 10.77% (14/130) and 6.35% (81/1275) of the total highly and lowly expressed protein-coding genes in this tissue. Both percentages were highest compared to such percentage of the other tissues (2.05–5.64% of highly expressed genes, and 4.25–5.09% of lowly expressed genes) (Table S51).

Transcription factor

Ormosia had a high number of TF genes in all compared species except lower than *Glycine max*, *Sesbania bispinosa, Acacia pycnantha* and their sister species *L. albus*, but the percentage of TF genes in *Ormosia* was low, especially in *O. purpureiflora* (3.96%, Table S52).

In *O. purpureiflora*, the highly expressed TF gene number in leaf tissue was 31, which was 11.65% of the total highly expressed protein-coding genes in this tissue, and this percentage was highest compared to the other tissues (Table S53). The highly/lowly expressed TF genes in *O. purpureiflora* in different tissues are shown in Tables S54–S57. For example, the highly expressed TF genes in leaf tissue were mainly WRKY, ERF, MIKC_MADS and MYB_related types (Table S54).

SNP calling and genetic structure

A total of 276,854 high quality, biallelic and no genotype missing SNPs were obtained from 153 *O. purpureiflora* individuals. PCAdapt indicated that four main components were suitable to account for population structure in the Scree plot (Fig. S7). PCAdapt then revealed 4661 candidate SNPs putatively under selection. BayPass revealed 12,639 candidate SNPs. Across the two results, there were 2632 SNPs identified as adaptive SNPs. Among these SNPs, 104 appeared in 58 genes. Enrichment analysis in KEGG indicated that they were mainly related to carbohydrate metabolism (Table S58).

After removing adaptive SNPs, 274,222 remained for genetic structure analyses. PCA revealed that the first principal component divided LFS4 from the other (sub)populations (Fig. 2B). The second principal component further separated LFS5 and NKS from the others. The third principal component confirmed that the NKS population was distinct from LFS populations. The value of CV errors in the ADMIXTURE analysis was reduced constantly from K=1 to K=6 (Fig. S8). However, from K=4, the decrease tended to slow down. Therefore, the results were shown for K=2–4 in this study (Fig. 2C). When K=2, individuals in LFS4 were separated from the others. At K=3, individuals in LFS5 were further separated. At K=4, NKS was separated as a distinct group. Both PCA and ADMIXTURE indicated the distinctiveness of LFS4; however, the reasons for its

distinctiveness were unclear.

Genetic diversity

Genetic diversity analyses in *O. purpureiflora* (sub)populations indicated that LFS1 contained the highest genetic diversity for all three parameters (*Ho*, *He* and π) (Table 1). The lowest *He* and π were observed in LFS4 (0.341 and 0.349, respectively), while the lowest *Ho* occurred in LFS5 (0.531). All (sub)populations showed negative *Fis*, indicating heterozygosity excess. The overall *Fst* was 0.047, which was low. Compared to *O. henryi*, which was more widely distributed in southern China, *O. purpureiflora* displayed higher genetic diversity but lower *Fis* (*Ho*: 0.228–0.287, *He*: 0.237–0.290, π: 0.122–0.143, *Fis*: −0.023–0.022 in *O. henryi*) [99]. This indicates that *O. purpureiflora* displays higher outcrossing.

All genetic diversity parameters along the chromosomes showed that the region from approximately 39 to 68 Mbp on chr5 had relatively higher π, dxy, *Ho* and *He* values but a lower *Fst* value (Fig. 2D). The genes in this region were mainly functionally related to glycosyltransferases, translation and carbohydrate metabolism in KEGG enrichment analysis but showed no significance (Table S59). Glycosyltransferases drive the glycosylation reactions, and glycosylation is an important biomolecule modification to transfer monosaccharides from sugar donors to glycans, lipids, peptides and small molecules [100–102]. These biomolecules participate in a variety of biological processes, playing crucial roles in plant growth, development, defense and stress tolerance [102–104].

The low *Fst* value and high values for other genetic diversity parameters could be explained by balancing selection on this region. Balancing selection maintains allelic diversity in the population and is an important driver for species evolutionary adaptation [105, 106]. Very few studies have investigated balancing selection in Fabaceae [107–110]. *Cicer arietinum* showed that 4.8% of its genome was probably under balancing selection, and the major genes under selection were NBS-LRR disease resistance genes [108]. In *O. purpureiflora* and other *Ormosia* species, further summary statistics and model-based tests [111] are required to detect and confirm the signatures of balancing selection from the genome scale.

Genetic relatedness and fine-scale spatial genetic structure

Pairwise relatedness among the individuals was shown by a heatmap (Fig. S9), which revealed that individuals of LFS4, LFS5 and NKS were more genetically related to themselves than the individuals of the other (sub)populations and indicated some isolation.

Significant fine-scale SGSs with positive relatedness coefficients were observed in LFS3, LFS5 and NKS but only at their first distance classes of 5, 5 and 10 m (Fig. S10). For the other three subpopulations, no significant SGSs were detected in any of the distance classes. *Ormosia* seeds are mainly dispersed by gravity [112], and not frequently by rats or birds [113, 114], which should restrict their distribution and might facilitate SGS formation. Therefore, weak or absent SGSs indicate high gene flow over greater distance via pollen in *Ormosia*, as they are insect pollinated species [112]. It has been suggested that pollen dispersal is often the major contributor to gene flow, making SGS undetectable [115].

Conclusion

Fabaceae play a key role in biological nitrogen fixation and form nutritious food sources for wild fauna. They are beneficial for the health and balance of the ecosystem, which is also held in *Ormosia* species. Previous studies have revealed that *Ormosia* species contain plentiful secondary metabolites, including alkaloids, terpenes and flavonoids, and merited deep exploration, especially from the genomic aspect. The previous two *Ormosia* genomes and the current *O. purpureiflora* genome indicate that the genes involved in the biosynthesis of these metabolites are in TD, PD duplications or expanded. The gene-repeat distribution association implies that repeats might also activate their duplication, suggesting a future study. Therefore, the high-quality *Ormosia* genomes provide valuable resources to decipher the efficiency of biosynthesis and identify more potentially useful chromosome regions (synteny, occurring structural rearrangement and balancing selection) for study in the future.

Funding:

The study is supported by Guangdong Science and Technology Plan Project (2023A1111110001); Key-Area Research and Development Program of Guangdong Province

(2022B1111230001) and its sub-project (2022B1111230001-2-5); Guangdong Provincial Forestry Bureau Project — Planning of the Provincial Plant Ex Situ Protection System and National Key Protected Plant Ex Situ Protection and Propagation; The National Natural Science Foundation of China (No. 32370406, 31970188); Guangdong Science and Technology Plan Project (grant No.: 2023B1212060046).

Data Availability

Raw sequenced reads have been uploaded to the NCBI Sequence Read Archive under accession number of SRR24060960 for short WGS reads, SRR24061088 and SRR24061087 for long WGS reads, SRR24085385 for ultralong WGS reads, SRR24112497 for Hi-C reads, SRR24044811 for fruit RNA-seq reads, SRR24044812 for seed RNA-seq reads, SRR24085891 for leaf RNA-seq reads, SRR24085890 for flower RNA-seq reads in *O. purpureiflora*; SRR25460826 for Hi-C reads of *O. emarginata*; SRR25460825 for Hi-C reads for *O. semicastrata*; SRR29820911-SRR29820936 for resequencing reads of LFS1, SRR29824870-SRR29824895 for resequencing reads of LFS2, SRR29837260-SRR29837285 for resequencing reads of LFS3, SRR29856316-SRR29856341 for resequencing reads of LFS4, SRR29887191-SRR29887216 for resequencing reads of LFS5, SRR29761002-SRR29761004, SRR29761010-SRR29761017, SRR29761028-SRR29761030, SRR29761107, SRR29761108, SRR29761114, SRR29761115, SRR29761118, SRR29761123, SRR29761124, SRR29761126, SRR29761139 for resequencing reads of NKS in *O. purpureiflora*. Assembled genomes are under accession number of GCA_040955955.1 for *O. purpureiflora*, GCA_029884595.2 for *O. semicastrata* and GCA_029884605.2 for *O. emarginata*. Annotations, SNPs and the other files are submitted to figshare (https://doi.org/10.6084/m9.figshare.26826466.v1).

Competing Interests

The authors declare that they have no competing interests.

References

1. Torke BM, Cardoso D, Chang H, et al. A dated molecular phylogeny and biogeographical

analysis reveals the evolutionary history of the trans-pacifically disjunct tropical tree genus *Ormosia* (Fabaceae). Mol Phylogenet Evol 2022;166:107329. https://doi.org/10.1016/j.ympev.2021.107329.

2. Niu M, Jiang K-W, Song Z-Q, et al. Two new synonyms of *Ormosia semicastrata* (Fabaceae, Papilionoideae, Ormosieae). Phytotaxa 2023;613(2):140-152. https://doi.org/10.11646/phytotaxa.613.2.3.
3. Wang Z, Shi G, Sun B, et al. A new species of *Ormosia* (Leguminosae) from the middle Miocene of Fujian, Southeast China and its biogeography. Rev Palaeobot Palyno 2019;270:40-47. https://doi.org/10.1016/j.revpalbo.2019.07.003.
4. Li L, Lei M, Wang H, et al. First report of dieback caused by *Lasiodiplodia pseudotheobromae* on *Ormosia pinnata* in China. Plant Dis 2020;104:2551-2555. https://doi.org/10.1094/PDIS-03-20-0647-RE.
5. Wei L, Wang G, Xie C. Predicting suitable habitat for the endangered tree *Ormosia microphylla* in China. Sci Rep 2024;14:10330. https://doi.org/10.1038/s41598-024-61200-5.
6. Zhang L-J, Zhou W-J, Ni L, et al. A review on chemical constituents and pharmacological activities of *Ormosia*. Chin Tradit Herbal Drugs, 2021;52(14):4433-4442. https://doi.org/10.7501/j.issn.0253-2670.2021.14.035.
7. Zhou Q-Q , Xie X-Y, Zhu J-W, et al. Hosimosines A-E, structurally diverse cytisine derivatives from the seeds of *Ormosia hosiei* Hemsl. et Wils. Fitoterapia, 2023;170:105661. https://doi.org/10.1016/j.fitote.2023.105661.
8. Zhou W, Quan Y, Chen Y, et al. A new lignan from leaves of *Ormosia xylocarpa*. Rec Nat Prod 2023;17(1):189-194. http://doi.org/10.25135/rnp.338.2203.2386.
9. Wang J, Li L, Wang Z et al. Integrative analysis of the metabolome and transcriptome reveals the molecular regulatory mechanism of isoflavonoid biosynthesis in *Ormosia henryi* Prain. Int J Biol Macromol 2023;246:125601. https://doi.org/10.1016/j.ijbiomac.2023.125601.
10. Wang J, Wang X, Deng X, et al. Analysis of candidate genes for terpene synthesis in *Ormosia henryi* based on metabolome and transcriptome. J Zhejiang A&F Univ 2023;40(5):970-981. https://doi.org/10.11833/j.issn.2095-0756.2022073.
11. Liu P-P, Yu E-P, Tan Z-J et al. Genome assemblies of two *Ormosia* species: Gene duplication

related to their evolutionary adaptation. Agronomy 2023;13:1757. https://doi.org/10.3390/agronomy13071757.

12. Joshi NA, Fass JN. Sickle: A sliding-window, adaptive, quality-based trimming tool for FastQ files (Version 1.33). 2011. https://github.com/najoshi/sickle. Accessed 3 September 2021.
13. Długosz M, Deorowicz S. RECKONER: read error corrector based on KMC. Bioinformatics 2017;33:1086-1089. https://doi.org/10.1093/bioinformatics/btw746.
14. Marçais G, Kingsford C. A fast, lock-free approach for efficient parallel counting of occurrences of k-mers. Bioinformatics 2011;27:764-770. https://doi.org/10.1093/bioinformatics/btr011.
15. Vurture GW, Sedlazeck FJ, Nattestad M et al. GenomeScope: fast reference-free genome profiling from short reads. Bioinformatics 2017;33:2202-2204. https://doi.org/10.1093/bioinformatics/btx153.
16. Porechop (Version 0.2.4). https://github.com/rrwick/Porechop/releases/tag/v0.2.4. Accessed 8 January 2019.
17. Hu J, Wang Z, Sun Z, et al. NextDenovo: an efficient error correction and accurate assembly tool for noisy long reads. Genome Biol 2024;25:107 (2024). https://doi.org/10.1186/s13059-024-03252-4.
18. Pseudohaploid. https://github.com/schatzlab/pseudohaploid. Accessed 28 August 2020.
19. Guan DF, McCarthy SA, Wood J, et al. Identifying and removing haplotypic duplication in primary genome assemblies. Bioinformatics 2020;36:2896-2898. https://doi.org/10.1093/bioinformatics/btaa025.
20. Vaser R, Sović I, Nagarajan N, et al. Fast and accurate de novo genome assembly from long uncorrected reads. Genome Res 2017;27(5):737-746. https://doi.org/10.1101/gr.214270.116.
21. Aury JM, Istace B. Hapo-G, haplotype-aware polishing of genome assemblies with accurate reads. NAR Genom Bioinform 2021;3(2):lqab034. https://doi.org/10.1093/nargab/lqab034.
22. Wick RR, Holt KE. Polypolish: short-read polishing of long-read bacterial genome assemblies. PLoS Comput Biol 2022;18(1):e1009802. https://doi.org/10.1371/journal.pcbi.1009802.
23. Depthcharge v0.2.0. https://github.com/slimsuite/depthcharge. Accessed 28 January 2023.
24. Scaffhic v1.1. https://github.com/wtsi-hpag/scaffHiC. Accessed 7 December 2022.

25. Durand NC, Shamim MS, Machol I, et al. Juicer provides a one-click system for analyzing loop-resolution Hi-C experiments. Cell Syst 2016;3(1):95-98. https://doi.org/10.1016/j.cels.2016.07.002.

26. Dudchenko O, Batra SS, Omer AD, et al. De novo assembly of the *Aedes aegypti* genome using Hi-C yields chromosome-length scaffolds. Science 2017;356(6333):92-95. https://doi.org/10.1126/science.aal3327.

27. Xu M, Guo L, Gu S, et al. TGS-GapCloser: A fast and accurate gap closer for large genomes with low coverage of error-prone long reads. Gigascience 2020;9(9):giaa094. https://doi.org/10.1093/gigascience/giaa094.

28. Leszek P. Pryszcz, Toni Gabaldón, Redundans: an assembly pipeline for highly heterozygous genomes, Nucleic Acids Res. 2016;44(12):e11. https://doi.org/10.1093/nar/gkw294.

29. Teloclip v0.0.3. https://github.com/Adamtaranto/teloclip. Accessed 28 March 2023.

30. Seppey M, Manni M, Zdobnov EM. BUSCO: Assessing genome assembly and annotation completeness. Methods Mol Biol 2019;1962:227-245. https://doi.org/10.1093/bioinformatics/btv351.

31. Ou S, Su W, Liao Y, et al. Benchmarking transposable element annotation methods for creation of a streamlined, comprehensive pipeline. Genome Biol 2019;20:275. https://doi.org/10.1186/s13059-019-1905-y.

32. Girgis HZ. Red: an intelligent, rapid, accurate tool for detecting repeats de-novo on the genomic scale. BMC Bioinform 2015;16(1):227. https://doi.org/10.1186/s12859-015-0654-5.

33. Quinlan AR, Hall IM. BEDTools: a flexible suite of utilities for comparing genomic features. Bioinformatics, 2010;26(6):841-842. https://doi.org/10.1093/bioinformatics/btq033.

34. Brůna T, Hoff KJ, Lomsadze A, et al. (2021) BRAKER2: automatic eukaryotic genome annotation with GeneMark-EP+ and AUGUSTUS supported by a protein database. NAR Genom Bioinform 2021;3(1):lqaa108. https://doi.org/10.1093/nargab/lqaa108.

35. Funannotate v1.8.16. https://github.com/nextgenusfs/funannotate. Accessed 12 March 2023.

36. Zhang H, Tanner Y, Huang L, et al. dbCAN2: a meta server for automated carbohydrate-active enzyme annotation. Nucleic Acids Res, 2018;46:W95-W101. https://doi.org/10.1093/nar/gky418.

37. Huerta-Cepas J, Forslund K, Coelho LP, et al. Fast genome-wide functional annotation through orthology assignment by eggNOG-mapper. Mol Biol Evol 2017;34:2115-2122. https://doi.org/10.1093/molbev/msx148.

38. The Gene Ontology Consortium. The gene ontology resource: 20 years and still GOing strong. Nucleic Acids Res 2019;47(D1):D330-D338. https://doi.org/10.1093/nar/gky1055.

39. Ashburner M, Ball CA, Blake JA, et al. Gene ontology: tool for the unification of biology. Nat Genet 2000;25:25-29. https://doi.org/10.1038/75556.

40. Kanehisa M, Soto Y, Kawashima M, et al. KEGG as a reference resource for gene and protein annotation. Nucleic Acids Res 2016;44(D1):D457-D462. https://doi.org/10.1093/nar/gkv1070.

41. Mitchell AL, Attwood TK, Babbitt PC, et al. InterPro in 2019: improving coverage, classification and access to protein sequence annotations. Nucleic Acids Res 2019;47(D1): D351-D360. https://doi.org/10.1093/nar/gky1100.

42. Rawlings ND, Barrett AJ, Thomas PD, et al. The merops database of proteolytic enzymes, their substrates and inhibitors in 2017 and a comparison with peptidases in the PANTHER database. Nucleic Acids Res 2018;46(D1):D624-D632. https://doi.org/10.1093/nar/gkx1134.

43. El-Gebali, S., Mistry, J., Bateman, A., et al. The Pfam protein families database in 2019. Nucleic Acids Res 2019;47(D1):D427-D432. https://doi.org/10.1093/nar/gky995.

44. Almagro Armenteros JJ, Tsirigos KD, Sønderby CK et al. SignalP 5.0 improves signal peptide predictions using deep neural networks. Nat Biotechnol 2019;37(4):420-423. https://doi.org/10.1038/s41587-019-0036-z.

45. The UniProt Consortium. UniProt: a worldwide hub of protein knowledge. Nucleic Acids Res 2019;47(D1):D506-D515. https://doi.org/10.1093/nar/gky1049.

46. Moriya Y, Itoh M, Okuda S, et al. KAAS: an automatic genome annotation and pathway reconstruction server. Nucleic Acids Res 2007;35:W182-W185. https://doi.org/10.1093/nar/gkm321.

47. Emms DM, Kelly S. OrthoFinder: solving fundamental biases in whole genome comparisons dramatically improves orthogroup inference accuracy. Genome Biol 2015;16:157. https://doi.org/10.1186/s13059-015-0721-2.

48. Emms DM, Kelly S. OrthoFinder: phylogenetic orthology inference for comparative genomics.

Genome Biol 2019;20:238. https://doi.org/10.1186/s13059-019-1832-y.

49. Emms DM, Kelly S. STAG: Species tree inference from all genes. bioRxiv 2018. https://doi.org/10.1101/267914.

50. Emms DM, Kelly S. STRIDE: species tree root inference from gene duplication events. Mol Biol Evol 2017;34:3267-3278. https://doi.org/10.1093/molbev/msx259.

51. Han MV, Thomas GWC, Jose LM, et al. Estimating gene gain and loss rates in the presence of error in genome assembly and annotation using cafe 3. Mol Biol Evol 2013;30(8):1987-1997. https://doi.org/10.1093/molbev/mst100.

52. dos Reis M, Zhu T, Yang Z. The impact of the rate prior on Bayesian estimation of divergence times with multiple loci. System Biol 2014;63:555-565. https://doi.org/10.1093/sysbio/syu020.

53. Chen CJ, Chen H, Zhang Y, et al. TBtools - an integrative toolkit developed for interactive analyses of big biological data. Mol Plant 2020;13(8):1194-1202. https://doi.org/10.1016/j.molp.2020.06.009.

54. Zwaenepoel A, de Peer YV. wgd-simple command line tools for the analysis of ancient whole-genome duplications. Bioinformatics 2019;35:2153-2155. https://doi.org/10.1093/bioinformatics/bty915.

55. Almeida-Silva F, Van de Peer Y. doubletrouble: Identification and classification of duplicated genes. R package version 0.99.1. 2022. https://github.com/almeidasilvaf/doubletrouble. Accessed 28 March 2023.

56. Qiao X, Li QH, Yin H, et al. Gene duplication and evolution in recurring polyploidization–diploidization cycles in plants. Genome Biol 2019;20:38. https://doi.org/10.1186/s13059-019-1650-2.

57. MCScanX. https://github.com/wyp1125/MCScanX. Accessed 28 July 2023.

58. Wang Y, Jia L, Tian G. et al. shinyCircos-V2.0: Leveraging the creation of Circos plot with enhanced usability and advanced features. iMeta 2023;2(2):e109. https://doi.org/10.1002/imt2.109.

59. Bandi V, Gutwin C. 2020. Interactive exploration of genomic conservation. In Proceedings of the 46th Graphics Interface Conference on Proceedings of Graphics Interface 2020 (GI'20). Canadian Human-Computer Communications Society, Waterloo, CAN.

60. Pérez-Wohlfeil E, Diaz-del-Pino S, Trelles O. Ultra-fast genome comparison for large-scale genomic experiments. Sci rep 2019;9:10274. https://doi.org/10.1038/s41598-019-46773-w.
61. Goel M, Schneeberger K. plotsr: visualizing structural similarities and rearrangements between multiple genomes, Bioinformatics 2022;38(10): 2922-2926. https://doi.org/10.1093/bioinformatics/btac196.
62. Patel H, Ewels P, Peltzer A, et al. nf-core/rnaseq: nf-core/rnaseq v3.14.0 - Hassium Honey Badger. 10.5281/zenodo.1400710. April 1, 2024. https://doi.org/10.5281/zenodo.1400710.
63. WackerO, Manning J, Zoufir A, et al. nf-core/differentialabundance: v1.4.0 - 2023-11-27. 10.5281/zenodo.7568000. April 1, 2024. https://doi.org/10.5281/zenodo.7568000.
64. Shao Z-Q, Xue J-Y, Wu P, et al. Large-scale analyses of angiosperm Nucleotide-Binding Site-Leucine-Rich Repeat genes reveal three anciently diverged classes with distinct evolutionary patterns. Plant Physiol 2016;170(4):2095-2109. https://doi.org/10.1104/pp.15.01487.
65. Chou WC, Jha S, Linhoff MW et al. The NLR gene family: from discovery to present day. Nat Rev Immunol 2023;23,635-654. https://doi.org/10.1038/s41577-023-00849-x.
66. Santos MdL, Resende MLV, Alves GSC, et al. Genome-wide identification, characterization, and comparative analysis of NLR resistance genes in *Coffea* spp.. Front Plant Sci 2022;13:868581. https://doi.org/10.3389/fpls.2022.868581.
67. Liu Y, Zhang Y-M, Tang Y, et al. The evolution of plant NLR immune receptors and downstream signal components. Curr Opin Plant Biol 2023;73:102363. https://doi.org/10.1016/j.pbi.2023.102363.
68. De-la-Cruz IM, Hallab A, Olivares-Pinto U, et al. Genomic signatures of the evolution of defence against its natural enemies in the poisonous and medicinal plant *Datura stramonium* (Solanaceae). Sci Rep 2021;11:882. https://doi.org/10.1038/s41598-020-79194-1.
69. Tian F, Yang DC, Meng YQ, et al. PlantRegMap: charting functional regulatory maps in plants. Nucleic Acids Res 2019;48(D1):D1104-D1113. https://doi.org/10.1093/nar/gkz1020.
70. Puritz JB, Hollenbeck CM, Gold JR. Docent: a RADseq, variant-calling pipeline designed for population genomics of non-model organisms. PeerJ 2014;2:e431 https://doi.org/10.7717/peerj.431.
71. Liu H-L, Harris AJ, Wang Z-F, et al. The genome of the Paleogene relic tree *Bretschneidera*

sinensis: insights into trade-offs in gene family evolution, demographic history, and adaptive SNPs. DNA Res 2022;29(1):dsac003. https://doi.org/10.1093/dnares/dsac003.

72. Danecek P, Auton A, Abecasis G, et al. The variant call format and VCFtools. Bioinformatics. 2011;27(15):2156-2158. https://doi.org/10.1093/bioinformatics/btr330.
73. Luu K, Bazin E, Blum MG. pcadapt: an R package to perform genome scans for selection based on principal component analysis, Mol Ecol Resour 2017;17:67-77. https://doi.org/10.1111/1755-0998.12592.
74. Privé F, Luu K, Vilhja´lmsson BJ, et al. Performing highly efficient genome scans for local adaptation with R package pcadapt version 4. Mol Biol Evol 2020;37:2153–2154. https://doi.org/10.1093/molbev/msaa053.
75. Gautier M. Genome-wide scan for adaptive divergence and association with population-specific covariates. Genetics 2015;201:1555-1579. https://doi.org/10.1534/genetics.115.181453.
76. Alexander DH, Novembre J, Lange K. Fast model-based estimation of ancestry in unrelated individuals. Genome Res 2009;19:1655-1664. https://doi.org/10.1101/gr.094052.109.
77. Zheng X, Levine D, Shen J, et al. A high-performance computing toolset for relatedness and principal component analysis of SNP data. Bioinformatics 2012;28(24):3326-3328. doi:10.1093/bioinformatics/bts606.
78. Mussmann S, Douglas MR, Chafin T, et al. AdmixPipe: population analyses in Admixture for non-model organisms, BMC Bioinform 2020;21:337. https://doi.org/10.1186/s12859-020-03701-4.
79. Kopelman NM, Mayzel J, et al. Clumpak: a program for identifying clustering modes and packaging population structure inferences across K. Mol Ecol Resour 2015;15:1179-1191. https://doi.org/10.1111/1755-0998.12387.
80. Korunes KL, Samuk K. pixy: Unbiased estimation of nucleotide diversity and divergence in the presence of missing data. Mol Ecol Resour 2021;21:1359-1368. https://doi.org/10.1111/1755-0998.13326.
81. Lynch M, Ritland K. Estimation of pairwise relatedness with molecular markers. Genetics 1999; 152(4):1753-1766. doi: 10.1093/genetics/152.4.1753.

82. Wang, J. Fast and accurate population admixture inference from genotype data from a few microsatellites to millions of SNPs. Heredity 2022;129:79-92. https://doi.org/10.1038/s41437-022-00535-z.

83. Peakall R, Smouse PE. GenAlEx 6.5: genetic analysis in Excel. Population genetic software for teaching and research--an update. Bioinformatics 2012:28(19):2537-2539. https://doi.org/10.1093/bioinformatics/bts460.

84. Xu W, Zhang Q, Yuan W, et al. The genome evolution and low-phosphorus adaptation in white lupin. Nat Commun 2020;11:1069. https://doi.org/10.1038/s41467-020-14891-z.

85. Lai X, Schnable JC, Liao Z. et al. Genome-wide characterization of non-reference transposable element insertion polymorphisms reveals genetic diversity in tropical and temperate maize. BMC Genomics 2017;18:702. https://doi.org/10.1186/s12864-017-4103-x.

86. Chen D, Wang Y, Zhang W, et al. Gametophyte-specific DEAD-box RNA helicase 29 is required for functional maturation of male and female gametophytes in Arabidopsis, J Exp Bot 2020;71:4083-4092. https://doi.org/10.1093/jxb/eraa190.

87. Baldo AM, McClure MA. Evolution and horizontal transfer of dUTPase-encoding genes in viruses and their hosts. J Virol 1999;73:7710-7721. https://doi.org/10.1128/jvi.73.9.7710-7721.1999.

88. Zhu S, Tan Z, Guo Z, et al. Symbiotic virus-bacteria interactions in biological treatment of coking wastewater manipulating bacterial physiological activities. Water Res, 2024;257:121741. https://doi.org/10.1016/j.watres.2024.121741.

89. Zhang H, Zhang TT, Liu H, et al. Thioredoxin-mediated ROS homeostasis explains natural variation in plant regeneration. Plant Physiol 2018;76(3):2231-2250. https://doi.org/10.1104/pp.17.00633.

90. Kushwaha R, Lloyd TD, Schafermeyer KR, et al. Identification of late embryogenesis abundant (LEA) protein putative interactors using phage display. Int J Mol Sci 2012;13:6582-603. https://doi.org/10.3390/ijms13066582.

91. Zhang M, Zhong X, Li M, et al. Genome-wide analyses of the Nodulin-like gene family in bread wheat revealed its potential roles during arbuscular mycorrhizal symbiosis. Int J Biol Macromol 2022;201:424-436. https://doi.org/10.1016/j.ijbiomac.2022.01.076.

92. Xiao H, Zhang Y, Wang M. Discovery and engineering of cytochrome P450s for terpenoid biosynthesis. Trends Biotechnol 2019;37:618-631. https://doi.org/10.1016/j.tibtech.2018.11.008.

93. Zheng X, Li P, Lu X. Research advances in cytochrome P450-catalysed pharmaceutical terpenoid biosynthesis in plants. J Exp Bot 2019;70:4619-4630. https://doi.org/10.1093/jxb/erz203.

94. Feulner PGD, De-Kayne R. Genome evolution, structural rearrangements and speciation. J Evol Biol 2017;30(8):1488-1490. https://doi.org/10.1111/jeb.13101.

95. Nascimento T, Pedrosa-Harand A. High rates of structural rearrangements have shaped the chromosome evolution in dysploid *Phaseolus* beans. Theor Appl Genet 2023;136:215. https://doi.org/10.1007/s00122-023-04462-3.

96. Ferguson S, Jones A, Murray K, et al. Plant genome evolution in the genus Eucalyptus is driven by structural rearrangements that promote sequence divergence. Genome Res 2024; 34(4):606-619. https://doi.org/10.1101/gr.277999.123.

97. Burssed B, Zamariolli M, Bellucco FT, et al. Mechanisms of structural chromosomal rearrangement formation. Mol Cytogenet 2022;15:23. https://doi.org/10.1186/s13039-022-00600-6

98. Hassan AH, Mokhtar MM, El Allali A . Transposable elements: multifunctional players in the plant genome. Front. Plant Sci. 2024;14:1330127. doi: https://doi.org/10.3389/fpls.2023.1330127.

99. Zhou C, Xia S, Wen Q, et al. Genetic structure of an endangered species *Ormosia henryi* in southern China, and implications for conservation. BMC Plant Biol 2023;23:220. https://doi.org/10.1186/s12870-023-04231-w.

100. Jones P, Vogt T. Glycosyltransferases in secondary plant metabolism: tranquilizers and stimulant controllers. Planta 2001;213:164-174. https://doi.org/10.1007/s004250000492.

101. Keegstra K, Raikhel N. Plant glycosyltransferases. Curr Opin Plant Biol 2001;4(3):219-224. https://doi.org/10.1016/s1369-5266(00)00164-3.

102. Bowles D, Isayenkova J, Lim E-K, et al. Glycosyltransferases: managers of small molecules. Curr Opin Plant Biol 2005;8(3):254-263. https://doi.org/10.1016/j.pbi.2005.03.007.

103. Wang J, Hou B. Glycosyltransferases: key players involved in the modification of plant secondary metabolites. Front Biol China 2009;4:39-46. https://doi.org/10.1007/s11515-008-0111-1.
104. Gharabli H, Gala VD, Welner DH. The function of UDP-glycosyltransferases in plants and their possible use in crop protection. Biotechnol Adv 2023;67:108182. https://doi.org/10.1016/j.biotechadv.2023.108182.
105. Delph LF, Kelly JK. On the importance of balancing selection in plants. New Phytol 2013;201(1):45-56. https://doi.org/10.1111/nph.12441 .
106. Llaurens V, Whibley A, Joron M. Genetic architecture and balancing selection: the life and death of differentiated variants. Mol Ecol 2017;26(9):2430-2448. https://doi.org/10.1111/mec.14051.
107. Olsen KM, Kooyers NJ, Small LL. Recurrent gene deletions and the evolution of adaptive cyanogenesis polymorphisms in white clover (*Trifolium repens* L.). Mol Ecol 2012;22(3):724-738. https://doi.org/10.1111/j.1365-294x.2012.05667.x PMID: 22694056
108. Varshney RK, Song C, Saxena RK, et al. Draft genome sequence of chickpea (*Cicer arietinum*) provides a resource for trait improvement. Nat Biotechnol 2013;31:240-246. https://doi.org/10.1038/nbt.2491.
109. Epstein B, Burghardt LT, Heath KD, et al. Combining GWAS and population genomic analyses to characterize coevolution in a legume-rhizobia symbiosis. Mol Ecol 2022;32:3798-3811. https://doi.org/10.1111/mec.16602.
110. Wang X, Choi, Y-M, Jeon Y-A, et al. Analysis of genetic diversity in adzuki Beans (*Vigna angularis*): Insights into environmental adaptation and early breeding strategies for yield improvement. Plants 2023;12:4154. https://doi.org/10.3390/plants12244154.
111. Bitarello BD, Brandt DYC, Meyer D, et al. Inferring balancing selection from genome-scale data. Genome Biol Evol 2023;15(3):evad032. https://doi.org/10.1093/gbe/evad032.
112. Li F, Chen H, Liu S, et al. Mating systems of single families and population genetic diversity of endangered *Ormosia hosiei* in South China. Genes 2022;13:2117. https://doi.org/10.3390/genes13112117.
113. Liu X, Wang Z, Xiao Z. Patterns of seed predation and dispersal of an endangered rare plant

Ormosia hosiei by Edward's long-tailed rats and Chinese white-bellied rats. Biodiversity, 2011;19 (1):93-96. https://doi.org/10.3724/SP.J.1003.2011.04197.

114. Foster MS, Delay LS. Dispersal of mimetic seeds of three species of *Ormosia* (Leguminosae). J Trop Ecol 1998;14(4):389-411. doi:10.1017/S0266467498000303.

115. Hardy OJ, González-Martínez SC, Fréville H, et al. Fine-scale genetic structure and gene dispersal in *Centaurea corymbosa* (Asteraceae) I. Pattern of pollen dispersal. J Evol Biol 2004;17(4):795-806. https://doi.org/10.1111/j.1420-9101.2004.00713.x.

Table 1. Six sampled (sub)populations and their genetic diversities in *Ormosia purpureiflora*

(Sub)population	Sample size	*Ho*	*He*	*Fis*	*π*
LFS1	26	0.561	0.365	-0.511	0.373
LFS2	26	0.547	0.357	-0.473	0.364
LFS3	26	0.545	0.363	-0.466	0.371
LFS4	26	0.541	0.341	-0.457	0.349
LFS5	26	0.531	0.346	-0.429	0.354
NKS	23	0.538	0.345	-0.448	0.353

Table 2. Statistics of genome assemblies for three *Ormosia* species (size: bp)

Ormosia purpureiflora				*Ormosia emarginata*		*Ormosia semicastrata*	
Contig statistics of initial assembly using Nanopore reads		Final assembly		Final assembly		Final assembly	
Sequence length	Order of the sequence length	Chromosome	Length	Chromosome	Length	Chromosome	Length
N10=122192683	L10=2	chr1	259935025	chr1	199918031	chr1	205218018
N20=120000233	L20=3	chr2	233292245	chr2	210768611	chr2	211883283
N30=75858835	L30=5	chr3	229093642	chr3	183696964	chr3	200464886
N40=61354201	L40=8	chr4	212222348	chr4	180298008	chr4	178099194
N50=50908349	L50=11	chr5	195349128	chr5	202609791	chr5	205007630
N60=45450924	L60=15	chr6	187433795	chr6	149243870	chr6	185806757
N70=36587725	L70=20	chr7	144758916	chr7	145867561	chr7	164432676
N80=15728371	L80=28	chr8	121398155	chr8	147815325	chr8	159254978
N90=3163854	L90=56	Unanchored to chromosome	645,468	Unanchored to chromosome	35,505	Unanchored to chromosome	519,897
N100=34487	L100=313						
Total length	1,811,176,403		1,584,128,722		1,420,253,666		1,510,687,319
Average length	5,786,506.08		144,011,702		142,025,366.60		151,068,731.90
Largest length	142,757,542		259,935,025		210,768,611		211,883,283
Minimum length	34,487		205,949		1,099		259,325

Figure legends

Figure 1 Picture showing *Ormosia purpureiflora*. A) *O. purpureiflora* seeds. Seed sizes measured by a ruler are illustrated by a small picture embedded in the lower right and chromosome number observation results embedded in the lower left (scale bar: 10 μm); B) *O. purpureiflora* flowers; C) *O. purpureiflora* fruit in the distance showing attack by insect/disease; D) *O. purpureiflora* seeds attacked by a worm and insect/disease; E) *O. purpureiflora* flowers attacked by insect/disease; F) *O. purpureiflora* growing habitat and an arrow showing the sampled individual (a small shrub) used for genome assembly.

Figure 2 Sampled (sub)populations and population genetics of *Ormosia purpureiflora*. A) Map showing (sub)populations sampled for *Ormosia purpureiflora*; B) PCA results showing the first three components (PC1 vs. PC2 and PC1 vs. PC3) for *O. purpureiflora* individuals sampled from different (sub)populations; C) Admixture results representing data for K=2–4 clusters; D) Genetic diversity values (*pi*, *dxy*, *Ho*, *He* and *Fst*) along the *O. purpureiflora* chromosomes (per 1 Mbp).

Figure 3 *Ormosia* genomes and comparative genomics. A) Hi-C interaction heat maps (bin length 100,000 bp) for the three *Ormosia* genome assemblies. B) Circos plot showing the genome features (chromosome, repeat density, gene density and syntenic blocks from outer to inner) across the chromosomes of three *Ormosia* genome assemblies. In *O. purpureiflora*, its circos also includes SNP density results between the results of gene density and syntenic blocks. All densities were estimated with a 1-Mbp sliding window; C) The inferred phylogenetic tree, divergence time, and contracted (–) and expanded (+) gene families in *O. purpureiflora* and other species. D) The density distribution of synonymous nucleotide substitutions (*Ks*) in whole genome duplication analysis for *Ormosia* species and their sister species of *Lupinus albus*; E) Syntenic blocks among *Ormosia* species and *L. albus*; F) Intra-chromosomal structural variation detection among three *Ormosia* species.

Figure 4 Smoothing lines for gene and repeat density distributions (Bin size: 100,000 bp) along chromosomes in *Ormosia* species. Scatterplots for the gene density and repeat distribution are presented in Supplementary Figure S2. Box sizes correspond to chromosome sizes in *Ormosia* species and the black bar on the upper part of each box (chromosome) present the hot structural rearrangement region in the chromosome.

Additional files

Supplementary Table S1 Protein sequences of species used for gene prediction.

Supplementary Table S2 Species used for comparative genomics.

Supplementary Table S3 Species pairs and their estimated divergence times used for time calibration points to infer time-calibrated phylogeny of *Ormosia purpureiflora*.

Supplementary Table S4 Repeat contents in *Ormosia* assemblies.

Supplementary Table S5 Summary of gene functional annotations of the *Ormosia purpureiflora* assembly using different databases.

Supplementary Table S6 GO enrichment results for possible Helitron mediated genes in *Ormosia purpureiflora.*

Supplementary Table S7 KEGG enrichment results for possible Helitron mediated genes in *Ormosia purpureiflora.*

Supplementary Table S8 GO enrichment results for possible TIR mediated genes in *Ormosia purpureiflora.*

Supplementary Table S9 KEGG enrichment results for possible TIR mediated genes in *Ormosia purpureiflora.*

Supplementary Table S10 Comparation of gene number in part of InterPro accesssion among three *Ormosia* species.

Supplementary Table S11 Summary of gene families.

Supplementary Table S12 GO enrichment results for *Ormosia purpureiflora* specific gene families.

Supplementary Table S13 KEGG enrichment results for *Ormosia purpureiflora* specific gene families.

Supplementary Table S14 GO enrichment results for *Ormosia purpureiflora* significantly expanded gene families.

Supplementary Table S15 KEGG enrichment results for *Ormosia purpureiflora* significantly expanded gene families.

Supplementary Table S16 GO enrichment results for *Ormosia purpureiflora* siginficantly contracted gene families.

Supplementary Table S17 KEGG enrichment results for *Ormosia purpureiflora* significantly contracted gene families.

Supplementary Table S18 Number of different gene duplication in *Ormosia*

Supplementary Table S19 GO enrichment results for *Ormosia purpureiflora* WGD genes.

Supplementary Table S20 KEGG enrichment results for *Ormosia purpureiflora* WGD genes.

Supplementary Table S21 GO enrichment results for *Ormosia purpureiflora* TD genes.

Supplementary Table S22 KEGG enrichment results for *Ormosia purpureiflora* TD genes.

Supplementary Table S23 GO enrichment results for *Ormosia purpureiflora* PD genes.

Supplementary Table S24 KEGG enrichment results for *Ormosia purpureiflora* PD genes.

Supplementary Table S25 Results of the syntenic block analysis of *Ormosia* species.

Supplementary Table S26 Structural variation between *Ormosia* species.

Supplementary Table S27 GO enrichment results for *Ormosia purpureiflora* specific inversion on chromosome 2.

Supplementary Table S28 GO enrichment results for possible *Copia* mediated genes in *Ormosia purpureiflora.*

Supplementary Table S29 KEGG enrichment results for possible *Copia* mediated genes in *Ormosia purpureiflora.*

Supplementary Table S30 Highly and lowly expressed genes estimated by nf-differenceabundance in leaf tissue.

Supplementary Table S31 GO enrichment results for *Ormosia purpureiflora* highly expressed genes in leaf tissue.

Supplementary Table S32 KEGG enrichment results for *Ormosia purpureiflora* highly expressed genes in leaf tissue.

Supplementary Table S33 GO enrichment results for *Ormosia purpureiflora* lowly expressed genes in leaf tissue.

Supplementary Table S34 KEGG enrichment results for *Ormosia purpureiflora* lowly expressed genesin leaf tissue.

Supplementary Table S35 Highly and lowly expressed genes estimated by nf-differenceabundance in flower tissue.

Supplementary Table S36 GO enrichment results for *Ormosia purpureiflora* highly expressed genes in flower tissue.

Supplementary Table S37 KEGG enrichment results for *Ormosia purpureiflora* highly expressed genes in flower tissue.

Supplementary Table S38 GO enrichment results for *Ormosia purpureiflora* lowly expressed genes in flower tissue.

Supplementary Table S39 KEGG enrichment results for *Ormosia purpureiflora* lowly expressed genesin flower tissue.

Supplementary Table S40 Highly and lowly expressed genes estimated by nf-differenceabundance in fruit tissue.

Supplementary Table S41 GO enrichment results for *Ormosia purpureiflora* highly expressed genes in fruit tissue.

Supplementary Table S42 KEGG enrichment results for *Ormosia purpureiflora* highly expressed genes in fruit tissue.

Supplementary Table S43 GO enrichment results for *Ormosia purpureiflora* lowly expressed genes in fruit tissue.

Supplementary Table S44 KEGG enrichment results for *Ormosia purpureiflora* lowly expressed genes in fruit tissue.

Supplementary Table S45 Highly and lowly expressed genes estimated by nf-differenceabundance in seed tissue.

Supplementary Table S46 GO enrichment results for *Ormosia purpureiflora* highly expressed

genes in seed tissue.

Supplementary Table S47 KEGG enrichment results for *Ormosia purpureiflora* highly expressed genes in seed tissue.

Supplementary Table S48 GO enrichment results for *Ormosia purpureiflora* lowly expressed genes in seed tissue.

Supplementary Table S49 KEGG enrichment results for *Ormosia purpureiflora* lowly expressed genes in fruit tissue.

Supplementary Table S50 The *R* genes in the species.

Supplementary Table S51 Number of highly and lowly expressed *R* genes and their percentages in the total highly and lowly expressed genes in different *Ormosia purpureiflora* tissues.

Supplementary Table S52 Summary of transcription factor in *Ormosia* and the other compared species.

Supplementary Table S53 Number of highly and lowly expressed TF genes and their percentages in the total highly and lowly expressed genes in different *Ormosia purpureiflora* tissues.

Supplementary Table S54 Highly and lowly expressed TF genes in *Ormosia purpureiflora* leaf tissues.

Supplementary Table S55 Highly and lowly expressed TF genes in *Ormosia purpureiflora* flower tissues.

Supplementary Table S56 Highly and lowly expressed TF genes in *Ormosia purpureiflora* fruit tissues.

Supplementary Table S57 Highly and lowly expressed TF genes in *Ormosia purpureiflora* seed tissues.

Supplementary Table S58 KEGG enrichment results for *Ormosia purpureiflora* candidate adaptive genes.

Supplementary Table S59 KEGG enrichment results for *Ormosia purpureiflora* genes in chr5 region presumably under balancing selection.

Supplementary Figure S1 Genome size estimation by GenomeScope.

Supplementary Figure S2 Gene and repeat density (Bin size: 100,000 bp) along chromosomes

in *Ormosia* species. The smoothing line was added for each density distribution by the geom_smooth() function in the ggplot2 program of the R package. Box sizes corresponding to chromosome sizes in *Ormosia* species.

Supplementary Figure S3 The plant–pathogen interaction pathway map generated using KAAS https://www.genome.jp/tools/kaas/. The green boxes represent genes identified in the *Ormosia* genome assemblies. The red arrows show that the EDS1 gene is missing in *O. purpureiflora* but identified in the other two *Ormosia* species.

Supplementary Figure S4 Dot plots among three *Ormosia* species. The red arrow indicates a specific inversion on chromosome 2 of *O. purpureiflora*.

Supplementary Figure S5 Venn diagrams showing tissue-specific and commonly expressed genes.

Supplementary Figure S6 Distribution of *R* genes on each chromosome in three *Ormosia* species.

Supplementary Figure S7 Scree plot from PCAdapt.

Supplementary Figure S8 Cross validation (CV) error plot of admixture analysis.

Supplementary Figure S9 Heatmap showing the relatedness of individuals in each *Ormosia purpureiflora* (sub)population.

Supplementary Figure S10 Spatial correlograms of relatedness coefficients for different *Ormosia purpureiflora* (sub)population. The confidence interval (95%) for the average relatedness coefficient in a particular distance class was obtained from 999 permutations of individual spatial locations. Relatedness coefficients above or below the 95% confidence envelopes (red dashed lines) indicate a degree of genetic structuring that is significantly larger or smaller than expected at random.

Figure 1

Click here to access/download;Figure;Figure_1.jpg

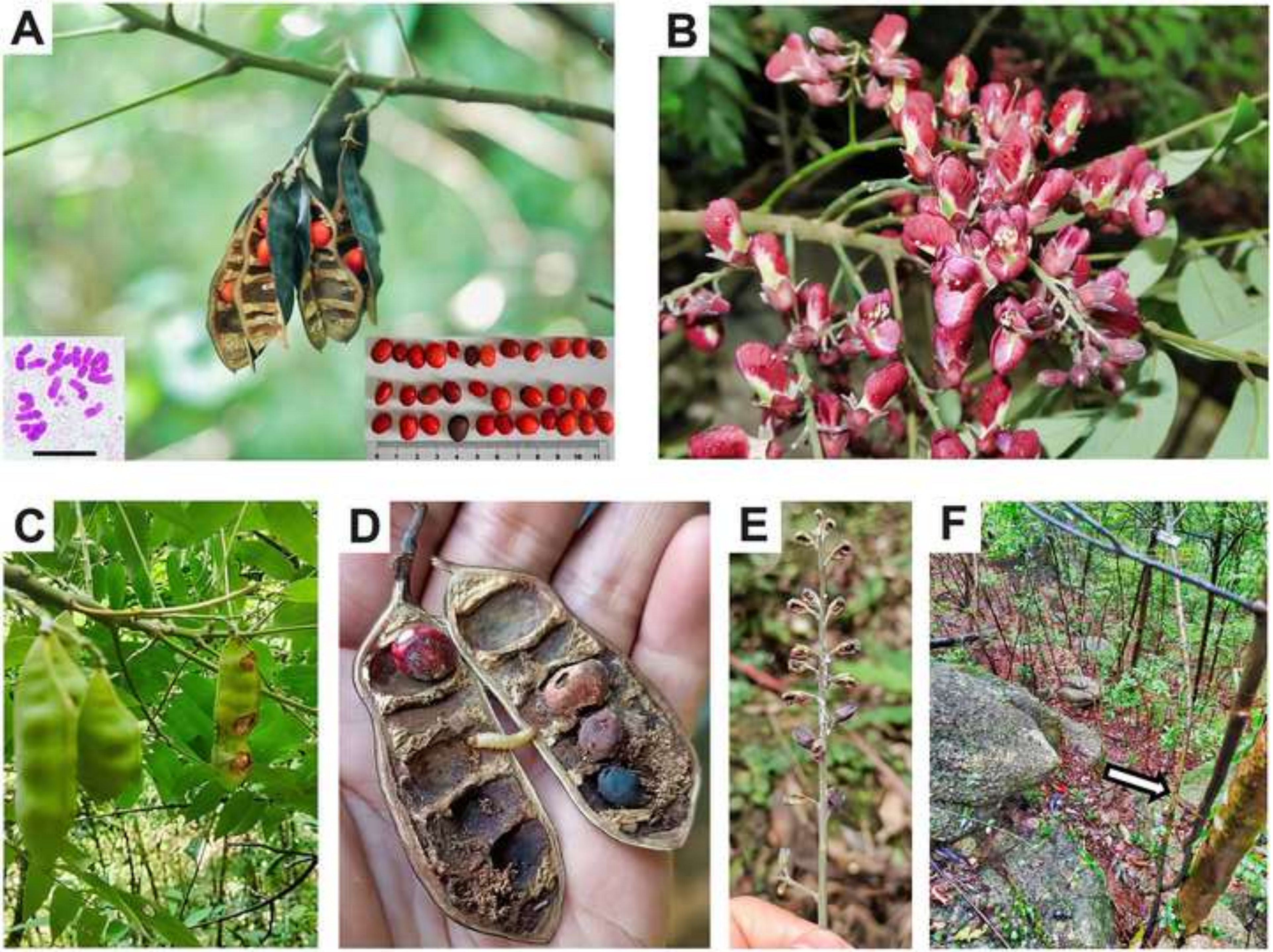

Figure 2

Click here to access/download;Figure;Figure_2.jpg

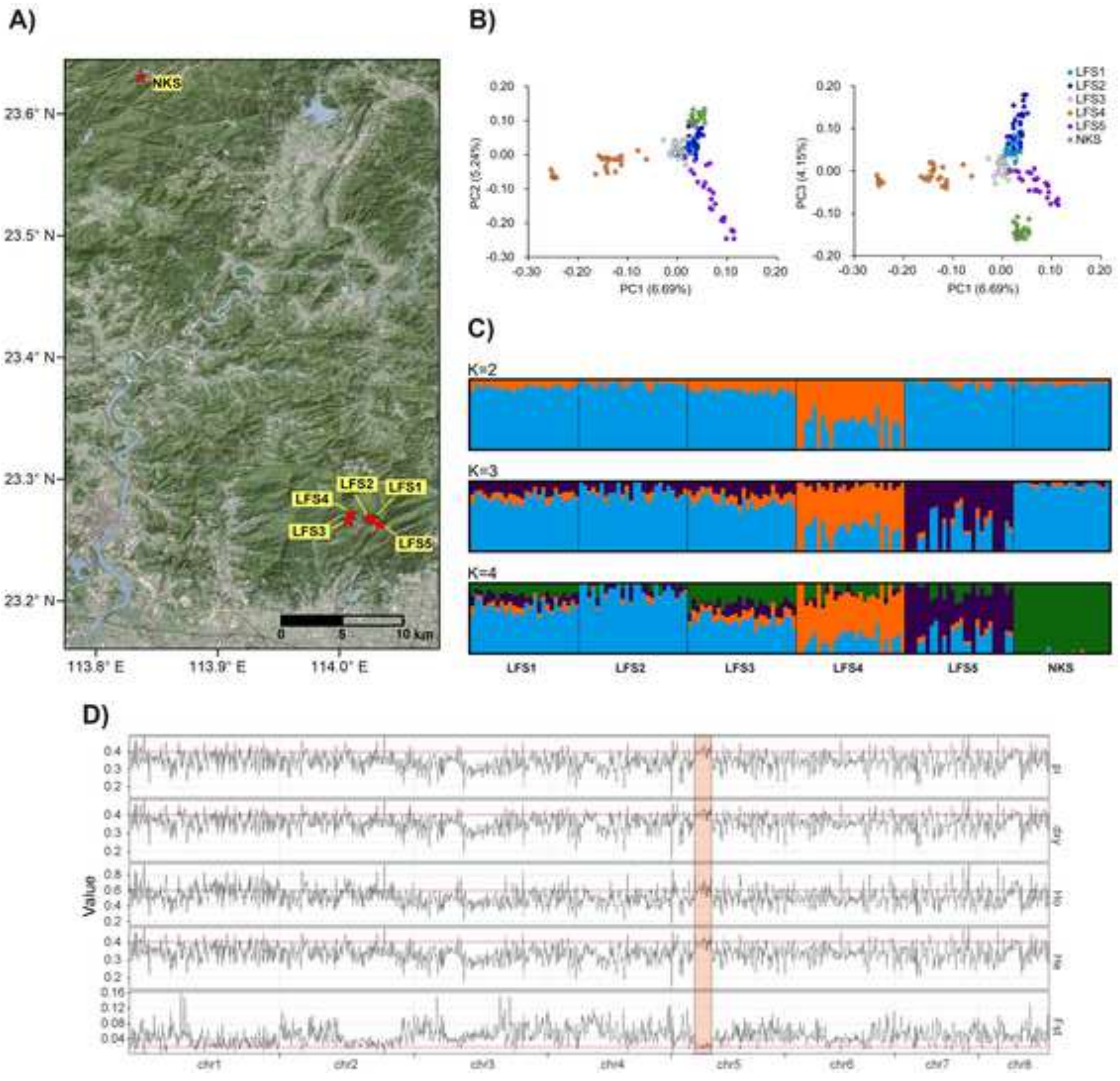

Figure 3

Click here to access/download;Figure;Figure_3.jpg

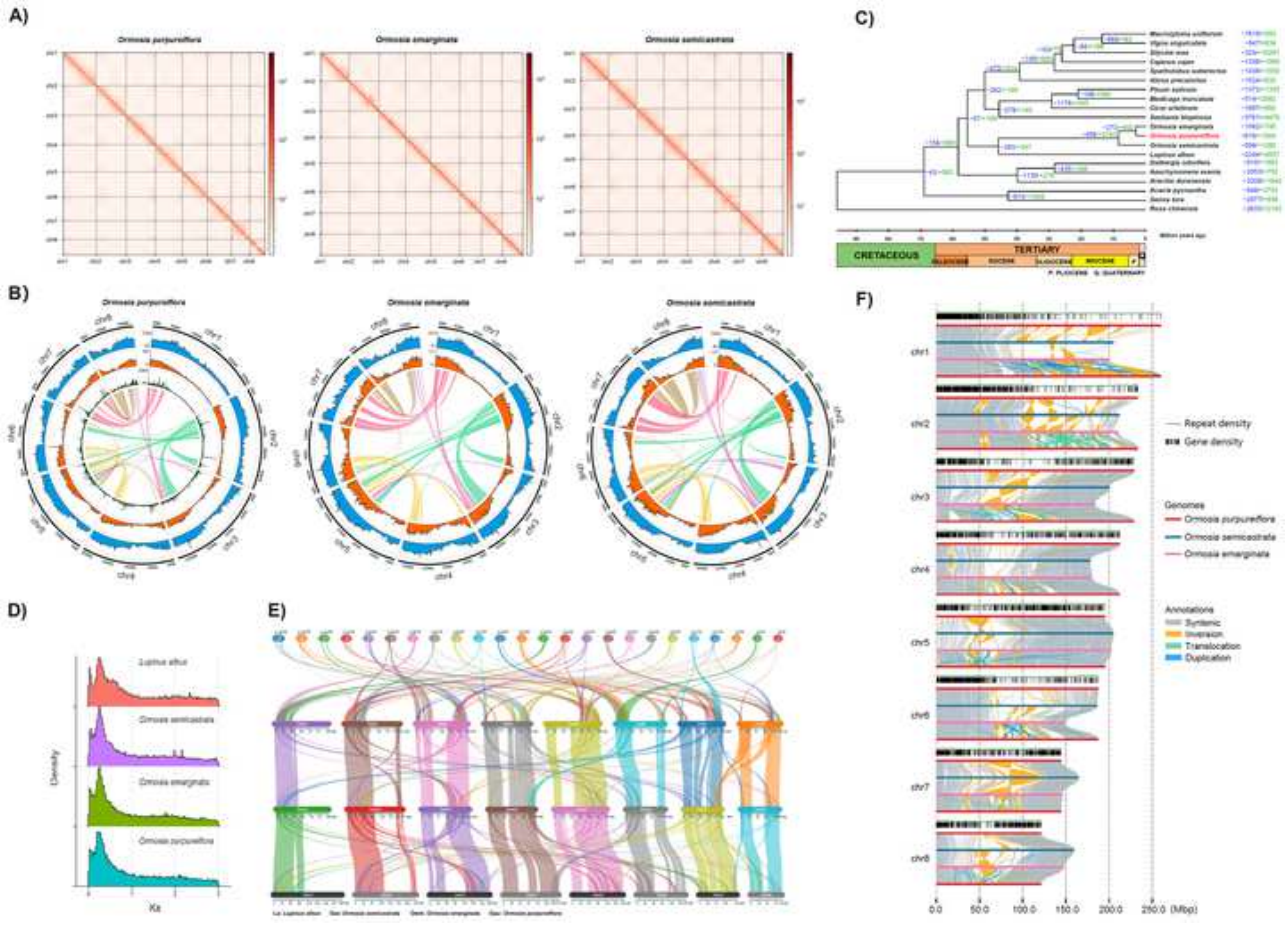

Figure 4

Click here to access/download;Figure;Figure_4.jpg

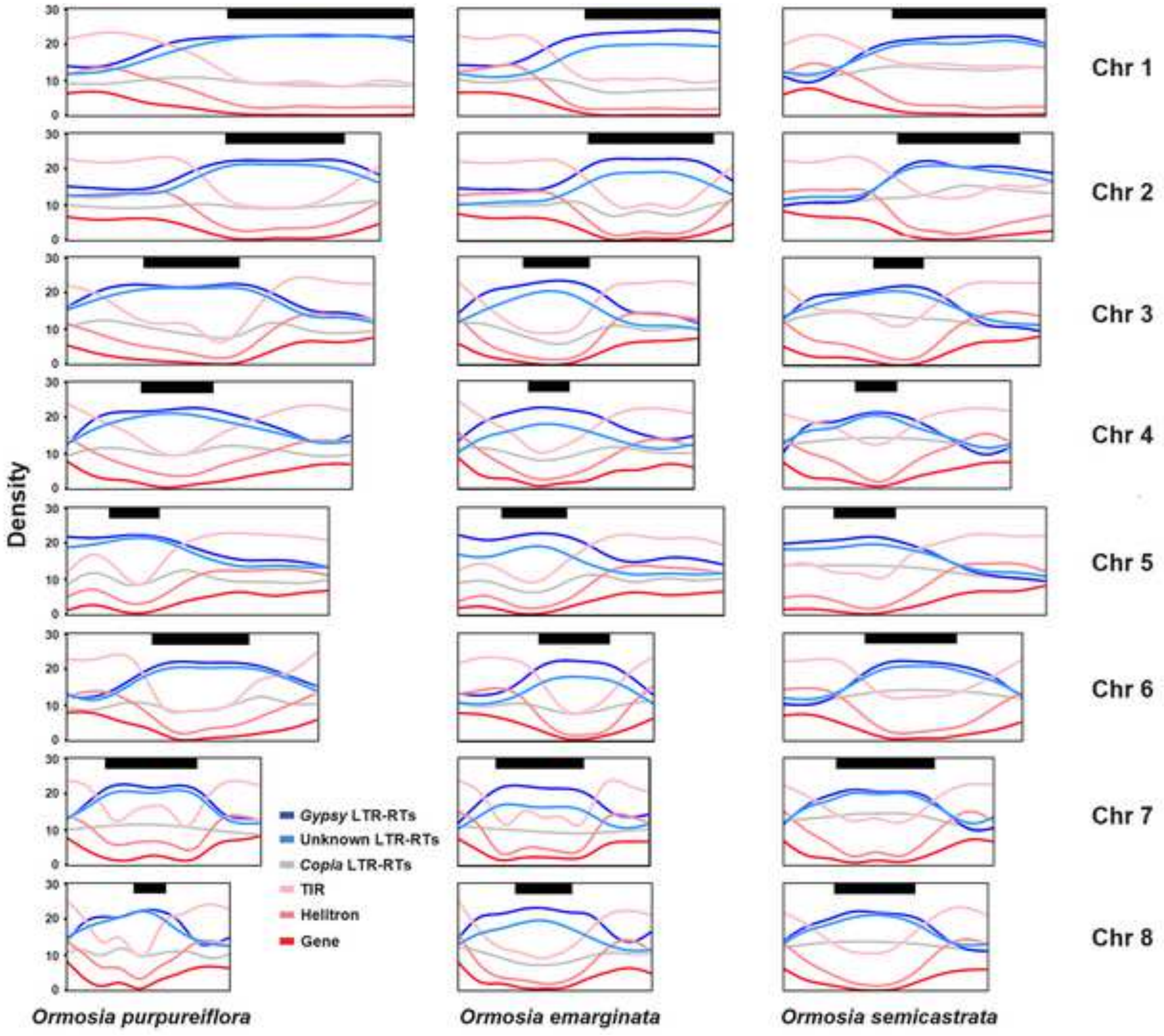

Supplementary Material_Figure_S1

Click here to access/download
Supplementary Material
Figure_S1.png

Supplementary Material_Figure_S2

Click here to access/download
Supplementary Material
Figure_S2.tif

Supplementary Material_Figure_S3

Click here to access/download
Supplementary Material
Figure_S3.tif

Supplementary Material_Figure_S4

Click here to access/download
Supplementary Material
Figure_S4.tif

Supplementary Material_Figure_S5

Click here to access/download
Supplementary Material
Figure_S5.tif

Supplementary Material_Figure_S6

Click here to access/download
Supplementary Material
Figure_S6.tif

Supplementary Material_Figure_S7

Click here to access/download
Supplementary Material
Figure_S7.tif

Supplementary Material_Figure_S8

Click here to access/download
Supplementary Material
Figure_S8.tif

Supplementary Material_Figure_S9

Click here to access/download
Supplementary Material
Figure_S9.tif

Supplementary Material_Figure_S10

Click here to access/download

Supplementary Material

Figure_S10.tif

Click here to access/download
Supplementary Material
supplymentary_tableS1-S59.xlsx
