# Supplementary material for: Chromosome-scale assemblies of three Ormosia species: repetitive sequences distribution and structural rearrangement
Source: Gigascience. 2025 May 16;14:giaf047. doi: 10.1093/gigascience/giaf047 (PMC12083454; doi:10.1093/gigascience/giaf047)
Supplement: giaf047_GIGA-D-24-00350_Revision_1 [file giaf047_giga-d-24-00350_revision_1.pdf]

GigaScience

Chromosome-scale assemblies of three Ormosia species: Gene-repeat association architecture, structural rearrangement and balancing selection

--Manuscript Draft--

**Manuscript Number:**	GIGA-D-24-00350R1
**Full Title:**	Chromosome-scale assemblies of three Ormosia species: Gene-repeat association architecture, structural rearrangement and balancing selection
**Article Type:**	Data Note
**Funding Information:**	
**Abstract:**	Background:<br>The genus Ormosia is in Fabaceae family. China is one of its centers with almost all of its species endemic, suggesting need for genomic studies to understand its evolution and help its conservation and usage. Therefore, the chromosome-scale assembly of O. purpureiflora was performed, and that for O. emarginata and O. semicastrata were updated.<br><br>Findings:<br>The genome assembly sizes of the three species varied from 1.42 to 1.58 Gb, and O. purpureiflora was the largest. Repeats accounted for 74.0–76.3% of sequences in the assemblies; the predicted genes varied from 50,517 to 55,061.<br>Repeats contributed largely to the Ormosia chromosome architectures, in which Helitron and Terminal Inverted Repeat (TIR) were associated with gene distribution, while Gypsy and unknown LTR were related to structural rearrangements.<br>Ormosia contained substantial resistance (R) genes but fewer transcription factor genes. Alkaloid-, terpene- and flavonoid-related genes were found with tandem or proximal duplications. Some growth- and defense-related genes were missed in O. purpureiflora.<br>By resequencing 153 genotypes (~30 Gb data each) in 6 O. purpureiflora (sub)populations, 276,854 high-quality single nucleotide polymorphisms (SNPs) were identified, in which 2632 were presumably adaptive SNPs, and their function was mainly related to carbohydrate metabolism. High genetic diversity in O. purpureiflora was revealed even though it had very small populations. A weak and absent spatial genetic structures indicating high pollen flows within/among the (sub)populations. An approximately 30-Mb region on chromosome 5 was found to likely be under balancing selection.<br><br>Conclusions:<br>The Ormosia assemblies provide valuable resources for evolutionary study, conservation and utility in both Ormosia and Fabaceae.
**Corresponding Author:**	Zheng-Feng Wang<br>South China Botanical Garden, Chinese Academy of Sciences<br>CHINA
**Corresponding Author Secondary Information:**	
**Corresponding Author's Institution:**	South China Botanical Garden, Chinese Academy of Sciences
**Corresponding Author's Secondary Institution:**	
**First Author:**	Zheng-Feng Wang
**First Author Secondary Information:**	
**Order of Authors:**	Zheng-Feng Wang
	En-Ping Yu
	Lin Fu
	Hua-Ge Deng

Powered by Editorial Manager® and ProduXion Manager® from Aries Systems Corporation

	Wei-Guang Zhu
	Feng-Xia Xu
	Hong-Lin Cao
**Order of Authors Secondary Information:**	
**Response to Reviewers:**	GigaScience<br>December 12, 2024<br><br>Dear editor,<br>Thank you very much for your decision letter about our manuscript entitled “Chromosome-scale assemblies of three Ormosia species: Gene-repeat association architecture, structural rearrangement, and balancing selection” (No. GIGA-D-24-00350), including the comments.<br>We are now sending our responses to comments with our revised manuscript (both tracked and clean versions). All the modified parts in the revised manuscript are marked in red.<br>Our specific responses are as follows:<br><br>Comments from the reviewers:<br>Reviewer #1:<br>1. There is very little context about important information of the sequenced species such as the ploidy and the reproductive mode (sexual reproduction, autogamous or allogamous, etc). This information is critical to know the expected assembly that should be pursued. The analysis of the genomic variation dataset should include an analysis of heterozygosity per sample to know which type of assembly should be generated. In case that a phased assembly would be appropriate, I assume that this could not be made due to the error rate of nanopore reads. This error rate should be reported and the reason for not trying a phased assembly must be clearly stated in the manuscript.<br>>>>1) We have included information on the ploidy and reproductive mode, as suggested. Please refer to lines 87–90, 187–188, 372–376, and Table S4.<br>2) We have included information on the heterozygosity for Ormosia purpureiflora and the other two Ormosia species. Please refer to lines 185–187, 390–391, and 397–399.<br>3) We have incorporated the error rate estimation for Nanopore reads and additional details addressing the phased assembly. Please refer to lines 146–147 and 431–439.<br><br>2. The information related to the origin of the RNA-seq data is unclear. The text seems to indicate that RNA-seq was collected from 153 accessions, but then the SRA records suggest that only one accession was sequenced. More importantly, the experimental design did not seem to include biological replicates. This makes the results of differential expression meaningless. Taking into account that the manuscript is a data note, I recommend to completely remove the section "RNA-seq analysis of tissues". The RNA-seq data generated in this work is worth to aid the gene annotation process, but the experiment is not correctly designed to assess differential expression. As a small note, nothing is "highly significant". Significance is an assessment of probability that an event (a difference in many cases) is real. Hence, it should not be a term subject to gradation.<br>>>>We performed RNA-seq of only one O. purpureiflora individual, which was used for genome annotation. The other 153 samples underwent only whole-genome resequencing and were not subjected to RNA-seq. We have revised the text to clarify this point. Please refer to lines 153–155. In addition, we have removed references to “RNA-seq analysis of tissues” throughout the text and corrected the incorrect use of the term “highly significant,” as suggested.<br><br>3. Likewise, the protocol used to obtain DNA sequencing data for the diversity panel is unclear. Regarding the samples, it is not clear if the sampling is representative for the species. Regarding the data, it is not clear which protocol was used for sequencing. The text suggests that it was WGS, but the number of reported SNPs is very low for a WGS dataset of a plant species. The methods indicate that SNPs were called with a pipeline called "dDocent". Looking at the documentation, this seems to be a pipeline for RAD-sequencing data, which probably means that it is not a good option

for WGS data. This could be the main reason explaining why the number of SNPs is very small for a WGS dataset. This could also explain the reported result regarding excess of heterozygosity. If the authors want to release the resequencing data an a database of genomic variation, I recommend using more standard variant calling and genotyping tools such as NGSEP or GATK and redo the downstream analysis using the possibly more than 1 million SNPs that should be obtained from these data.

>>>1) We have clarified the sampling information in the text; please refer to lines 84–87 and 148–151. The sampling strategy was based on a thorough field investigation. Each (sub)population was randomly sampled, ensuring that sampling areas covered the entire region of each (sub)population. Sampling sizes ranged from 23 to 26 individuals per (sub)population, which are appropriate for population genetics studies.

2) The SNP-calling pipeline, dDocent (which uses Freebayes for SNP calling), is compatible with both RAD-seq and whole-genome resequencing data (please see https://www.ddocent.com/). We have clarified this point in the text; please refer to lines 311–318. Similarly, NGSEP can handle various NGS data types, including RAD-seq data (https://github.com/NGSEP/NGSEPcore).

We conducted whole-genome resequencing for each sample, and the initial SNP calling using Freebayes in the dDocent pipeline yielded 238,531,000 SNPs. However, only a small proportion of SNPs remained after filtering for low quality, Hardy-Weinberg equilibrium (HWE), and other parameters. Filtering out low-quality SNPs ensures accurate genetic diversity and structure analyses.

Additionally, we included NGSEP and GATK results in our study. However, due to the significant computational time required for GATK, we used it only to validate dDocent and NGSEP results, rather than for whole-genome SNP calling. Genetic diversity and structure analyses were then conducted using SNPs identified by all three tools. We have added a detailed explanation and schematic of this process in Supplementary Figure S1 and the “Single-nucleotide polymorphism calling” section in “Methods”. Accordingly, we re-analyzed the SNP results. Please see sections of “Single-nucleotide polymorphism calling” and “Genetic diversity and genetic structure” in “Results”.

3) The low number of SNPs does not affect the observed excess heterozygosity. The retained SNPs adequately reflect population genetic processes because they are neutral and randomly distributed across chromosomes. In our current research on another species (Dunnia sinensis, unpublished), we identified 144,746 SNPs (including 112,324 bi-allelic SNPs) after filtering. Using these SNPs, we found two populations with heterozygosity deficits (inbreeding coefficient Fis values of 0.9889 and 0.9895), whereas the other four populations exhibited excess heterozygosity (Fis values ranged from −0.6475 to −0.7100).

In O. purpureiflora, excess heterozygosity may be explained by outcrossing and clonal reproduction. We have included an explanation for this in the text; please refer to lines 659–669.

4. About the analysis of genetic structure, if the number of sampled populations is actually 6, it does not make sense to show the results only until k=4. The resolution of figure 2 needs to be improved. I can not differentiate correctly the populations in the current PCA. If the most genetically distant population is NKS, it is weird that this population gets differentiated only until k=4. Again k=5 and k=6 are needed to evaluate the stability of this differentiation.

>>>After submission, the journal reduced the resolution of the figures to minimize the manuscript size. However, high-resolution figures can be accessed by clicking on and downloading them for review.

Additionally, we have included the result for K = 6 and revised the text accordingly. Please refer to Figure 2C and lines 686–690.

Minor comments.

1. The order of the figures looks disorganized relative to the order of the text. The reason for this seems to be that the map in figure 2A is cited in the context to talk about the distribution of the species. Given that this map does not actually show much of global context, I would recommend eliminating the references to figure 2A in the context and in the methods and reorganize the figures.

>>>We have deleted the citation of Figure 2A in the Introduction, as suggested.

2. The resolution of the figures 2 and 3 is very poor. This makes it difficult to follow

Powered by Editorial Manager® and ProduXion Manager® from Aries Systems Corporation

the results included in these figures.

>>>After submission, the journal reduced the resolution of the figures to minimize the manuscript size. However, high-resolution figures can be accessed by clicking on and downloading them for review.

3. Abstract: Please rephrase the second sentence of the background

>>>We have rephrased the texts in the background, as suggested. Please refer to lines 32–34.

4. Line 40: it is not clear what "substantial" means. It is also not clear to what Ormosia is compared in the expression "...fewer transcription factor genes"

>>>We have rephrased the statement for clarity. Please refer to lines 45 – 46.

5. Line 135. Replace "inputed into" with "given to"

>>>We have replaced the term "inputed into" and rephrased the sentence. Please refer to lines 186.

6. Lines 331 - 342. The text looks speculative and I think it is not needed for a data note.

>>>We have removed these sentences as suggested.

7. Lines 352-363. Again, I think that a data note does not require speculation about possible consequences of the genes that look missing in the assemblies.

>>>We have removed these sentences as suggested.

8. Line 392. The expression "a recent ancient WGD" looks self contradictory. More importantly, the event does not seem to be genus specific, but it just seem to be the known event in fabales. The authors could simply refer to this event.

>>>We have corrected these contradictory sentences. Please refer to line 543. For WGD, we have included the required information as suggested. Please refer to lines 544–546.

9. Line 399. It is not clear what are "TD-type genes". I assume they would be the tandem duplication, but this needs to be clear in the text.

>>>We have rephrased the words for better clarity. Please see line 553.

10. Line 401. Likewise, it is not clear what are "PD-type genes".

>>>We have rephrased the words for better clarity. Please see line 555.

11. Line 541-548. Again the text looks speculative. I recommend to remove this paragraph.

>>>We have removed these sentences, as suggested.

12. Lines 550-561. Again I think that this analysis should not be included in a data note, especially because the results do not seem to be conclusive.

>>>We have removed these sentences, as suggested.

Reviewer #2:

General comment: The current state of written English in the manuscript requires attention. A comprehensive review of the English language including sentence structure and overall flow is highly recommended to enhance the quality of the manuscript.

>>>We have used the editing service of an English language editing company, TopEdit (www.topeditsci.com), to improve quality of our manuscript. The English revised parts are marked in red in the text.

Line 40: This is not a very informative sentence to highlight the main findings. "substantial" and "fewer" are relative terms, thus substantial R genes and fewer TFs does not reveal the true extent/number of these identified genomic regions. please reframe.

>>>We have rephrased the sentences. Please refer to lines 45–46.

Line 42: what does it mean by "missed"? missed in the analysis or absent in the species?

>>> We have replaced “missed” with “absent.” Please refer to line 48.

It would be helpful to provide an account of the current status of genomic studies in the genus and may be closely related species.
>>>We have included information on the genomic studies conducted in the genus so far. Please refer to lines 101–112.

Line 102: Is the storage temperature for root tips indicated here, correct? Were the root tips incubated at subzero temperature?
>>>We have rephrased the sentences for clarity. Please refer to lines 126–128.

Figure 1A: Karyotype picture its loosing its relevance in the inset. Please consider reordering it.
>>>We have redrawn the picture and included the karyotype result as a separate supplementary file, Figure S2.

Lines 108-127: The authors have not provided details regarding the preparation of the DNA/RNA samples and sequencing libraries, which is a critical omission. Given that the study focuses on a less-studied plant species that is not well-characterized, this information is especially important for ensuring the reproducibility of the results and aiding future studies. I highly recommend including a clear description of the library preparation methods.
>>>The details of sequencing information, including DNA/RNA preparation and library construction (except Nanopore ultra-long sequencing), have been provided in our previous studies. We have cited our previous studies and added details for Nanopore ultra-long sequencing. Please refer to lines 162–176.

It is unclear whether biological replicates were employed in the RNA sequencing. Please include these details.
>>>We did not use replicates and thus have removed RNA expression analysis as suggested by the other reviewer.

Line 129-138: Although, authors have reported the followed pipeline and softwares used for read processing and genome assembly, it is important to include details on the parameters used for each employed software. This is universally applicable to all the analysis softwares mentioned throughout the different portions of the study. These parameters should be fully described.
>>>We have used the default setting for programs during analyses. For additional parameters, we have described the detailed information in the revised manuscript. Please refer to lines 177–178.

Line 166-167: It is not clear how the redundancy in repeats identified through two different programs, EDTA and RED were taken care of.
>>>In combination, both overlapped and nonoverlapped repetitive regions of EDTA and RED were merged to form overall repetitive regions in the assembly. We have revised the information for better clarity. Please refer to lines 226–230.

Line 219-229: Identification of R genes has not been described.
>>>We have included the description in the text. Please refer to lines 295–300.

Line 291-293: I was expecting to see some information about how application of HiC has improved the previous reported assemblies of O. emarginata and O. semicastrata.
>>>We have included this information as suggested. Please refer to lines 396–397, 400–404, and Table 2.

Line 294-299: BUSCO indicates completeness of genic regions of the assemblies which are often not the most difficult genome regions to map and assemble. Authors should evaluate the integrity and completeness of the assembly through other metrics. My suggestions would be performing analysis of LTR assembly index (https://academic.oup.com/nar/article/46/21/e126/5068908?login=false), mapping back the short and long-read DNA/RNA sequences onto to assembly to correlate mapping percentages with genome continuity.
>>>As suggested, we have included more quality evaluations in the assemblies

Powered by Editorial Manager® and ProduXion Manager® from Aries Systems Corporation

and replaced Table 2. Please refer to lines 211–216, 413–421, and Table 2.

Line 312-316: What is the length distribution of the genes predicted in the analysis. Have an exploratory analysis done to ascertain the quality of the predicted genes? How does the authors justify only 70% of their predicted genes being annotated.

>>>1) We have updated Table 3 to include the length distribution information. Please refer to Table 3, lines 455-457.

2) To assess gene quality in our studied species, we have included analyses using BUSCO, OMArk, and Fabaceae-represented gene matches. Please see lines 244–254 and 468–494.

3) We compared our annotation rates with those of the other genomes. Annotation rates vary across species, with similarly low rates observed in some cases. This could be attributed to the presence of unknown or novel genes. We have incorporated this information in the text; please refer to lines 460–467.

Line 327: "By also removing possible LTR-mediated genes and reciprocally excluding Helitron- or TIR-mediated genes, we finally retained 7216 possible Helitron-mediated genes and 2728 possible TIR-mediated genes." Does the author means LTRs were nested in the helitron and TIR repeats? and by removing these putative regions 7216 genes were retained from 21641? This statement is not clear.

>>>We have removed the whole paragraph as suggested by the other reviewer.

Line 361: This statement sounds very speculative. have the authors done any validation to make sure these genes are entirely missing from the assembly or have left undetected by the used pipeline?

>>>We have removed the whole paragraph as suggested by the other reviewer. These genes were not annotated in O. purpureiflora when gene annotation was performed using the InterPro database.

Table 2: Authors should consider elaborating the annotation details for the assemblies in the main Table which bring more visibility to the reader.

>>>We have replaced Table 2 and included this information as suggested. Please see Table 2 and Table 3, Table S6.

The manuscript does not describe and provide information about data validation and quality control.

>>>We have included this information as suggested, including additional information on experiments, genome assembly, genome and predicted gene quality evaluations, and SNP quality control. Please see above responses from two reviewers. In addition, all raw sequencing data are available in GenBank (please see “Data Availability”) and the results are available in Figshare (https://doi.org/10.6084/m9.figshare.26826466.v14). The results of synteny and the other comparative genomics among three Ormosia species confirmed their sequencing data are reliable.

Miscellaneous:

1) Because there are references added and removed, the order of references in the revised manuscript are changed. Please see revised citing numbers corresponding to the reference list in the text and “References” section.

2) Because revision, we also remove and replace the tables/figures in the text and supplementary materials.

3) We reanalyze some of the results as suggested, and the results are revised correspondingly.

Please let us know if there are any additional concerns about our manuscript after we have offered these corrections and responses, as we are happy to address any continued issues or proposed changes to the manuscript. Thank you for your time.

Sincerely yours,

Zheng-Feng Wang
South China Botanical Garden
Chinese Academy of Sciences

	Guangzhou, 510650<br>China
**Additional Information:**	
**Question**	**Response**
Are you submitting this manuscript to a special series or article collection?	No
**Experimental design and statistics**<br><br>Full details of the experimental design and statistical methods used should be given in the Methods section, as detailed in our Minimum Standards Reporting Checklist. Information essential to interpreting the data presented should be made available in the figure legends.<br><br>Have you included all the information requested in your manuscript?	Yes
**Resources**<br><br>A description of all resources used, including antibodies, cell lines, animals and software tools, with enough information to allow them to be uniquely identified, should be included in the Methods section. Authors are strongly encouraged to cite Research Resource Identifiers (RRIDs) for antibodies, model organisms and tools, where possible.<br><br>Have you included the information requested as detailed in our Minimum Standards Reporting Checklist?	Yes
**Availability of data and materials**<br><br>All datasets and code on which the conclusions of the paper rely must be either included in your submission or deposited in publicly available repositories (where available and ethically appropriate), referencing such data using a unique identifier in the references and in the “Availability of Data and Materials”	Yes

section of your manuscript.<br><br>Have you have met the above requirement as detailed in our Minimum Standards Reporting Checklist?	

Powered by Editorial Manager® and ProduXion Manager® from Aries Systems Corporation

Manuscript
Click here to access/download;Manuscript;MS-revised-clean.docx

Date note

Chromosome-scale assemblies of three *Ormosia* species: Gene-repeat association architecture, structural rearrangement, and balancing selection

Zheng-Feng Wang[1, 2, 3, 4], En-Ping Yu[1, 2, 3, 4, 5], Lin Fu[1, 3, 4, 6], Hua-Ge Deng[7], Wei-Guang Zhu[1, 2, 3, 4], Feng-Xia Xu[1, 3, 4, 6], Hong-Lin Cao[1, 2, 3, 4]

[1] Guangdong Provincial Key Laboratory of Applied Botany, South China Botanical Garden, Guangzhou, 510650, China

[2] Key Laboratory of Vegetation Restoration and Management of Degraded Ecosystems, South China Botanical Garden, Chinese Academy of Sciences, Guangzhou 510650, China

[3] Key Laboratory of National Forestry and Grassland Administration on Plant Conservation and Utilization in Southern China, South China Botanical Garden, Chinese Academy of Sciences, Guangzhou 510650, China

[4] South China National Botanical Garden, Guangzhou 510650, China

[5] University of Chinese Academy of Sciences, Beijing 100049, China

[6] Key Laboratory of Plant Resources Conservation and Sustainable Utilization, South China Botanical Garden, Chinese Academy of Sciences, Guangzhou 510650, China

[7] Management Office of Guangdong Luofushan Provincial Nature Reserve, Huizhou, 516133, China

Joint first authors:

Zheng-Feng Wang, En-Ping Yu, Lin Fu

Corresponding authors:

Zheng-Feng Wang (wzf@scib.ac.cn); Hong-Lin Cao (caohl@scib.ac.cn)

Abstract

Background: The genus *Ormosia* belongs to Fabaceae family, with China being one of its primary centers, where almost all species are endemic. Thus, genomic studies on the genus are needed to better understand species evolution and ensure the conservation and utilization of these species. We performed a chromosome-scale assembly of *O. purpureiflora* and updated the chromosome-scale assemblies of *O. emarginata* and *O. semicastrata* for comparative genomics.

Findings: The genome assembly sizes of the three species ranged from 1.42 to 1.58 Gb, with *O. purpureiflora* being the largest. Repetitive sequences accounted for 74.0%–76.3% of the genomes, and the predicted gene counts ranged from 50,517 to 55,061. Benchmarking Universal Single-Copy Orthologs (BUSCO) analysis indicated 97.0%–98.4% genome completeness, whereas the long terminal repeat assembly index values ranged from 13.66 to 17.56, meeting the "Reference genome" quality standard. Gene completeness, assessed using BUSCO and OMArk, ranged from 95.1% to 96.3% and 97.1% to 98.1%, respectively.

Repeats played a significant role in shaping the chromosome architecture of *Ormosia*. Helitron and Terminal Inverted Repeat (TIR) elements were associated with gene distribution, whereas *Gypsy* and unknown LTR were linked to structural rearrangements.

Compared with the sister species *Lupinus albus*, *Ormosia* species had lower numbers and percentages of resistance (*R*) genes and transcription factor genes. Genes related to alkaloid, terpene and flavonoid biosynthesis were found to be duplicated through tandem or proximal duplications. Notably, some genes associated with growth and defense were absent in *O. purpureiflora*.

By resequencing 153 genotypes (~30 Gb of data per sample) from six *O. purpureiflora* (sub)populations, we identified 561,922 single nucleotide polymorphisms (SNPs) , including 2922 potentially adaptive SNPs. Despite having very small populations, *O. purpureiflora* exhibited high genetic diversity. A 30-Mb region on Chromosome 5 was identified as likely being under balancing selection.

Conclusions: The *Ormosia* genome assemblies provide valuable resources for studying the evolution, conservation and potential utility of both *Ormosia* and Fabaceae species.

Keywords: Adaptive loci; Comparative genomics; Gene families; Gene duplication; Genetic diversity; Population genetics; RNA-seq; Repeat-mediated chromosome architectures; SNP calling; Structure variation

Data Description

Context

The genus *Ormosia* Jackson, belonging to Fabaceae family, comprises approximately 130–150 species [1, 2]. These species are trees and shrubs that thrive in warm climate. Fossil records suggest that *Ormosia* species were originally distributed in northern regions of the North Hemisphere and migrated southwards during the Paleogene or Neogene period due to climate cooling [3]. Today, Their distributions spans tropical America, Southeast Asia and North Australia [1–3], following a typical Asian–American Tropical Disjunction Pattern [1]. Continental Asia is widely considered the center of origin for the genus.

One of the most distinctive features of *Ormosia* species is their brightly colored seeds, including red, orange, bicolored red/orange, or black color (Fig. 1A), These seeds are commonly used in ethnic jewelry and other decorative applications [1]. In addition, certain *Ormosia* species have high-value timber, and are cultivated as ornamental landscape trees [4, 5]. Extracts from their seeds, roots, stems, bark and leaves have medicinal applications [6, 7], containing bioactive compounds such as alkaloids, flavonoids, isoflavones, terpenes, and lignans [6–8]. Metabolomic and transcriptomic analyses have further revealed that transcription factors play a key role in regulation of flavonoid and terpenoid biosynthesis in *Ormosia* species [9, 10].

In China, approximately 37 species of *Ormosia* exist, and 34 of them are endemic [2]. *O. purpureiflora* is one such endemic species. Unlike most *Ormosia* species, which typically have white or yellow flowers, *O. purpureiflora* is characterized by its purple flowers (Fig. 1B), as reflected in its species name (*purpureiflora*). This species has been identified at only two locations in southeast China: the Guangdong Longmen Nankunshan (NKS) Provincial Natural Reserve and Guangdong Luofushan (LFS) Provincial Natural Reserve. Field investigations have revealed approximately 2000 individuals across these two sites [11]. A detailed survey of five plots (each 20 m × 20 m, four in LFS and one in NKS) recorded a total of 1,468 individuals. *O. purpureiflora* is a small shrub, with a diameter at breast height not exceeding 5 cm (average: 1.74 cm) and a height of no more than 4.5 m (average: 1.02 m). The species produces bisexual flowers in panicles or racemes and is primarily insect-pollinated, with bees as the main pollinators. Although it exhibits typical outcrossing, self-pollination is also possible, as confirmed by bagging experiments [11]. Root-

derived clonal reproduction has also been observed in the field [11]. The species is subject to severe pest and disease attacks affecting both flowers and fruit (Fig. 1C–E), resulting in low seed yields in its natural habitat.

Advancements of high-throughput sequencing technologies have enabled the assembly of full genome information in diversified species, facilitating their conservation, restoration, management and utilization. In this study, we generated a high-quality genome of *O. purpureiflora* by using a combination of long- and short-read whole genome sequencing (WGS), high-throughput chromosome conformation capture (Hi-C) sequencing, and RNA sequencing (RNA-Seq) of different tissues for annotation. In addition, we examined genetic diversity and conducted population genetics analyses for *O. purpureiflora* by resequencing 153 samples collected from two sites.

Genomic studies in *Ormosia* have primarily focused on its chloroplasts, with approximately 15 species, including *O. purpureiflora*, having been studied [12, 13]. To date, only one mitochondrial genome has been reported, which is from *O. boluoensis* [14]. Moreover, draft nuclear genomes have been reported for only two species, *O. emarginata* and *O. semicastrata* [15]. According to phylogenetic studies in Fabaceae [16], *Ormosia* belongs to the Genistoid lineage, where it is most closely related to *Hovea* and *Poecilanthe*. However, no genomes have been reported for these two genera. Among the Genistoid, genomes from only two *Lupinus* species, specifically *Lupinus albus* [17] and *Lupinus angustifolius* [18], had been published at the time of our manuscript preparation. Presently, additional genomes with well-annotated genes have become available, including those of *L. luteus* [19], *Crotalaria pallida* (GenBank accession: GCA_037953625.1), *Ammopiptanthus mongolicus* [20], and *Sophora flavescens* [21]. We aim to incorporate these newly available genomes in our future studies.

For comparative genomics, we updated the genome assemblies of previously published *O. emarginata* and *O. semicastrata* genomes [15] by using Hi-C data to generate chromosome-scale assemblies. Compared with *O. purpureiflora*, both *O. emarginata* and *O. semicastrata* are widespread species in southern and southeastern China, with *O. emarginata* extending into Vietnam. Unlike the small shrub *O. purpureiflora*, both *O. emarginata* and *O. semicastrata* are large trees. Phylogenetic analyses conducted by Torke et al. [1] placed *O. emarginata* and *O. semicastrata* in

different clades, with *O. emarginata* belonging to the Old World *Ormosia* clade 1 and *O. semicastrata* in the Old World *Ormosia* clade 2. However, *O. purpureiflora* was not included in these phylogenies.

Methods

Chromosome number observation

The individual used for chromosome number observation in *O. purpureiflora* was regenerated from seeds collected at LFS. Its root tips were pretreated with 0.002 M 8-hydroxyquinoline for 6 h and then fixed in a 3 : 1 (v:v) solution of absolute ethanol and glacial acetic acid for 24 h at room temperature. After fixation, the root tips were transferred to 70% ethanol and stored at −4°C until chromosome counts were performed. For chromosome counting, the fixed root tips were hydrolyzed in a 1 : 1 (v:v) solution of 1 M absolute ethanol and hydrochloric acid at room temperature for 7 min, rinsed within water, and then stained with carbol fuchsin for 4 min. Meristems were then excised and squashed for microscope observation. Photographs were taken using an Olympus BX-43 microscope (Olympus Corporation, TN, USA) at 100× magnification with an Olympus DP26 camera (Olympus Corporation, TN, USA).

Sample collection and sequencing

An *O. purpureiflora* individual (Fig. 1F) collected from LFS was used for genome assembly. Genomic DNA was isolated from its leaf tissues and multiple libraries were constructed, including long- and short-read WGS and Hi-C libraries. For gene annotation, RNA was extracted from the same individual used for genome assembly, specifically from its leaves, flowers, seeds and fruit. RNA-seq libraries were then constructed for these tissues. Long-read WGS was performed using the Oxford Nanopore Technologies (ONT) PromethION sequencer (Oxford Nanopore Technologies plc. OX, UK). Both long-read and ultra-long-read (50 kb) sequencing libraries were generated on the ONT platform. Short-read WGS, Hi-C and RNA-seq were conducted using an MGI DNBSEQ-T7 (MGI Tech Co., Ltd. Shenzhen, China) sequencer with a 150-bp paired-end sequencing strategy (insert size: 300 bp). Given the relatively high error rate of ONT reads, the error profile of the ONT data was estimated using SeqFaiLR (Tools To Analyse Long Reads Sequencing Error Profile) [22].

For population genetic studies on *O. purpureiflora*, leaf samples were collected from 153 individuals representing six (sub)populations in LFS and NKS (Table 1, Fig. 2A). These individuals were randomly selected to ensure that their distribution covered the entire range of the two sites based on a thorough field investigation [11]. The geographical positions of the sampled individuals were recorded using a handheld GPS. Leaves from each individual were immediately placed into sealed plastic bags containing silica gel for preservation. Whole-genome resequencing was conducted using an MGI DNBSEQ-T7 sequencer with a PE-150bp model, generating approximately 30 Gb of data per sample.

Leaf and flower RNA-seq libraries were constructed and sequenced by Annoroad Gene Technology (AGT, Beijing, China), and the remaining libraries were constructed and sequenced by GrandOmics Biosciences (GB, Wuhan, China).

For *O. emarginata* and *O. semicastrata*, Hi-C libraries were constructed using leaf samples from the same individuals used in their primary genome assembies [15], and sequencing was performed by GrandOmics Biosciences.

Detailed sequencing information, including DNA/RNA preparation and library construction (except for ONT ultra-long WGS sequencing), has been reported in our previous studies [23, 24]. Specifically, ONT long-read WGS sequencing was performed using the protocol described by Wang et al. [23], whereas short-read WGS, Hi-C, and RNA-seq sequencing were conducted using protocols described by Wang et al. [24]. For *O. purpureiflora* ONT ultra-long WGS sequencing, genomic DNA was extracted, and approximately 8–10 μg of DNA fragments longer than 50 kb were selected using the SageHLS HMW library system (Sage Science, Inc., MA, USA). The size-selected DNA was repaired using the NEBNext FFPE DNA Repair Mix (Cat #M6630, New England Biolabs, MA, USA) in accordance with the manufacturer's instructions. End-repair and dA-tailing were then performed using the NEBNext Ultra II End-Repair/dA-tailing Module (Cat #E7546, New England Biolabs). Ligation of adaptors was performed by adding Adaptor Mix (SQK-LSK114, Oxford Nanopore Technologies, OX, Uk). The adaptor-ligated DNA was cleaned and quantified using fluorometry (Qubit 3.0, Thermo Fisher Scientific Inc., MA, USA) before library construction. The final library was sequenced on the Nanopore PromethION platform by using the R10.4.1 flowcell (Oxford Nanopore Technologies). Basecalling was performed using Dorado (RRID) v. 0.3.4 [25].

For genome assembly, annotation, and comparative genome analysis, default parameters were used in all programs unless otherwise specified.

Date preprocessing

Short WGS reads of *O. purpureiflora* and Hi-C reads from all three *Ormosia* species were quality-trimmed using Sickle v1.33 (RRID:SCR_006800) [26]. Reads had base quality values below 30 or lengths shorter than 80 bp were removed. The WGS reads of *O. purpureiflora* were further error corrected using RECKONER v1.1 [27]. Based on the error-corrected reads, 21-mer frequencies were generated using Jellyfish 2.3.0 (RRID:SCR_005491) [28], and the results were analyzed using GenomeScope 2.0 (RRID:SCR_017014) [29] to estimate the genome size, heterozygosity, and repetitiveness of *O. purpureiflora*. The ploidy level of *O. purpureiflora* was determined using nQuire with the "lrdmodel" function [30]. For ONT (ultra-)long WGS reads of *O. purpureiflora*, adapters were removed using Porchop 0.2.4 [31]. ONT reads larger than 20 kb were then extracted from the full dataset and defined as the 20-kb ONT read set, which was subsequently used for *O. purpureiflora* genome assembly.

Genome assembly

Using the 20-kb ONT read set, the *O. purpureiflora* genome was assembled using NextDenovo 2.3.1 [32]. After assembly, Pseudohaploid [33] and Purge_Dups v1.2.6 (RRID:SCR_021173) [34] were used to identify and remove duplications resulting from heterozygosity. The assembly was then polished sequentially by Racon v1.5.0 (RRID:SCR_017642) [35] (run twice), Hapo-G v1.3.2 [36] (run twice) and Polypolish v0.5.0 [37]. Depthcharge v0.2.0 [38] was applied to correct potential misassemblies, and contigs shorter than 1000 bp were removed. The corrected assembly was scaffolded using Hi-C reads with Scaffhic v1.1 [39], the Juicer pipeline 1.6 (RRID:SCR_017226) [40] and 3d-dna 201008 (RRID:SCR_017227) [41]. Gaps in the scaffolded assembly were closed with TGS-GapCloser v1.2.1 (RRID:SCR_017633) [42]. The gap-closed assembly was polished again using Racon, Hapo-G and Polypolish. Redundans 0.14a [43] was employed to remove redundant sequences unanchored to chromosomes. The assembly was then uploaded to GenBank to check for possible contamination. Sequences identified as bacterial and fungal contaminants were

removed. Subsequently, telomeric repeats at each chromosome ends were identified (with the parameter of "--motifs TTTAGGG –matchAny") and recovered using Teloclip v0.0.3 [44]. The assembly was then polished by Racon, Hapo-G and Polypolish to produce a complete genome assembly. To evaluate the assembly completeness, Benchmarking Universal Single-Copy Orthologs (BUSCO) v5.5.0 (RRID:SCR_015008)) [45] was applied using the eudicots_odb10.2020-09-10 database, which contains 2326 conserved eudicot core genes. Assembly quality was further assessed using AssemblyQC v. 2.1.1 [46] and GAEP v. 1.2.3 [47]. AssemblyQC provided metrics such as the Long Terminal Repeat (LTR) Assembly Index (LAI) [48], which evaluates contiguity based on repetitive sequences, as well as k-mer-based assembly completeness [49]. GAEP offered mapping-based evaluations, reporting read mapping ratios for various read types (long WGS, short WGS, and RNA-seq reads) and a consensus quality value (QV) for overall mapping accuracy.

For *O. emarginata* and *O. semicastrata*, the primary assemblies [15] were upgraded to chromosome-scale assemblies following the *O. purpureiflora* procedures, starting from Hi-C scaffolding. The final assemblies were evaluated for quality by using the same methods applied to *O. purpureiflora*.

Repeat sequence and gene prediction

The repeat sequences in three *Ormosia* chromosome-scale assemblies were identified using both EDTA v2.1.0 (RRID:SCR_022063) [50] and RED v2.0 [51]. The results from both programs for each assembly were combined and used to soft-mask the corresponding assembly with Bedtools v2.29.2 (RRID:SCR_006646) [52] using the commands "merge" and "maskfasta." During the merging process, the overlapping repetitive regions identified using the two programs, including fully overlapping regions and adjacent but nonoverlapping connected regions (if present), were merged into a single entry. Nonoverlapping repetitive regions identified uniquely using EDTA or RED, with no overlap in any part, were treated as distinct entries and included in the overall results.

The soft-masked *Ormosia* assemblies were annotated using BRAKER2 v.2.0 [53] and the Funannotate pipeline v1.8.16 [54]. BRAKER2 utilized RNA-seq reads and reference proteins from eight species (Table S1) for transcriptome- and homology-based annotation, except for *ab initio*-based gene prediction. The results from BRAKER2 were integrated using Funannotate to generate

consensus gene sets. Gene prediction in Funannotate followed three steps: “train”, “predict” and “update”. For the “predict” and “update” steps, the parameters “-max_intronlen 100,000 -busco_db embryophyta -organism other” were applied. Function annotation of predicted genes in *Ormosia* species was performed using Funannotate with the “annotate” command. The annotation databases included dbCAN v10.0 (RRID:SCR_013208) [55], EggNOG v5.0.2 (RRID:SCR_002456) [56], Gene Ontology (GO, RRID:SCR_002811) [57, 58], Kyoto Encyclopedia of Genes and Genomes (KEGG, RRID:SCR_012773) [59], InterPro v5.62-94.0 (RRID:SCR_006695) [60], MEROPS v12.0 [61] (RRID:SCR_007777), Pfam v35.0 [62] (RRID:SCR_004726), SignalP 5.0b (RRID:SCR_015644) [63] and UniProt v2023_02 (RRID:SCR_002380) [64].

The completeness of the predicted genes was initially evaluated using BUSCO with the eudicots_odb10.2020-09-10 database, analyzing the longest transcripts from each *Ormosia* assembly. In addition, prediction quality was assessed using the online tool OMArk v. 0.3.0 [65]. Unlike BUSCO, which focuses solely on conserved single-copy genes, OMArk evaluates completeness based on conserved genes in both single and multiple copies. It also examines the consistency of the predicted genes relative to closely related species (e.g., the proportion of genes in the same lineage) and identifies potential contamination events. Finally, the completeness of the predicted genes was examined against 15,345 representative gene models from 12 Fabaceae species [66]. For this analysis, the gene models of each comparative species (Table S2) were matched to the representative genes by using blastp (RRID: SCR_004870) v. 2.13.0 [67] with the parameters of “-evalue 1e-2 -outfmt 6 -num_threads 96 -max_hsps 5 -max_target_seqs 5”.

For the comparative genomic analyses, only the longest transcript for each gene across all species was used, unless stated otherwise. Additionally, for genome comparisons, the protein-coding genes of all other species used in our phylogenetic analysis (see below) were functionally annotated following the same procedures applied to the *Ormosia* species.

Gene family and comparative genomics

Orthologous groups (gene families) in *Ormosia* were identified using OrthoFinder 3.0.0 (RRID:SCR_017118) [68, 69], with protein-coding gene sequences from 17 other species (Table S2) as inputs. Phylogenetic analysis was subsequently performed using 1131 single-copy orthologs

inferred using OrthoFinder, employing with STAG [70] and STRIDE [71], which are integrated within OrthoFinder. The gene family file generated using OrthoFinder was further analyzed to assess gene family expansion or contraction using CAFE v5 (RRID:SCR_018924) [72]. The species tree, along with divergence time required for CAFE analysis, was constructed using MCMCTree [73], with 12 calibration points from the TimeTree database (http://timetree.org/, Table S3) for calibration. Following the CAFE analysis, GO and KEGG enrichment analyses were performed on the significantly expanded and contracted gene families in *O. purpureiflora* using TBtools v2.030 [74].

Gene duplications, synteny and structural variation analysis

Ancient whole genome duplication (WGD) events in *Ormosia* and their sister species *Lupinus albus* (see results) were identified using wgd v1.1.2 [75]. Gene duplications in *Ormosia* were analyzed using Doubletrouble v0.99.1 [76], which classified the duplication origin into categories including WGD, tandem duplications, proximal duplications, transposed duplications and dispersed duplications [77]. In this analysis *L. albus* was used as an outgroup species. For genes resulting from WGD, tandem, and proximal duplications in *O. purpureiflora*, GO and KEGG enrichment analyses were performed using TBtools.

Syntenic regions within and between *Ormosia* and *L. albus* genome assemblies were identified using MCScanX [78] and visualized using Shinycircos [79] or SynVisio [80]. The parameter of "-s 30" (MATCH_SIZE) was used for synteny analysis in MCScanX. Structural variations were identified using chromeister v1.5.a [81] and plotsr v1.1.0 [82].

Identification of nucleotide binding leucine-rich repeats and other resistance genes

Nucleotide binding leucine-rich repeats (NLR) genes are the primary plant resistance (*R*) genes that protect against viruses, bacteria, nematodes, fungi, oomycetes and insects [83, 84]. These genes typically consist of three canonical domains: a variable N-terminal domain, a central nucleotide-binding domain (NB-ARC) and a C-terminal domain composed of leucine-rich repeats (LRRs) [85]. At the N terminus, three types have been identified: Toll/interleukin-1 receptor (TIR), coiled-coil (CC) and resistance to powdery mildew8 (RPW8) [86]. The InterPro/Pfam entries associated with these domains include NB-ARC (IPR002182/PF00931), TIR (IPR000157/PF01582/PF13676), CC

(IPR038005), RPW8 (IPR008808/PF05659) and LRR (IPR001611/PF00560/, IPR013101/PF07723, IPR011713/PF07725, IPR025875/PF12799, IPR026906/PF13306, IPR001611/PF13516/PF13855, PF14580 and IPR032675). In addition to NLR genes, other *R* genes were identified based on their InterPro entries, as described by De-la-Cruz et al. [87]. Using gene annotation results from all species (three *Ormosia* species and the 17 comparative species listed in Table S2), obtained using the "annotate" command from the Funannotate pipeline, the InterPro/Pfam entries of their genes were matched to the corresponding *R* gene entries. The types and statistics of *R* genes were subsequently categorized for each species.

Transcription factor

Transcription factor (TF) genes in the genomes of *Ormoisa* species and the other species were identified by TF prediction online tools (https://planttfdb.gao-lab.org/prediction.php) [88].

Single-nucleotide polymorphism calling

Single-nucleotide polymorphisms (SNPs) in 153 *O. purpureiflora* individuals were identified using a modified dDocent v2.7.6 [89–91], NGSEP (RRID:SCR_012827) v. 5.0.0 [92], and GATK (RRID:SCR_001876) v. 4.6.0.0 [93], with the *O. purpureiflora* genome assembled in this study serving as the reference. A schematic of the SNP-calling workflow is shown in Fig. S1.

The dDocent pipeline, originally designed for SNP calling in RAD-seq (restriction-site associated DNA sequencing) data, is also suitable for other types of NGS data. For RAD-seq data, dDocent includes a specific RAD assembly step for accurate SNP calling in the absence of a reference genome. When a reference genome is available, the assembly step is omitted, and the provided reference genome is used to map resequencing data with BWA v. 0.7.17-r1188 [94]. SNP calling is then performed using Freebayes (RRID:SCR_010761) v. 1.3.6 [95]. Compared with other SNP-calling software, Freebayes has demonstrated strong performance [96–103], with its accuracy further enhanced by filtering to eliminate possible false positives [96, 97, 99, 102, 104]. After SNP calling, InDels, low-quality SNPs and SNPs deviating from Hardy–Weinberg equilibrium (HWE) were filtered out according to the "SNP Filtering Tutorial" from dDocent [105] and the code provided by Wang et al. [91]. The code is available as listed in the "Data Availability" section.

SNP calling in NGSEP used the mapping results from BWA after the dDocent pipeline was executed. For NGSEP, the parameters -h 0.00952 –maxAlnsPerStartPos 2 were used, with all other settings remaining at their defaults. The -h parameter specifies the heterozygosity rate, which was derived from the GenomeScope results (see Results). The raw SNPs called by NGSEP were quality-filtered using VCFtools (RRID) v. 0.1.17 [106], with the parameters of "--max-missing 0.95 --maf 0.05 --recode --recode-INFO-all --out try --min-meanDP 20 --mac 3 --minQ 30 --non-ref-af 0.001 --max-non-ref-af 0.9999". Filtered SNPs were further processed to remove the SNPs deviating from HWE and the InDels.

Due to the extremely slow runtime of GATK, it was not used for full SNP calling across the genome. Instead, GATK was used to recall SNPs detected using dDocent and NGSEP. For the recall process, the parameters of "--native-pair-hmm-threads 24 --base-quality-score-threshold 20 --minimum-mapping-quality 20" were used, with other settings left as defaults. After recalling, the two datasets were combined, and the successfully recalled SNPs (including SNPs co-identified by dDocent and NGSEP) were retained. The retained SNPs were filtered using the VariantFiltration command in GATK, with the following criteria: QD < 2.0 QUAL < 30.0 SOR > 3.0 FS > 60.0 MQ < 40.0 MQRankSum < −12.5 ReadPosRankSum < −8.0. This filtering process generated the "SNP Data Set-1," which was used to characterize the general SNP distribution, including SNP densities along chromosomes in the *O. purpureiflora* genome assembly.

To accurately infer population genetic diversity and structure, SNPs common to the dDocent- and NGSEP-recalled results were selected. These SNPs were further filtered to remove those in linkage disequilibrium (LD) by using Plink (RRID) v. 1.90p [107–109]. Specifically, SNP loci with an LD association coefficient (r^2) greater than 0.2 were excluded. Subsequently, only biallelic SNPs that were not missing across all genotyped individuals were retained using vcftools. Finally, SNPs under selection (i.e., adaptive SNPs) were identified and removed from the dataset used for genetic diversity and structure inferences. These SNPs were detected using PCAdapt v4.3.5 [110, 111] and BayPass v2.4 [112]. The resulting filtered SNPs comprised "SNP Data Set-2."

For PCAdapt, a principal component analysis (PCA) was first performed, and a Scree plot was used to determine the optimal number of PCs for regression with each SNP. Following regression analysis, SNPs with a *q* value (adjusted *P* value) of <0.01 were considered outliers, potentially under

selection. For BayPass, the core model with default parameters was applied. This model estimated an F_{ST}-like XtX statistic while accounting for the variance–covariance structure. To determine significance, a calibrated threshold (99%) was established by simulating pseudo-observed datasets (100,000 SNPs). SNPs falling within the 99.9% quantile of the pseudo-observed XTX distribution were considered candidates potentially under selection. Adaptive SNPs were identified as those occurring in both PCAdapt and BayPass results.

Genetic diversity and genetic structure

Genetic diversity parameters, including observed heterozygosity (*Ho*), expected heterozygosity (*He*) and inbreeding coefficient (*Fis*), were estimated by VCFtools. Nucleotide diversity within populations (π), nucleotide divergence between populations (*dxy*), and pairwise genetic differentiation (*Fst*) were calculated using pixy v1.2.7.beta1 [113].

The genetic structure of *O. purpureiflora* was inferred through PCA and ADMIXTURE (RRID:SCR_001263) [114]. PCA was performed using SNPRelate v1.36.0 [115], and ADMIXTURE was conducted using the AdmixPipe v3.2 pipeline [116]. In AdmixPipe, the number of potential genetic groups (*K*) was tested from 1 to 6, with 20 replicates for each *K* value. The best *K* value was determined based on cross-validation (CV) errors. For the inferred *K*, CLUMPAK v1.1 [117] was used to estimate the mean membership coefficients for individuals across the 20 replicates

Results

Chromosome number

The ploidy level estimated using nQuire indicated that the *O. purpureiflora* genome is diploid because the diploid model showed a lower delta likelihood than the free model (diploid delta likelihood: 1,609,982.99; triploid delta likelihood: 2,029,931.52; tetraploid delta likelihood: 2,270,455.24) (Table S4). Similar estimations for *O. emarginata* and *O. semicastrata* confirmed that these species also have diploid genomes.

The chromosome number of *O. purpureiflora* was determined to be 2n = 16 (Fig. S2), consistent with the number reported previously in *O. macrocalyx* [118] and *O. arborea* [119].

Genome sequencing

For *O. purpureiflora*, the ONT sequencing platform generated approximately 181.6 Gb of WGS reads, including 51.3 Gb ultra-long reads. The short sequencing platform produced approximately 139.3 Gb WGS reads and 146.8 Gb Hi-C reads. RNA-seq data amounted to approximately 20.4 Gb, 21.9 Gb, 23.3 Gb and 25.3 Gb for leaf, flower, fruit and seed samples, respectively. For *O. emarginata* and *O. semicastrata*, 148.7 Gb and 123.6 Gb Hi-C reads were generated, respectively,

Genome assembly

For *O. purpureiflora*, the genome size estimated using GenomeScope was 1,503,292,231 bp, with repetitive sequences accounting for 66.6% of the genome and a heterozygosity rate of 0.952% (Fig. S3). The initial genome assembly size was 1,811,176,403 bp, comprising 313 contigs with an N50 of 50,908,349 bp. After redundancy removal, Hi-C scaffolding and gap closing, the final assembly measured 1,584,128,722, with 1,583,483,254 bp (99.96%) anchored to 8 chromosomes (Table 2, Fig. 3A), consistent with chromosome number observation (Fig. S2). The longest chromosome was 259,935,025 bp long, and the shortest was 121,398,155 bp.

The initial assemblies for *O. emarginata* and *O. semicastrata* were 1,420,917,605 bp and 1,511,766,959 bp, respectively [15]. GenomeScope estimations using a k-mer size of 21 revealed repeat contents of 65.5% and 63.4%, and heterozygosity rates of 2.29% and 2.05% for *O. emarginata* and *O. semicastrata*, respectively. Both species displayed higher heterozygosity than *O. purpureiflora*, although the repeat content was similar across the three species. After incorporating Hi-C data, the assembly sizes of *O. emarginata* and *O. semicastrata* were refined to 1,420,253,666 and 1,510,687,319 bp, respectively (Table 2). Each assembly achieved 8 chromosome-level scaffolds, which accounting for 99.99% and 99.97% of the total in *O. emarginata* and *O. semicastrata*, respectively.

For *O. purpureiflora*, BUSCO evaluation revealed 98.3% complete BUSCOs, with 89.4% of them being single-copy BUSCOs and 8.9% being duplicated BUSCOs. In addition, 0.3% BUSCOs were fragmented, and 1.4% were missing. For *O. emarginata*, the complete BUSCO score was 97.0%, including 89.4% complete and single-copy BUSCOs and 7.6% complete but duplicated

BUSCOs, with 0.5% fragmented and 2.5% missing. Similarly, *O. semicastrata* achieved a complete BUSCO score of 98.4%, consisting of 90.4% complete and single-copy BUSCOs and 8.0% complete but duplicated BUSCOs, with fragmented and missing BUSCOs constituting 0.1% and 1.5%, respectively.

The LAI values for the three *Ormosia* assemblies were all above 10, ranging from 13.66 to 17.56 (Table 2), meeting the quality standard for a "Reference genome." Mapping-based evaluations indicated that all types of reads achieved high mapping ratios, exceeding 91%. However, the k-mer and mapping-based quality value (QV) scores in all three *Ormosia* assemblies were below 40, a threshold that corresponds to 99.99% base accuracy and is considered high quality for genome assemblies [47, 49]. In addition, k-mer-based completeness was below 90% across all *Ormosia* assemblies, with *O. purpureiflora* having the highest completeness at 88.36% and *O. emarginata* having the lowest at 78.04%. These assessments suggest that further improvements in the assemblies are warranted.

Assembling genomes with a large size (>1 Gb), high repeat content (>50%), and elevated heterozygosity (>0.5%) presents significant challenges [120], and the species examined in this study exhibited all these features. To address these issues, we employed the Nanopore sequencing platform, which generates reads that are longer in length than those produced by the PacBio sequencing platform, particularly in Hi-Fi sequencing mode [121]. For *O. purpureiflora*, we included ultra-long reads (>50 kb) to enhance assembly continuity. In the assembly process, reads longer than 20 kb were used for assembling the *O. purpureiflora* genome, whereas reads longer than 10 kb were used for assembling the genomes of *O. emarginata* and *O. semicastrata*. Programs such as Pseudohaploid and Purge_Dups were used to remove heterozygous contigs and regions, effectively mitigating challenges associated with high repeat content and heterozygosity in these genomes. However, ONT reads generally have high sequencing error rates, ranging from 5% to 20% [121, 123]. For *O. purpureiflora*, the error rate of ONT reads was 15.18%, as assessed using the 20 kb ONT read set employed for genome assembly (see the "Methods" section). Similarly, error rates of 17.75% and 16.82% were observed in the 10-kb ONT read sets of *O. emarginata* and *O. semicastrata*, respectively. Given these limitations, haplotype-resolved de novo genome assembly was not performed for the three *Ormosia* species. Future studies should incorporate highly accurate Hi-Fi

long-read sequencing technology and phasing steps to optimize the current assemblies and improve their overall quality.

Repeat and gene annotation

RED analyses identified 1,037,006,095 bp (65.5%), 885,912,252 bp (62.4%) and 968,176,023 bp (64.1%) of repetitive sequences in *O. purpureiflora*, *O. emarginata* and *O. semicastrata*, respectively. EDTA analyses revealed higher percentages, that is, 1,139,417,595 bp (71.9%), 989,514,254 bp (69.6%), and 1,074,353,470 bp (71.1%) of repetitive sequences in *O. purpureiflora*, *O. emarginata* and *O. semicastrata*, respectively (Table S5). After combining the results from RED and EDTA, the total repetitive components were found to be 1,209,324,791 bp (76.3%) in *O. purpureiflora*, 1,051,218,280 bp (74.0%) in *O. emarginata* and 1,135,447,010 bp (75.2%) in *O. semicastrata*. According to EDTA analyses, the *Gypsy*-like long terminal repeat retrotransposon (LTR-RT) family represents the most abundant repetitive sequence, comprising 33.51%, 35.45%, and 27.73% of the genome assemblies for *O. purpureiflora*, *O. emarginata* and *O. semicastrata*, respectively.

Gene prediction identified 55,061 genes encoding 59,809 proteins in *O. purpureiflora*. For *O. emarginata* and *O. semicastrata*, the predictions revealed 50,517 and 51,220 genes encoding 54,456 and 55,363 proteins, respectively (Table 3). Table 3 also provides statistics on various gene features in the three species' assemblies. Overall, *O. purpureiflora* exhibited the lowest average number of exons and introns per gene as well as the shortest average gene and CDS lengths. Approximately 70.81%, 76.43% and 72.43% of protein-coding genes in *O. purpureiflora*, *O. emarginata* and *O. semicastrata*, respectively, were functionally annotated in at least one database (Table 3). Comparatively low annotation rates were also observed in other genomes, such as *Senna tora* (67.16%), *Pisum sativum* (72.70%), and *Sesbania bispinosa* (78.15%; Table S6). By contrast, higher annotation rates were reported in agriculturally important species such as *Glycine max* (99.03%), *Cajanus cajan* (98.97%), *Cicer arietinum* (98.84%), and *Vigna unguiculata* (98.55%), whose genomes have received greater research attention, contributing to more functional information in annotation databases. The low annotation rates in *Ormosia* species may be attributed to the presence of novel genes with unknown functions, which are less represented in the current annotation

databases.

Gene prediction completeness, as assessed using BUSCO, indicated a completeness score of 96.1% in *O. purpureiflora* (88.8% complete and single-copy, 7.3% complete but duplicated), with 1.5% fragmented and 2.4% missing genes. For *O. emarginata*, the BUSCO analysis revealed 95.1% completeness (88.6% complete and single-copy, 6.5% complete but duplicated), with 1.5% fragmented and 3.4% missing genes. In *O. semicastrata*, the completeness score scores was 96.3% (89.6% complete and single-copy, 6.7% complete but duplicated), with 1.4% fragmented and 2.3% missing genes.

OMArk evaluations reported a completeness score of 97.9% for *O. purpureiflora* (67.0% single-copy, 30.0% duplicated), with 2.1% missing genes. Of the predicted genes, 60.4% were consistent, 3.2% were inconsistent, and 36.39% are unknown (Table S7). For *O. emarginata*, OMArk indicated 97.1% completeness (67.0% single-copy, 30.0% duplicated), with 2.9% missing; 62.8% consistent, 2.5% inconsistent, and 34.66% unknown genes. For *O. semicastrata*, OMArk reported 98.1% completeness (67.8% single-copy, 30.3% duplicated), with 1.9% missing, 62.8% consistent, 2.8% inconsistent, and 34.37% unknown genes. No contamination was detected in the gene sets of any of the three *Ormosia* species. Compared with other species, *Ormosia* exhibited a higher proportion of duplicated and unknown genes and lower consistency. Similarly high levels of duplicated genes were observed in *Ormosia*' s sister species, *L. albus* (37.77%), which may be attributed to lineage-specific WGD events (see "Gene duplications, synteny, and structural variation analysis" section). The low consistency scores are likely linked to the high proportion of unknown genes. The high proportion of unknown genes in *Ormosia* may result from newly identified genes that lack homologs in OMArk's reference databases, reflecting the limited genomic information available for this lineage. A similar trend of high unknown gene rates (39.07%) and low consistency (56.99%) in *Senna tora* may also be explained by the same factor.

The *Ormosia* genes showed high matching rates with Fabaceae representative genes (Table S8), ranging from 73.41% to 73.91%. These rates were only slightly lower than those observed for *Medicago truncatula* (75.68%) and *Pisum sativum* (75.36%), supporting the completeness of the predicted *Ormosia* genes.

Gene density distribution in *Ormosia* was found to correlate with repeat density distribution

along each chromosome (Fig. 3B), suggesting potential repeat-mediated gene formation. On chromosomes 3, 4, 6, 7 and 8, regions with low repeat and gene density were generally located in the middle of the chromosome. By contrast, for chromosomes 1, 2 and 5, high densities were observed on one arm of the chromosome, while low densities were noted on the other arm. In contrast to *Ormosia*, no such gene-repeat density correlation was observed in its sister species, *L. albus* [17], indicating that this pattern may be specific to *Ormosia*. A closer examination of the repeat types revealed that Helitrons and Terminal Inverted Repeats (TIRs) were specifically associated with gene distribution in the *Ormosia* assemblies (Fig. 4 and Fig. S4).

According to InterPro functional annotation, we found that some photosynthesis-related genes were not annotated in *O. purpureiflora* when compared with *O. emarginata* and *O. semicastrata* (Table S9). Specifically, the number of genes associated with Photosystem I PsaA/PsaB (IPR001280) in *O. purpureiflora* was 3, which was lower than the number of genes in *O. emarginata* (8) and *O. semicastrata* (9). In addition, the InterPro database showed the absence of annotation in several genes related to plant–pathogen interaction (EDS1-like, IPR044214), plant reproduction (DBP10, C-terminal, IPR012541), pyrimidine/nucleotide metabolism (deoxyuridine triphosphate nucleotidohydrolase, IPR008181; dUTPase-like, IPR029054/IPR036157), regeneration (Thioredoxin DCC1, IPR044691), seed maturation protein 1 (SMP1, IPR044984) and nodulin (IPR003387) in *O. purpureiflora*.

Gene family

A total of 47,608 gene families were identified using OrthoFinder. In *O. purpureiflora*, 50,275 genes (91.3%) were assigned to 27,347 gene families. Among these, 454 families were specific to *O. purpureiflora* (Table S10). The genes in these families were mainly enriched in processes such as endoplasmic reticulum to Golgi vesicle-mediated transport and non-membrane-bounded organelle assembly in GO's BP category (Table S11) and ribosome biogenesis in eukaryotes in the KEGG analysis (Table S12).

The phylogenetic tree (Fig. 3C) indicated that *O. purpureiflora* was sister to *O. emarginata* and that *Ormosia* was sister to *L. albus*. The estimated divergence time between *O. purpureiflora* and *O. emarginata* was approximately 2.94 million years ago (95% CI: 1.19–5.00), whereas the divergence

time between *Ormosia* and *Lupinus* was 45.90 million years ago (95% CI: 32.66, 56.44). In *O. purpureiflora*, 1020 gene families were expanded and 623 were contracted. Among these, the expansion and contraction were significant in 205 and 84 gene families ($P < 0.05$). Significantly expanded gene families were mainly enriched in DNA integration and regulation of amino acid transmembrane transport in GO's biological process (BP) category (Table S13) and alkaloid, polyketide and zeatin biosynthesis in the KEGG analysis (Table S14). The significantly contracted gene families were mainly associated with transcription by lipid transport and lipid localization in the GO's BP category (Table S15) and with terpenoid biosynthesis in the KEGG analysis (Table S16).

The genes in the contracted gene families related to terpenoid biosynthesis were primarily Cytochrome P450 (CYP450) genes, which are responsible for downstream activities in the final terpenoid products [124, 125]. However, terpenoids were mostly represented by two conserved domains with Pfam IDs of PF01397 and PF03936 [90]. A comparison showed that the *O. purpureiflora* assembly annotated 23 and 25 of these genes, slightly fewer than those in *O. emarginata* (31 and 26) and *O. semicastrata* (26 and 28). Nevertheless, the number of genes in *Ormosia* species was much higher than that in their sister species, *L. albus* (8 and 10).

Gene duplications, synteny, and structural variation analysis

WGD analysis indicated that *O. purpureiflora* has undergone a recent WGD event (Fig. 3D), which was shared with the other two *Ormosia* species and *L. albus*. Therefore, this WGD event is not specific to *Ormosia* but instead may be specific to the Genistoid lineage in Fabales [16, 17]. Future studies, including newly published Fabaceae genomes, will help confirm this hypothesis.

Gene duplication analysis revealed that the three *Ormosia* species exhibited similar numbers of genes across different duplication types (Table S17). In *O. purpureiflora*, enrichment analysis showed that WGD-duplicated genes were primarily associated with the processes related to calcium ion, blue light, flower and development, and cytokinin biosynthetic process in the GO's BP category (Table S18). In KEGG analysis, these genes were linked to signaling proteins, glycosylphosphatidylinositol (GPI)-anchored proteins, GTP-binding proteins and SNARE interactions in vesicular transport (Table S19). Tandem-duplicated genes were mainly associated

with phloem development, glutathione metabolic process and the biosynthesis of monoterpenoid, anthocyanin, zeatin, and flavonoid (Table S20 and S21). Proximal-duplicated genes were predominantly involved in diterpenoid and triterpenoid biosynthetic process, arginine biosynthetic process, phloem development, and flavone and flavone biosynthesis (Table S22 and S23). These results were consistent with those of the previous study on *O. emarginata* and *O. semicastrata* by Liu et al. [15], which showed that tandem and proximal duplicated genes were relevant to various (secondary) biosynthetic and metabolic processes, including the biosynthesis of alkaloid, flavonoid, and terpenoid.

Synteny analysis within *Ormosia* revealed 48, 42 and 45 syntenic blocks in *O. purpureiflora*, *O. emarginata* and *O. semicastrata*, respectively (Table S24). The longest syntenic blocks identified in these species were between chromosomes 2 and 3. These blocks measured 39,614,256 bp and contained 427 gene pairs in *O. purpureiflora*, 33,895,706 bp with 383 gene pairs in *O. emarginata*, and 36,266,649 bp with 424 gene pairs in *O. semicastrata*. The syntenic relationships were illustrated in the Circos plot (Fig. 3B).

Overall, *O. purpureiflora* genome exhibited highly syntenic relationships with the other two *Ormosia* genomes, as shown by both synteny analysis (Fig. 3E) and dot plots (Fig. S5). However, further genetic variation analysis revealed extensive intra-chromosomal rearrangements among the *Ormosia* species (Fig. 3F). These rearrangements were primarily concentrated in specific "hot" chromosomal regions, where the gene density was low, indicating unstable genome architecture in these regions, while gene-rich regions maintained a more conserved genome structure in *Ormosia*.

O. semicastrata exhibited greater divergence from *O. purpureiflora* and *O. emarginata*, as evidenced by the high unaligned proportions (52.36% unalignment with *O. purpureiflora* assembly and 50.54% unalignment with *O. emarginata* assembly) compared with lower unaligned proportions between *O. emarginata* and *O. purpureiflora* (24.88% and 28.51%, respectively, Table S25). Furthermore, *O. semicastrata* exhibited fewer translocations and duplications than *O. purpureiflora* and *O. emarginata*. These findings align with those of our phylogeny analysis (Fig. 3C) as well as previous results, which have reported that *O. emarginata* and *O. semicastrata* belong to different clades [1]. Although *O. purpureiflora* was sister to *O. emarginata* and thus in the same clade, the structural rearrangements observed between *O. emarginata* and *O. semicastrata* were not preserved

in *O. purpureiflora*.

Ormosia purpureiflora and *O. emarginata* exhibited the largest inversion on Chromosome 1, spanning from 166,804,741 to 222,962,103 bp in *O. purpureiflora* and from 127,118,909 to 186,491,244 bp in *O. emarginata*. Extensive duplications were also observed on the same chromosome. On Chromosome 2, significant translocations were detected. Notably, a *O. purpureiflora*-specific inverted region was identified on Chromosome 2, spanning from 52,506,652 to 61,757,520 bp (Fig. 3F and Fig. S5), which was located away from the rearrangement hot regions. This inverted region was 9,250,868 bp in length and contained 577 genes. The enrichment analysis of these genes revealed their involvement in osmotic stress and temperature regulation (Table S26), which may contribute to *O. purpureiflora*' s adaptation to rocky environments (Fig. 1F), thin soil layers (with low soil moisture content) and relatively high elevation (400–750 m in altitude) [11].

The hot structural rearrangement regions on the chromosomes of *Ormosia* were strongly correlated with the areas of highest repeat density, particularly those containing *Gypsy* and unknown LTR-RTs (Fig. 4). This suggests that repeat-mediated rearrangements play a crucial role in shaping the *Ormosia* genomes. Because all three *Ormosia* species displayed similar patterns of rearrangements on their chromosomes, these structural changes may have been inherited from their common ancestor. By contrast, the other LTR-RT type, *Copia* was generally evenly distributed along the chromosomes in *Ormosia*. The enrichment analysis revealed that *Copia*-mediated genes were primarily associated with mitosis, mismatch repair and secondary metabolite biosynthetic process in the GO's BP category (Table S27). In KEGG analysis, these genes were linked to processes such as glutathione metabolism, transcription machinery, replication and repair, and chromosome and associated proteins (Table S28). However, these results were not statistically significant after *p*-value correction procedures.

Genomic rearrangements are a significant driving force of evolution [126], although they have been less studied in the Fabaceae family [127]. In *Eucalyptus*, duplications and translocations have been shown to contribute most to genome divergence [128]. The mechanisms responsible for these rearrangements may include recombination, repair or replication, and they are closely linked to repetitive elements [129, 130]. The observed correlation between hot rearrangement regions and LTR-RTs in *Ormosia* suggests that this genus could serve as a valuable model system for studying

these mechanisms in the future.

Nucleotide binding leucine-rich repeats (NLR) and the other resistance (*R*) gene identification

Compared with other species, *Ormosia* species were found to have a higher number of *R* genes (Table S29). Specifically, the number and percentage of NLR genes in the *Ormosia* species were higher than those in the sister species *L. albus*. However, in terms of the other *R* genes, *L. albus* displayed a higher number and percentage than the *Ormosia* species. Among the *Ormosia* species, *O. purpureiflora* had a higher number and percentage of other *R* genes than *O. emarginata* and *O. semicastrata*. The distribution of *R* genes across the chromosomes of each *Ormosia* species is shown in Fig. S6. *R* genes were spread across all eight chromosomes, following a distribution pattern consistent with the overall gene distribution in *Ormosia* species.

Transcription factor

Ormosia species had a higher number of TF genes than all the other species, except for *Glycine max*, *Sesbania bispinosa, Acacia pycnantha* and their sister species *L. albus*. However, the percentage of TF genes in *Ormosia* species was relatively low, particularly in *O. purpureiflora* (3.96%, Table S30).

Single-nucleotide polymorphism calling

Initial SNP calling using the dDocent pipeline from 153 *O. purpureiflora* individuals yielded 313,309,883 loci, including 238,531,000 SNP loci and 74,778,883 InDel loci. After the filtering out of low-quality SNPs, SNPs deviating from HWE, and InDels, a total of 297,891 SNPs were retained. The raw SNPs called by NGSEP identified 37,875,127 loci, comprising 24,941,612 SNPs and 12,933,515 InDels. After quality filtering and HWE correction and InDel removal, 358,992 SNPs were retained.

GATK recalled 287,928 SNPs from the dDocent results (96.66% recovery rate) and 340,190 SNPs from the NGSEP results (94.76% recovery rate). After combining the two recalled datasets, a total of 561,922 SNPs (SNP Data Set-1) were identified, including 66,196 co-occurring SNPs. After LD filtering, 44,592 co-occurring SNPs remained. Further filtering retained 41,265 biallelic co-

occurred SNPs with no missing genotypes. PCAdapt analysis indicated that four main components were suitable to account for population structure, as shown in the Scree plot (Fig. S7). PCAdapt identified 5,006 candidate SNPs potentially under selection, whereas BayPass revealed 5,257 candidate SNPs. Across both analyses, 2,922 SNPs were identified as adaptive. Among these, 160 SNPs appeared in 80 genes; however, GO and KEGG analyses did not show significant enrichment. After the removal of adaptive SNPs, 38,343 SNPs remained in SNP Data Set-2, which was used for further genetic structure and diversity analyses.

Genetic diversity and genetic structure

Genetic diversity analyses in *O. purpureiflora* (sub)populations indicated that LFS1 and LFS3 exhibited the highest genetic diversity for two parameters (*He* and π), both with values of 0.460 and 0.468, respectively (Table 1). LFS4 displayed the highest diversity in observed heterozygosity (*Ho*), with a value of 0.882. In contrast, *Ho*, *He*, and π were found to be the lowest in LFS5, with values of 0.854, 0.449, and 0.456, respectively. All (sub)populations showed negative *Fis*, indicating an excess of heterozygosity. The overall *Fst* was 0.001, which was low, suggesting minimal population differentiation. Compared with *O. henryi*, a species more widely distributed in southern China, *O. purpureiflora* displayed higher genetic diversity but lower *Fis*. For *O. henryi*, the genetic diversity measures were *Ho*: 0.228–0.287, *He*: 0.237–0.290, π: 0.122–0.143, and *Fis*: −0.023–0.022 in) [131].

Heterozygosity excess in plants may be attributed to several factors, including polyploidy, reproduction mode (such as outcrossing, self-incompatibility systems, and clonal growth), demographic history (such as population bottlenecks), and natural selection (e.g., the overdominant phenomenon, where heterozygous individuals have high survival rates) [132–139]. *O. purpureiflora* is a diploid species, as mentioned earlier. Its flowers are insect-pollinated, exhibiting a typical outcrossing reproductive system. Given the small size of its populations, the limited reproduction among individuals reduces the likelihood of inbreeding and leads to a decrease in inbred offspring. Therefore, the reproductive system may favor heterozygous individuals in the population. *O. purpureiflora* also reproduces asexually through suckering [11], which contributes to the observed heterozygosity excess. However, the effects of other factors, such as a historical bottleneck, cannot be overlooked and warrant further investigation.

Genetic diversity parameters along the chromosomes revealed that the region from approximately 39–68 Mbp on Chromosome 5 had relatively high SNP density, π, dxy, *Ho* and *He* values but a lower *Fst* value (Fig. 2D). Balancing selection is a possible explanation for the observed patterns in this region, although further exploration is needed. The KEGG enrichment analysis functionally annotated the genes in this region to glycosyltransferases, translation and carbohydrate metabolism, however, the results were not statistically significant (Table S31). Glycosyltransferases drive glycosylation reactions, a key process in which monosaccharides are transferred from sugar donors to glycans, lipids, peptides, and small molecules [140–142]. These glycosylated biomolecules participate in various biological processes and play crucial roles in plant growth, development, defense and stress tolerance [142–144].

PCA revealed that the first PC separated LFS4 from the other (sub)populations (Fig. 2B). The second PC did not show a clear separation among the (sub)populations, whereas the third PC distinguished NKS from the others. In the ADMIXTURE analysis, the cross-validation (CV) error decreased consistently from K = 1 to K = 6 (Fig. S8), but from K = 4 onward, the decrease slowed down. Therefore, K = 4 was identified as the optimal number of genetic groups. Given the limited number of (sub)populations in *O. purpureiflora*, the present study reports the results for K = 2 to K = 6 (Fig. 2C). Overall, extensive admixture was observed in all subpopulations, especially in LFS1, LFS2, and LFS3, regardless of the optimal K value. At K = 2 and K = 3, LFS4 could be separated into one group, whereas LFS5 and NKS formed another group. At K = 4, LFS5 and NKS were further differentiated from the other subpopulations. However, when K was increased further, no additional clear groups were identified. Both PCA and ADMIXTURE analyses highlighted the distinctiveness of LFS4, although the reasons for this distinctiveness remain unclear.

Conclusion

Fabaceae play a crucial role in biological nitrogen fixation and serve as a source of nutrition for wild fauna, contributing to the health and balance of ecosystems. The same holds true for *Ormosia* species. Previous studies have shown that *Ormosia* species are rich in secondary metabolites, including alkaloids, terpenes and flavonoids, which warrant further exploration, particularly from a genomic perspective. The genomes of the two previous *Ormosia* species and the

current *O. purpureiflora* genome indicate that genes involved in the biosynthesis of these metabolites are often found in tandem duplications, proximal duplications, or are expanded. The association between gene distribution and repeats suggests that these repeats play a role in gene duplication, highlighting the need for future research on this topic. Thus, the high-quality *Ormosia* genomes serve as a valuable resource for understanding the efficiency of metabolite biosynthesis and identifying potentially useful chromosome regions (such as syntenic regions, structural rearrangements, and balancing selection) for future study.

Funding:

The study is supported by Guangdong Science and Technology Plan Project (2023A1111110001); Key-Area Research and Development Program of Guangdong Province (2022B1111230001) and its sub-project (2022B1111230001-2-5); Guangdong Provincial Forestry Bureau Project — Planning of the Provincial Plant Ex Situ Protection System and National Key Protected Plant Ex Situ Protection and Propagation; The National Natural Science Foundation of China (No. 32370406, 31970188); Guangdong Science and Technology Plan Project (grant No.: 2023B1212060046).

Data Availability

Raw sequenced reads have been uploaded to the NCBI Sequence Read Archive under accession number of SRR24060960 for short WGS reads, SRR24061088 and SRR24061087 for long WGS reads, SRR24085385 for ultralong WGS reads, SRR24112497 for Hi-C reads, SRR24044811 for fruit RNA-seq reads, SRR24044812 for seed RNA-seq reads, SRR24085891 for leaf RNA-seq reads, SRR24085890 for flower RNA-seq reads in *O. purpureiflora*; SRR25460826 for Hi-C reads of *O. emarginata*; SRR25460825 for Hi-C reads for *O. semicastrata*; SRR29820911-SRR29820936 for resequencing reads of LFS1, SRR29824870-SRR29824895 for resequencing reads of LFS2, SRR29837260-SRR29837285 for resequencing reads of LFS3, SRR29856316-SRR29856341 for resequencing reads of LFS4, SRR29887191-SRR29887216 for resequencing reads of LFS5, SRR29761002-SRR29761004, SRR29761010-SRR29761017, SRR29761028-SRR29761030, SRR29761107, SRR29761108, SRR29761114, SRR29761115, SRR29761118, SRR29761123,

SRR29761124, SRR29761126, SRR29761139 for resequencing reads of NKS in *O. purpureiflora*. Assembled genomes are under accession number of GCA_040955955.1 for *O. purpureiflora*, GCA_029884595.2 for *O. semicastrata* and GCA_029884605.2 for *O. emarginata*. Annotations, SNPs and the other files are submitted to figshare (https://doi.org/10.6084/m9.figshare.26826466.v14). Codes are available from Wang (2022) [91] and https://molecular-ecologist.com/col.jsp?id=103. Specifically, the SNP filtering procedure is provided in Chapter 9.

Competing Interests

The authors declare that they have no competing interests.

References

1. Torke BM, Cardoso D, Chang H, et al. A dated molecular phylogeny and biogeographical analysis reveals the evolutionary history of the trans-pacifically disjunct tropical tree genus *Ormosia* (Fabaceae). Mol Phylogenet Evol 2022;166:107329. https://doi.org/10.1016/j.ympev.2021.107329.
2. Niu M, Jiang K-W, Song Z-Q, et al. Two new synonyms of *Ormosia semicastrata* (Fabaceae, Papilionoideae, Ormosieae). Phytotaxa 2023;613(2):140-152. https://doi.org/10.11646/phytotaxa.613.2.3.
3. Wang Z, Shi G, Sun B, et al. A new species of *Ormosia* (Leguminosae) from the middle Miocene of Fujian, Southeast China and its biogeography. Rev Palaeobot Palyno 2019;270:40-47. https://doi.org/10.1016/j.revpalbo.2019.07.003.
4. Li L, Lei M, Wang H, et al. First report of dieback caused by *Lasiodiplodia pseudotheobromae* on *Ormosia pinnata* in China. Plant Dis 2020;104:2551-2555. https://doi.org/10.1094/PDIS-03-20-0647-RE.
5. Wei L, Wang G, Xie C. Predicting suitable habitat for the endangered tree *Ormosia microphylla* in China. Sci Rep 2024;14:10330. https://doi.org/10.1038/s41598-024-61200-5.

6. Zhang L-J, Zhou W-J, Ni L, et al. A review on chemical constituents and pharmacological activities of *Ormosia*. Chin Tradit Herbal Drugs, 2021;52(14):4433-4442. https://doi.org/10.7501/j.issn.0253-2670.2021.14.035.
7. Zhou Q-Q , Xie X-Y, Zhu J-W, et al. Hosimosines A-E, structurally diverse cytisine derivatives from the seeds of *Ormosia hosiei* Hemsl. et Wils. Fitoterapia, 2023;170:105661. https://doi.org/10.1016/j.fitote.2023.105661.
8. Zhou W, Quan Y, Chen Y, et al. A new lignan from leaves of *Ormosia xylocarpa*. Rec Nat Prod 2023;17(1):189-194. http://doi.org/10.25135/rnp.338.2203.2386.
9. Wang J, Li L, Wang Z et al. Integrative analysis of the metabolome and transcriptome reveals the molecular regulatory mechanism of isoflavonoid biosynthesis in *Ormosia henryi* Prain. Int J Biol Macromol 2023;246:125601. https://doi.org/10.1016/j.ijbiomac.2023.125601.
10. Wang J, Wang X, Deng X, et al. Analysis of candidate genes for terpene synthesis in *Ormosia henryi* based on metabolome and transcriptome. J Zhejiang A&F Univ 2023;40(5):970-981. https://doi.org/10.11833/j.issn.2095-0756.2022073.
11. Yu E-P. Preliminary study on conservation ecology of the rare and endemic plant *Ormosia purpureiflora* to Guangdong. Master's thesis. University of Chinese Academy of Sciences; 2024
12. Tang J, Zou R, Wei X, et al. Complete chloroplast genome sequences of five *Ormosia* species: Molecular structure, comparative analysis, and phylogenetic analysis. Horticulturae 2023;9(7):796. https://doi.org/10.3390/horticulturae9070796.
13. Wang Z-F, Yu E-P, Zeng QS, et al. The complete chloroplast genome of *Ormosia purpureiflora* (Fabaceae). Mitochondrial DNA B 2012;6(12):3327-3328. https://doi.org/10.1080/23802359.2021.1994901
14. Wang Z-F, Zhang Y., Zhong X-J, et al. The complete mitochondrial genome of *Ormosia boluoensis*. Mitochondrial DNA B 2021;6(8): 2109-2111. https://doi.org/10.1080/23802359.2021.1920503
15. Liu P-P, Yu E-P, Tan Z-J et al. Genome assemblies of two *Ormosia* species: Gene duplication related to their evolutionary adaptation. Agronomy 2023;13:1757. https://doi.org/10.3390/agronomy13071757.

16. Zhao Y, Zhang R, Jiang K-W, et al. Nuclear phylotranscriptomics and phylogenomics support numerous polyploidization events and hypotheses for the evolution of rhizobial nitrogen-fixing symbiosis in Fabaceae. Mol Plant 2021; 14(5):748-773. doi: 10.1016/j.molp.2021.02.006.
17. Xu W, Zhang Q, Yuan W, et al. The genome evolution and low-phosphorus adaptation in white lupin. Nat Commun 2020;11:1069. https://doi.org/10.1038/s41467-020-14891-z.
18. Hane JK, Ming Y, Kamphuis LG, et al. A comprehensive draft genome sequence for lupin (*Lupinus angustifolius*), an emerging health food: insights into plant–microbe interactions and legume evolution. Plant Biotechnol J 2017;15(3):318-330. https://doi.org/10.1111/pbi.12615.
19. Martinez-Hernandez JE, Salvo-Garrido H, Levicoy D, et al. Chromosome-level genome assembly of yellow lupin (*Lupinus luteus*) provides novel insights into genome evolution, crop adaptation and seed protein in the three most cultivated lupins. Res Square 2024. https://doi.org/10.21203/rs.3.rs-4171664/v1.
20. Feng L, Teng F, Li N, et al. A reference-grade genome of the xerophyte *Ammopiptanthus mongolicus* sheds light on its evolution history in legumes and drought-tolerance mechanisms. Plant Commun 2024; 5(7):100891. https://doi.org/10.1016/j.xplc.2024.100891.
21. Qu Z, Wang W, Adelson DL. Chromosomal level genome assembly of medicinal plant *Sophora flavescens*. Sci Data 2023;10:572. https://doi.org/10.1038/s41597-023-02490-8.
22. Delahaye C, Nicolas J (2021) Sequencing DNA with nanopores: Troubles and biases. PLoS ONE 2021;16(10):e0257521. https://doi.org/10.1371/journal.pone.0257521.
23. Wang Z-F, Rouard M, Droc G, et al. Genome assembly of *Musa beccarii* shows extensive chromosomal rearrangements and genome expansion during evolution of Musaceae genomes. GigaScience, 2023;12:giad005. https://doi.org/10.1093/gigascience/giad005.
24. Wang Z-F, Fu L, Yu E-P, et al. Chromosome-level genome assembly and demographic history of *Euryodendron excelsum* in monotypic genus endemic to China. DNA Res 2024; 31(1):dsad028. https://doi.org/10.1093/dnares/dsad028.
25. Oxford Nanopore's Basecaller. https://github.com/nanoporetech/dorado. Accessed 15 August 2023.
26. Joshi NA, Fass JN. Sickle: A sliding-window, adaptive, quality-based trimming tool for FastQ files (Version 1.33). 2011. https://github.com/najoshi/sickle. Accessed 3 September 2021.

27. Długosz M, Deorowicz S. RECKONER: read error corrector based on KMC. Bioinformatics 2017;33:1086-1089. https://doi.org/10.1093/bioinformatics/btw746.
28. Marçais G, Kingsford C. A fast, lock-free approach for efficient parallel counting of occurrences of k-mers. Bioinformatics 2011;27:764-770. https://doi.org/10.1093/bioinformatics/btr011.
29. Vurture GW, Sedlazeck FJ, Nattestad M et al. GenomeScope: fast reference-free genome profiling from short reads. Bioinformatics 2017;33:2202-2204. https://doi.org/10.1093/bioinformatics/btx153.
30. Weiß CL, Pais M, Cano LM, et al. nQuire: a statistical framework for ploidy estimation using next generation sequencing. BMC Bioinform 2018;19:122. https://doi.org/10.1186/s12859-018-2128-z.
31. Porechop (Version 0.2.4). https://github.com/rrwick/Porechop/releases/tag/v0.2.4. Accessed 8 January 2019.
32. Hu J, Wang Z, Sun Z, et al. NextDenovo: an efficient error correction and accurate assembly tool for noisy long reads. Genome Biol 2024;25:107 (2024). https://doi.org/10.1186/s13059-024-03252-4.
33. Pseudohaploid. https://github.com/schatzlab/pseudohaploid. Accessed 28 August 2020.
34. Guan DF, McCarthy SA, Wood J, et al. Identifying and removing haplotypic duplication in primary genome assemblies. Bioinformatics 2020;36:2896-2898. https://doi.org/10.1093/bioinformatics/btaa025.
35. Vaser R, Sović I, Nagarajan N, et al. Fast and accurate de novo genome assembly from long uncorrected reads. Genome Res 2017;27(5):737-746. https://doi.org/10.1101/gr.214270.116.
36. Aury JM, Istace B. Hapo-G, haplotype-aware polishing of genome assemblies with accurate reads. NAR Genom Bioinform 2021;3(2):lqab034. https://doi.org/10.1093/nargab/lqab034.
37. Wick RR, Holt KE. Polypolish: short-read polishing of long-read bacterial genome assemblies. PLoS Comput Biol 2022;18(1):e1009802. https://doi.org/10.1371/journal.pcbi.1009802.
38. Depthcharge v0.2.0. https://github.com/slimsuite/depthcharge. Accessed 28 January 2023.
39. Scaffhic v1.1. https://github.com/wtsi-hpag/scaffHiC. Accessed 7 December 2022.
40. Durand NC, Shamim MS, Machol I, et al. Juicer provides a one-click system for analyzing

loop-resolution Hi-C experiments. Cell Syst 2016;3(1):95-98. https://doi.org/10.1016/j.cels.2016.07.002.

41. Dudchenko O, Batra SS, Omer AD, et al. De novo assembly of the *Aedes aegypti* genome using Hi-C yields chromosome-length scaffolds. Science 2017;356(6333):92-95. https://doi.org/10.1126/science.aal3327.
42. Xu M, Guo L, Gu S, et al. TGS-GapCloser: A fast and accurate gap closer for large genomes with low coverage of error-prone long reads. Gigascience 2020;9(9):giaa094. https://doi.org/10.1093/gigascience/giaa094.
43. Leszek P. Pryszcz, Toni Gabaldón, Redundans: an assembly pipeline for highly heterozygous genomes, Nucleic Acids Res. 2016;44(12):e11. https://doi.org/10.1093/nar/gkw294.
44. Teloclip v0.0.3. https://github.com/Adamtaranto/teloclip. Accessed 28 March 2023.
45. Seppey M, Manni M, Zdobnov EM. BUSCO: Assessing genome assembly and annotation completeness. Methods Mol Biol 2019;1962:227-245. https://doi.org/10.1093/bioinformatics/btv351.
46. Rashid U, Wu C, Shiller J, et al. AssemblyQC: a Nextflow pipeline for reproducible reporting of assembly quality. Bioinformatics 2024;40(8):btae477. https://doi.org/10.1093/bioinformatics/btae477.
47. Zhang Y, Lu H-W, Ruan J. GAEP: a comprehensive genome assembly evaluating pipeline. J Genet Genomics 2023;50(10):747-754. https://doi.org/10.1016/j.jgg.2023.05.009.
48. Ou S, Chen J, Jiang N. Assessing genome assembly quality using the LTR Assembly Index (LAI), Nucleic Acids Res 2018; 46(21): e126. https://doi.org/10.1093/nar/gky730.
49. Rhie A, Walenz BP, Koren S, et al. Merqury: reference-free quality, completeness, and phasing assessment for genome assemblies. Genome Biol 2020;21:245. https://doi.org/10.1186/s13059-020-02134-9.
50. Ou S, Su W, Liao Y, et al. Benchmarking transposable element annotation methods for creation of a streamlined, comprehensive pipeline. Genome Biol 2019;20:275. https://doi.org/10.1186/s13059-019-1905-y.
51. Girgis HZ. Red: an intelligent, rapid, accurate tool for detecting repeats de-novo on the genomic scale. BMC Bioinform 2015;16(1):227. https://doi.org/10.1186/s12859-015-0654-5.

52. Quinlan AR, Hall IM. BEDTools: a flexible suite of utilities for comparing genomic features. Bioinformatics, 2010;26(6):841-842. https://doi.org/10.1093/bioinformatics/btq033.

53. Brůna T, Hoff KJ, Lomsadze A, et al. (2021) BRAKER2: automatic eukaryotic genome annotation with GeneMark-EP+ and AUGUSTUS supported by a protein database. NAR Genom Bioinform 2021;3(1):lqaa108. https://doi.org/10.1093/nargab/lqaa108.

54. Funannotate v1.8.16. https://github.com/nextgenusfs/funannotate. Accessed 12 March 2023.

55. Zhang H, Tanner Y, Huang L, et al. dbCAN2: a meta server for automated carbohydrate-active enzyme annotation. Nucleic Acids Res, 2018;46:W95-W101. https://doi.org/10.1093/nar/gky418.

56. Huerta-Cepas J, Forslund K, Coelho LP, et al. Fast genome-wide functional annotation through orthology assignment by eggNOG-mapper. Mol Biol Evol 2017;34:2115-2122. https://doi.org/10.1093/molbev/msx148.

57. The Gene Ontology Consortium. The gene ontology resource: 20 years and still GOing strong. Nucleic Acids Res 2019;47(D1):D330-D338. https://doi.org/10.1093/nar/gky1055.

58. Ashburner M, Ball CA, Blake JA, et al. Gene ontology: tool for the unification of biology. Nat Genet 2000;25:25-29. https://doi.org/10.1038/75556.

59. Kanehisa M, Soto Y, Kawashima M, et al. KEGG as a reference resource for gene and protein annotation. Nucleic Acids Res 2016;44(D1):D457-D462. https://doi.org/10.1093/nar/gkv1070.

60. Mitchell AL, Attwood TK, Babbitt PC, et al. InterPro in 2019: improving coverage, classification and access to protein sequence annotations. Nucleic Acids Res 2019;47(D1): D351-D360. https://doi.org/10.1093/nar/gky1100.

61. Rawlings ND, Barrett AJ, Thomas PD, et al. The merops database of proteolytic enzymes, their substrates and inhibitors in 2017 and a comparison with peptidases in the PANTHER database. Nucleic Acids Res 2018;46(D1):D624-D632. https://doi.org/10.1093/nar/gkx1134.

62. El-Gebali, S., Mistry, J., Bateman, A., et al. The Pfam protein families database in 2019. Nucleic Acids Res 2019;47(D1):D427-D432. https://doi.org/10.1093/nar/gky995.

63. Almagro Armenteros JJ, Tsirigos KD, Sønderby CK et al. SignalP 5.0 improves signal peptide predictions using deep neural networks. Nat Biotechnol 2019;37(4):420-423. https://doi.org/10.1038/s41587-019-0036-z.

64. The UniProt Consortium. UniProt: a worldwide hub of protein knowledge. Nucleic Acids Res 2019;47(D1):D506-D515. https://doi.org/10.1093/nar/gky1049.

65. Nevers Y, Vesztrocy AW, Rossier V, et al. Quality assessment of gene repertoire annotations with OMArk. Nat Biotechnol 2024. https://doi.org/10.1038/s41587-024-02147-w.

66. Fernandez CGT, Bayer PE, Petereit J, et al. The conservation of gene models can support genome annotation. The Plant Genome 2023;16(3):e20377. https://doi.org/10.1002/tpg2.20377.

67. Camacho C, Coulouris G, Avagyan V, et al. BLAST+: architecture and applications. BMC Bioinf 2009;10:421. https://doi.org/10.1186/1471-2105-10-421.

68. Emms DM, Kelly S. OrthoFinder: solving fundamental biases in whole genome comparisons dramatically improves orthogroup inference accuracy. Genome Biol 2015;16:157. https://doi.org/10.1186/s13059-015-0721-2.

69. Emms DM, Kelly S. OrthoFinder: phylogenetic orthology inference for comparative genomics. Genome Biol 2019;20:238. https://doi.org/10.1186/s13059-019-1832-y.

70. Emms DM, Kelly S. STAG: Species tree inference from all genes. bioRxiv 2018. https://doi.org/10.1101/267914.

71. Emms DM, Kelly S. STRIDE: species tree root inference from gene duplication events. Mol Biol Evol 2017;34:3267-3278. https://doi.org/10.1093/molbev/msx259.

72. Han MV, Thomas GWC, Jose LM, et al. Estimating gene gain and loss rates in the presence of error in genome assembly and annotation using cafe 3. Mol Biol Evol 2013;30(8):1987-1997. https://doi.org/10.1093/molbev/mst100.

73. dos Reis M, Zhu T, Yang Z. The impact of the rate prior on Bayesian estimation of divergence times with multiple loci. System Biol 2014;63:555-565. https://doi.org/10.1093/sysbio/syu020.

74. Chen CJ, Chen H, Zhang Y, et al. TBtools - an integrative toolkit developed for interactive analyses of big biological data. Mol Plant 2020;13(8):1194-1202. https://doi.org/10.1016/j.molp.2020.06.009.

75. Zwaenepoel A, de Peer YV. wgd-simple command line tools for the analysis of ancient whole-genome duplications. Bioinformatics 2019;35:2153-2155. https://doi.org/10.1093/bioinformatics/bty915.

76. Almeida-Silva F, Van de Peer Y. doubletrouble: Identification and classification of duplicated genes. R package version 0.99.1. 2022. https://github.com/almeidasilvaf/doubletrouble. Accessed 28 March 2023.
77. Qiao X, Li QH, Yin H, et al. Gene duplication and evolution in recurring polyploidization–diploidization cycles in plants. Genome Biol 2019;20:38. https://doi.org/10.1186/s13059-019-1650-2.
78. MCScanX. https://github.com/wyp1125/MCScanX. Accessed 28 July 2023.
79. Wang Y, Jia L, Tian G. et al. shinyCircos-V2.0: Leveraging the creation of Circos plot with enhanced usability and advanced features. iMeta 2023;2(2):e109. https://doi.org/10.1002/imt2.109.
80. Bandi V, Gutwin C. 2020. Interactive exploration of genomic conservation. In Proceedings of the 46th Graphics Interface Conference on Proceedings of Graphics Interface 2020 (GI'20). Canadian Human-Computer Communications Society, Waterloo, CAN.
81. Pérez-Wohlfeil E, Diaz-del-Pino S, Trelles O. Ultra-fast genome comparison for large-scale genomic experiments. Sci rep 2019;9:10274. https://doi.org/10.1038/s41598-019-46773-w.
82. Goel M, Schneeberger K. plotsr: visualizing structural similarities and rearrangements between multiple genomes, Bioinformatics 2022;38(10): 2922-2926. https://doi.org/10.1093/bioinformatics/btac196.
83. Shao Z-Q, Xue J-Y, Wu P, et al. Large-scale analyses of angiosperm Nucleotide-Binding Site-Leucine-Rich Repeat genes reveal three anciently diverged classes with distinct evolutionary patterns. Plant Physiol 2016;170(4):2095-2109. https://doi.org/10.1104/pp.15.01487.
84. Chou WC, Jha S, Linhoff MW et al. The NLR gene family: from discovery to present day. Nat Rev Immunol 2023;23,635-654. https://doi.org/10.1038/s41577-023-00849-x.
85. Santos MdL, Resende MLV, Alves GSC, et al. Genome-wide identification, characterization, and comparative analysis of NLR resistance genes in *Coffea* spp.. Front Plant Sci 2022;13:868581. https://doi.org/10.3389/fpls.2022.868581.
86. Liu Y, Zhang Y-M, Tang Y, et al. The evolution of plant NLR immune receptors and downstream signal components. Curr Opin Plant Biol 2023;73:102363. https://doi.org/10.1016/j.pbi.2023.102363.

87. De-la-Cruz IM, Hallab A, Olivares-Pinto U, et al. Genomic signatures of the evolution of defence against its natural enemies in the poisonous and medicinal plant *Datura stramonium* (Solanaceae). Sci Rep 2021;11:882. https://doi.org/10.1038/s41598-020-79194-1.
88. Tian F, Yang DC, Meng YQ, et al. PlantRegMap: charting functional regulatory maps in plants. Nucleic Acids Res 2019;48(D1):D1104-D1113. https://doi.org/10.1093/nar/gkz1020.
89. Puritz JB, Hollenbeck CM, Gold JR. Docent: a RADseq, variant-calling pipeline designed for population genomics of non-model organisms. PeerJ 2014;2:e431 https://doi.org/10.7717/peerj.431.
90. Liu H-L, Harris AJ, Wang Z-F, et al. The genome of the Paleogene relic tree *Bretschneidera sinensis*: insights into trade-offs in gene family evolution, demographic history, and adaptive SNPs. DNA Res 2022;29(1):dsac003. https://doi.org/10.1093/dnares/dsac003.
91. Wang Z-F. Genomic analysis through high throughput sequencing (in Chinese). Beijing: Science Press; 2022.
92. Tello D, Gil J, Loaiza CD, et al. NGSEP3: accurate variant calling across species and sequencing protocols. Bioinformatics 2019;35(22):4716-4723. https://doi.org/10.1093/bioinformatics/btz275.
93. Van der Auwera GA, O'Connor BD. Genomics in the Cloud: Using Docker, GATK, and WDL in Terra (1st Edition). O'Reilly Media. 2020.
94. Li H, Durbin R. Fast and accurate short read alignment with Burrows-Wheeler transform. Bioinformatics, 2009;25(14):1754-1760. https://doi.org/10.1093/bioinformatics/btp324.
95. Garrison E, Marth G. Haplotype-based variant detection from short-read sequencing. arXiv 2012:1207.3907. https://doi.org/10.48550/arXiv.1207.3907.
96. Hwang S, Kim E, Lee I. et al. Systematic comparison of variant calling pipelines using gold standard personal exome variants. Sci Rep 2015;5:17875. https://doi.org/10.1038/srep17875.
97. Wang J, Skoog T, Einarsdottir E. et al. Investigation of rare and low-frequency variants using high-throughput sequencing with pooled DNA samples. Sci Rep 2016;6:33256. https://doi.org/10.1038/srep33256.
98. Sandmann S, de Graaf A, Karimi M et al. Evaluating Variant Calling Tools for Non-Matched Next-Generation Sequencing Data. Sci Rep 2017;7:43169. https://doi.org/10.1038/srep43169.

99. Schilbert HM, Rempel A, Pucker B. Comparison of Read Mapping and Variant Calling Tools for the Analysis of Plant NGS Data. Plants (Basel) 2020; 9(4):439. https://doi.org/10.3390/plants9040439.

100. Yao Z, You FM, N'Diaye A, et al. Evaluation of variant calling tools for large plant genome re-sequencing. BMC Bioinform 2020;21:360. https://doi.org/10.1186/s12859-020-03704-1.

101. Musker SD, Ellis AG, Schlebusch SA, et al. Niche specificity influences gene flow across fine-scale habitat mosaics in Succulent Karoo plants. Mol Ecol 2021;30(1):175-192. https://doi.org/10.1111/mec.15721.

102. Stegemiller MR, Redden RR, Notter DR, et al. Using whole genome sequence to compare variant callers and breed differences of US sheep. Front Genet 2023;13:1060882. https://doi.org/10.3389/fgene.2022.1060882.

103. O'Fallon B, Bolia A, Durtschi J, et al. Generative haplotype prediction outperforms statistical methods for small variant detection in NGS Data. Bioinformatics 2024;btae565. https://doi.org/10.1093/bioinformatics/btae565.

104. Andreu-Sánchez S, Chen L, Wang D, et al. A Benchmark of Genetic Variant Calling Pipelines Using Metagenomic Short-Read Sequencing. Front Genet 2021;12:648229. https://doi.org/10.3389/fgene.2021.648229.

105. SNP Filtering Tutorial. http://www.ddocent.com/filtering/. Accessed on: 10 Jan 2021

106. Danecek P, Auton A, Abecasis G, et al. The variant call format and VCFtools. Bioinformatics. 2011;27(15):2156-2158. https://doi.org/10.1093/bioinformatics/btr330.

107. Purcell S, Chang C. PLINK. www.cog-genomics.org/plink/1.9/. Accessed on: 10 Jan 2021

108. Chang CC, Chow CC, Tellier LCAM, et al. Second-generation PLINK: rising to the challenge of larger and richer datasets. GigaScience 2015;4(1):7. https://doi.org/10.1186/s13742-015-0047-8.

109. Gaunt T, Rodríguez S, Day I. Cubic exact solutions for the estimation of pairwise haplotype frequencies: implications for linkage disequilibrium analyses and a web tool 'CubeX'. BMC Bioinform 2007;8:428. https://doi.org/10.1186/1471-2105-8-428.

110. Luu K, Bazin E, Blum MG. pcadapt: an R package to perform genome scans for selection based on principal component analysis, Mol Ecol Resour 2017;17:67-77.

https://doi.org/10.1111/1755-0998.12592.

111. Privé F, Luu K, Vilhja´lmsson BJ, et al. Performing highly efficient genome scans for local adaptation with R package pcadapt version 4. Mol Biol Evol 2020;37:2153–2154. https://doi.org/10.1093/molbev/msaa053.

112. Gautier M. Genome-wide scan for adaptive divergence and association with population-specific covariates. Genetics 2015;201:1555-1579. https://doi.org/10.1534/genetics.115.181453.

113. Korunes KL, Samuk K. pixy: Unbiased estimation of nucleotide diversity and divergence in the presence of missing data. Mol Ecol Resour 2021;21:1359-1368. https://doi.org/10.1111/1755-0998.13326.

114. Alexander DH, Novembre J, Lange K. Fast model-based estimation of ancestry in unrelated individuals. Genome Res 2009;19:1655-1664. https://doi.org/10.1101/gr.094052.109.

115. Zheng X, Levine D, Shen J, et al. A high-performance computing toolset for relatedness and principal component analysis of SNP data. Bioinformatics 2012;28(24):3326-3328. doi:10.1093/bioinformatics/bts606.

116. Mussmann S, Douglas MR, Chafin T, et al. AdmixPipe: population analyses in Admixture for non-model organisms, BMC Bioinform 2020;21:337. https://doi.org/10.1186/s12859-020-03701-4.

117. Kopelman NM, Mayzel J, et al. Clumpak: a program for identifying clustering modes and packaging population structure inferences across K. Mol Ecol Resour 2015;15:1179-1191. https://doi.org/10.1111/1755-0998.12387.

118. WFO (2024): *Ormosia macrocalyx* Ducke. Published on the Internet;http://www.worldfloraonline.org/taxon/wfo-0000168275. Accessed on: 10 Oct 2024

119. Bandel G. Chromosome numbers and evolution in the Leguminosae. Caryologia 1974;27(1): 17-32. https://doi.org/10.1080/00087114.1974.10796558.

120. Chen Y-L, Wang Z-F, Jian S-G, et al. Genome assembly of *Cordia subcordata*, a coastal protection species in Tropical Coral Islands. Int J Mol Sci 2023;24:16273. https://doi.org/10.3390/ijms242216273.

121. Mochizuki T, Sakamoto M, Tanizawa Y, et al. A practical assembly guideline for genomes with

various levels of heterozygosity. Brief Bioinform 2023;24(6):bbad337. https://doi.org/10.1093/bib/bbad337.

122. Rang FJ, Kloosterman WP, de Ridder J. From squiggle to basepair: computational approaches for improving nanopore sequencing read accuracy. Genome Biol 2018;19:90. https://doi.org/10.1186/s13059-018-1462-9.

123. Chen Y, Nie F, Xie SQ, et al. Efficient assembly of nanopore reads via highly accurate and intact error correction. Nat Commun 2021;12:60. https://doi.org/10.1038/s41467-020-20236-7.

124. Xiao H, Zhang Y, Wang M. Discovery and engineering of cytochrome P450s for terpenoid biosynthesis. Trends Biotechnol 2019;37:618-631. https://doi.org/10.1016/j.tibtech.2018.11.008.

125. Zheng X, Li P, Lu X. Research advances in cytochrome P450-catalysed pharmaceutical terpenoid biosynthesis in plants. J Exp Bot 2019;70:4619-4630. https://doi.org/10.1093/jxb/erz203.

126. Feulner PGD, De-Kayne R. Genome evolution, structural rearrangements and speciation. J Evol Biol 2017;30(8):1488-1490. https://doi.org/10.1111/jeb.13101.

127. Nascimento T, Pedrosa-Harand A. High rates of structural rearrangements have shaped the chromosome evolution in dysploid *Phaseolus* beans. Theor Appl Genet 2023;136:215. https://doi.org/10.1007/s00122-023-04462-3.

128. Ferguson S, Jones A, Murray K, et al. Plant genome evolution in the genus Eucalyptus is driven by structural rearrangements that promote sequence divergence. Genome Res 2024; 34(4):606-619. https://doi.org/10.1101/gr.277999.123.

129. Burssed B, Zamariolli M, Bellucco FT, et al. Mechanisms of structural chromosomal rearrangement formation. Mol Cytogenet 2022;15:23. https://doi.org/10.1186/s13039-022-00600-6

130. Hassan AH, Mokhtar MM, El Allali A . Transposable elements: multifunctional players in the plant genome. Front. Plant Sci. 2024;14:1330127. doi: https://doi.org/10.3389/fpls.2023.1330127.

131. Zhou C, Xia S, Wen Q, et al. Genetic structure of an endangered species *Ormosia henryi* in southern China, and implications for conservation. BMC Plant Biol 2023;23:220. https://doi.org/10.1186/s12870-023-04231-w.

132. Balloux F. Heterozygote excess in small populations and the heterozygote-excess effective population size. Evolution 2004;58(9):1891-900. https://doi.org/10.1111/j.0014-3820.2004.tb00477.x.

133. Stoeckel S, Grange J, Fernández-Manjarres JF, et al. Heterozygote excess in a self-incompatible and partially clonal forest tree species — *Prunus avium* L. Mol Ecol 2006; 15(8):2109-2118. https://doi.org/10.1111/j.1365-294X.2006.02926.x.

134. Stevens L, Salomon B, Sun G. (2007). Microsatellite variability and heterozygote excess in *Elymus trachycaulus* populations from British Columbia in Canada. Biochem Syst Ecol 2007;35(11):725-736. https://doi.org/10.1016/j.bse.2007.05.017.

135. Campoy JA, Lerigoleur-Balsemin E, Christmann H, et al. Genetic diversity, linkage disequilibrium, population structure and construction of a core collection of *Prunus avium* L. landraces and bred cultivars. BMC Plant Biol 2016;16:49. https://doi.org/10.1186/s12870-016-0712-9.

136. Ruiz Mondragon KY, Aguirre-Planter E, Gasca-Pineda J, et al. Conservation genomics of Agave tequilana Weber var. azul: low genetic differentiation and heterozygote excess in the tequila agave from Jalisco, Mexico. Peerj 2022;10:e14398. https://doi.org/10.7717/peerj.14398.

137. Cisternas-Fuentes A, Koski MH. Drivers of strong isolation and small effective population size at a leading range edge of a widespread plant. Heredity 2023;130:347-357. https://doi.org/10.1038/s41437-023-00610-z.

138. Depecker J, Verleysen L, Asimonyio JA, et al. Genetic diversity and structure in wild Robusta coffee (*Coffea canephora* A. Froehner) populations in Yangambi (DR Congo) and their relation to forest disturbance. Heredity 2023;130:145-153. https://doi.org/10.1038/s41437-022-00588-0.

139. Le Veve A, Burghgraeve N, Genete M, et al. Long-term balancing selection and the genetic load linked to the self-incompatibility locus in *Arabidopsis halleri* and *A. lyrata*. Mol Biol Evol 2023;40(6):msad120. https://doi.org/10.1093/molbev/msad120.

140. Jones P, Vogt T. Glycosyltransferases in secondary plant metabolism: tranquilizers and stimulant controllers. Planta 2001;213:164-174. https://doi.org/10.1007/s004250000492.

141. Keegstra K, Raikhel N. Plant glycosyltransferases. Curr Opin Plant Biol 2001;4(3):219-224. https://doi.org/10.1016/s1369-5266(00)00164-3.

142. Bowles D, Isayenkova J, Lim E-K, et al. Glycosyltransferases: managers of small molecules. Curr Opin Plant Biol 2005;8(3):254-263. https://doi.org/10.1016/j.pbi.2005.03.007.

143. Wang J, Hou B. Glycosyltransferases: key players involved in the modification of plant secondary metabolites. Front Biol China 2009;4:39-46. https://doi.org/10.1007/s11515-008-0111-1.

144. Gharabli H, Gala VD, Welner DH. The function of UDP-glycosyltransferases in plants and their possible use in crop protection. Biotechnol Adv 2023;67:108182. https://doi.org/10.1016/j.biotechadv.2023.108182.

145. Bolger AM, Lohse M, Usadel B. Trimmomatic: a flexible trimmer for Illumina sequence data, Bioinformatics 2014;30(15):2114–2120, https://doi.org/10.1093/bioinformatics/btu170.

Table 1. Six sampled (sub)populations and their genetic diversities in *Ormosia purpureiflora*

(Sub)population	Sample size	*Ho*	*He*	*Fis*	π
LFS1	26	0.877	0.460	-0.899	0.468
LFS2	26	0.872	0.458	-0.888	0.466
LFS3	26	0.867	0.460	-0.877	0.468
LFS4	26	0.882	0.457	-0.910	0.464
LFS5	26	0.854	0.449	-0.850	0.456
NKS	23	0.865	0.452	-0.874	0.460

Table 2. Statistics and evaluations of genome assemblies for three *Ormosia* species

Species	*O. purpureiflora*	*O. emarginata**	*O. semicastrata**
**Initial assembly statistic (bp)**			
N10	122,192,683	81,285,628	89,031,100
N20	120,000,233	63,464,384	79,796,434
N30	75,858,835	43,593,171	73,253,298
N40	61,354,201	37,463,220	56,807,054
N50	50,908,349	28,195,512	48,976,089
N60	45,450,924	25,800,464	45,239,136
N70	36,587,725	20,527,781	31,722,207
N80	15,728,371	13,438,452	22,051,163
N90	3,163,854	7,895,810	12,933,450
N100	34,487	173,104	128,272
Total length	1,811,176,403	1,420,917,605	1,511,766,959
Average length	5,786,506.08	15,787,973.39	23,996,300.94
Largest length	142,757,542	84,853,091	144,833,628
Minimum length	34,487	173,104	128,272
Number of contigs	313	90	63
**Assembly after applying Hi-C data (bp)**			
chr1	259,935,025	199,918,031	205,218,018
chr2	233,292,245	210,768,611	211,883,283
chr3	229,093,642	183,696,964	200,464,886
chr4	212,222,348	180,298,008	178,099,194
chr5	195,349,128	202,609,791	205,007,630
chr6	187,433,795	149,243,870	185,806,757
chr7	144,758,916	145,867,561	164,432,676
chr8	121,398,155	147,815,325	159,254,978
Unanchored to chromosome	645,468	35,505	519,897
Total length	1,584,128,722	1,420,253,666	1,510,687,319
**Assembly quality assessed by AssemblyQC**			
LAI	16.08	13.66	17.56
K-mer based assessment			
Completeness	88.36%	78.04%	81.15%
QV	28.83	27.02	28.34
**Assembly quality assessed by GAEP**			
GC content	35.06%	34.53%	34.63%
Mapping based assessment			
QV	39.74	37.38	38.46
Long WGS reads mapping ratio	97.32%[#]	99.72%[##]	99.59%[##]
Short WGS read mapping ratio[###]	99.76%	98.31%	98.43%
RNA-seq mapping ratio [####]			
Leaf	95.77%	93.52%	95.24%
Flower	91.15%	—	—
Fruit	95.96%	—	—
Seed	92.23%	—	—

*: From Liu et al. [15]; [#]: With reads longer than 20Kb; [##]: With reads longer than 10Kb; [###]: Trimmed and error-corrected; [####]: Trimmed using trimmomatic (RRID:SCR_011848) v0.39 [145] with parameter of “SLIDINGWINDOW:4:5 LEADING:5 TRAILING:5 MINLEN:25”.

Table 3. Statistics of predicted genes for three *Ormosia* species[$]

Species	*O. purpureiflora*	*O. emarginata*	*O. semicastrata*
Predicted gene information			
No. of protein-coding genes	55,061	50,517	51,220
No. of mRNAs	59,809	54,456	55,363
No. of exons	254,087	241,766	245,306
No. of CDSs	242,624	232,457	235,681
No. of five_prime_UTRs	32,588	25,531	27,197
No. of introns	193,432	186540	189213
No. of three_prime_UTRs	30,110	23,826	25,220
Genes			
Average gene length (bp)	2,991.06	3,277.60	3,356.76
Largest length of genes (bp)	423,361	494,687	267,325
Minimum length of genes (bp)	141	153	153
50% cumulative length of genes (bp)	1467	1636	1710
90% cumulative length of genes (bp)	7312	7422	7533
Exons in genes			
Average exons per gene	3.81	4.03	4.03
Average exon length (bp)	226.87	218.64	222.94
Largest length of exons (bp)	8728	7959	7959
Minimum length of exons (bp)	3	3	3
50% cumulative length of exons (bp)	159	149	151
90% cumulative length of exons (bp)	658	581	603
Introns in genes			
Average introns per gene	2.81	3.03	3.03
Average intro length (bp)	757.16	790.41	811.46
Largest length of introns (bp)	422,767	783,472	318,269
Minimum length of introns (bp)	11	11	11
50% cumulative length of introns (bp)	229	234	246
90% cumulative length of introns (bp)	1375	1419	1463
CDS in genes			
Average CDS length (bp)	864.15	881.45	898.37
Largest length of CDSs (bp)	16,359	15,351	16,323
Minimum length of CDSs (bp)	141	150	144
50% cumulative length of CDSs (bp)	609	621	642
90% cumulative length of CDSs (bp)	1941	1953	1917
Gene functional annotations using different databases			
dbCAN	1,671	1,538	1,596
EggNOG	41,143	38,192	38,955
KEGG	20,284	19,163	19,545
GO	29,006	27,167	27,824
InterPro	35,255	32,777	33,548
MEROPS	1,335	1,269	1,292
Pfam	28,007	26,150	27,091
SignalP	4,143	3,778	3,952
UniProt	9,517	8,988	9,234
Total	42,348	39,147	40,100

[$]: Using all transcripts

Figure legends

Figure 1 Picture of *Ormosia purpureiflora*. A) *O. purpureiflora* seeds. Seed sizes measured using a ruler are illustrated in the lower right panel; B) *O. purpureiflora* flowers; C) *O. purpureiflora* fruit in the distance showing the diseased state or insect invasion; D) *O. purpureiflora* seeds showing invasion by worms/insect or the diseased state; E) *O. purpureiflora* flowers in the diseased state or under insect invasion; F) *O. purpureiflora* natural habitat; the arrow shows the sampled individual (a small shrub) used for genome assembly.

Figure 2 Sampled (sub)populations and population genetics of *Ormosia purpureiflora*. A) Map showing (sub)populations sampled for *Ormosia purpureiflora*; B) Principal component analysis (PCA) results showing the first three PCs (PC1 vs. PC2 and PC1 vs. PC3) for *O. purpureiflora* individuals sampled from different (sub)populations; C) Admixture results representing data for K=2–6 clusters; D) Genetic diversity indices (π, *dxy*, *Ho*, *He* and *Fst*) and SNP density along the *O. purpureiflora* chromosomes (per 1 Mbp).

Figure 3 *Ormosia* genomes and comparative genomics. A) Hi-C interaction heat maps (bin length 100,000 bp) for the genome assemblies of three *Ormosia* species; B) Circos plot showing the genome features (chromosome, repeat density, gene density and syntenic blocks from outer to inner) across chromosomes of the genome assemblies of three *Ormosia* species. For *O. purpureiflora*, the Circos plot also includes SNP density results between the results of gene density and syntenic blocks. All densities were estimated using a 1-Mbp sliding window; C) The inferred phylogenetic tree, divergence time, and contracted (–) and expanded (+) gene families in *O. purpureiflora* and other species. D) The density distribution of synonymous nucleotide substitutions (*Ks*) in the whole genome duplication analysis for *Ormosia* species and their sister species, *L. albus*; E) Syntenic blocks among *Ormosia* species and *L. albus*; F) Intra-chromosomal structural variations observed among the three *Ormosia* species.

Figure 4 Smoothing lines for gene and repeat density distributions (Bin size: 100,000 bp) along chromosomes in *Ormosia* species. Scatterplots for the gene density and repeat distribution are presented in Supplementary Figure S4. Box sizes correspond to chromosome sizes in *Ormosia* species, whereas the black bars on the upper part of each box (chromosome) represent the hot structural rearrangement region in the chromosomes.

Additional files

Supplementary Table S1 Protein sequences of the species used for gene prediction.

Supplementary Table S2 Species used for comparative genomics.

Supplementary Table S3 Species pairs and their estimated divergence times used for time calibration points to infer time-calibrated phylogeny of *Ormosia purpureiflora*.

Supplementary Table S4 nQuire results for the ploidy level assessment in *Ormosia* species.

Supplementary Table S5 Repeat contents in *Ormosia* assemblies.

Supplementary Table S6 Summary of gene functional annotations of the *Ormosia purpureiflora* assembly performed using different databases.

Supplementary Table S7 OMArk gene quality assessment.

Supplementary Table S8 Number of the genes matching to representative genes in Fabaceae.

Supplementary Table S9 Comparison of gene number in part of InterPro accesssion among three *Ormosia* species.

Supplementary Table S10 Summary of gene families.

Supplementary Table S11 GO enrichment results for specific gene families in *Ormosia purpureiflora*.

Supplementary Table S12 KEGG enrichment results for specific gene families in *Ormosia purpureiflora*.

Supplementary Table S13 GO enrichment results for significantly expanded gene families in *Ormosia purpureiflora*.

Supplementary Table S14 KEGG enrichment results for significantly expanded gene families in *Ormosia purpureiflora*.

Supplementary Table S15 GO enrichment results for siginficantly contracted gene families in *Ormosia purpureiflora*.

Supplementary Table S16 KEGG enrichment results for significantly contracted gene families in *Ormosia purpureiflora*.

Supplementary Table S17 Number of different gene duplication in *Ormosia*.

Supplementary Table S18 GO enrichment results for *Ormosia purpureiflora* WGD genes.

Supplementary Table S19 KEGG enrichment results for *Ormosia purpureiflora* WGD genes.

Supplementary Table S20 GO enrichment results for tandem-duplicated genes in *Ormosia purpureiflora*.

Supplementary Table S21 KEGG enrichment results for tandem-duplicated genes in *Ormosia purpureiflora*.

Supplementary Table S22 GO enrichment results for proximal-duplicated genes in *Ormosia purpureiflora*.

Supplementary Table S23 KEGG enrichment results for proximal-duplicated genes in *Ormosia purpureiflora*.

Supplementary Table S24 Syntenic block analysis results for *Ormosia* species.

Supplementary Table S25 Structural variations between the *Ormosia* species.

Supplementary Table S26 GO enrichment results for *Ormosia purpureiflora* specific inversion on Chromosome 2.

Supplementary Table S27 GO enrichment results for possible *Copia*-mediated genes in *Ormosia purpureiflora*.

Supplementary Table S28 KEGG enrichment results for possible *Copia*-mediated genes in *Ormosia purpureiflora*.

Supplementary Table S29 *R* genes in the species.

Supplementary Table S30 Summary of transcription factor genes in *Ormosia* species relative to those in the other species.

Supplementary Table S31 KEGG enrichment results for *Ormosia purpureiflora* genes on Chromosome 5 presumably under balancing selection.

Supplementary Figure S1 Schematic showing SNP calling.

Supplementary Figure S2 Chromosome numbers observed in *Ormosia purpureiflora* (scale bar: 10 μm)

Supplementary Figure S3 Genome size estimation using GenomeScope.

Supplementary Figure S4 Gene and repeat density (Bin size: 100,000 bp) along chromosomes in *Ormosia* species. The smoothing line is added for each density distribution by the geom_smooth() function in the ggplot2 program of the R package. Box sizes correspond to chromosome sizes in the *Ormosia* species.

Supplementary Figure S5 Dot plots among three *Ormosia* species. The red arrow indicates a specific inversion on Chromosome 2 of *O. purpureiflora*.

Supplementary Figure S6 Distribution of *R* genes on each chromosome in the three *Ormosia* species.

Supplementary Figure S7 Scree plot from PCAdapt.

Supplementary Figure S8 Cross validation error plot of admixture analysis.

Figure 1

Click here to access/download;Figure;Figure_1_resized.tif

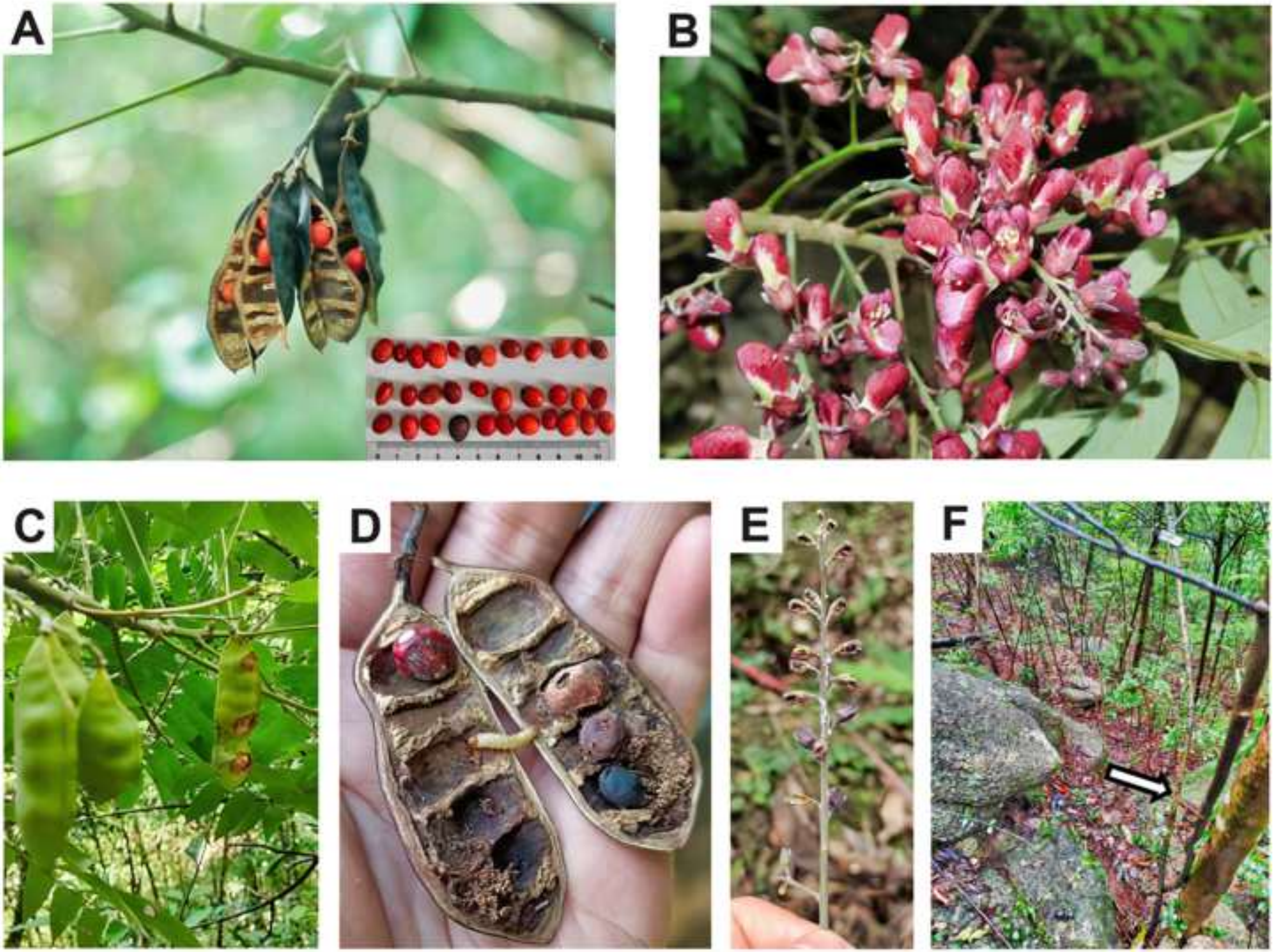

Figure 2

Click here to access/download;Figure;Figure_2_resized.tif

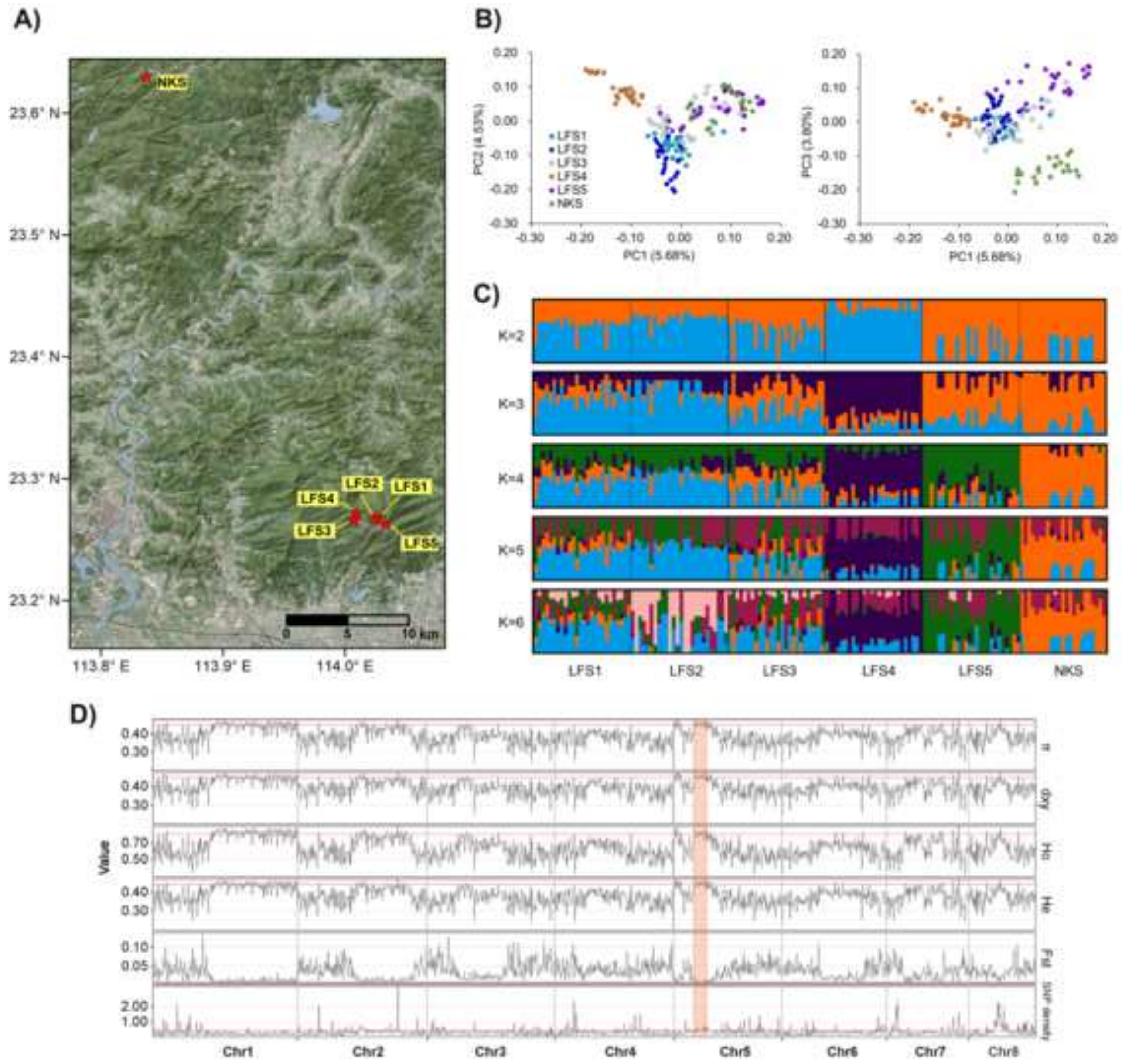

Figure 3

Click here to access/download;Figure;Figure_3_resized.tif

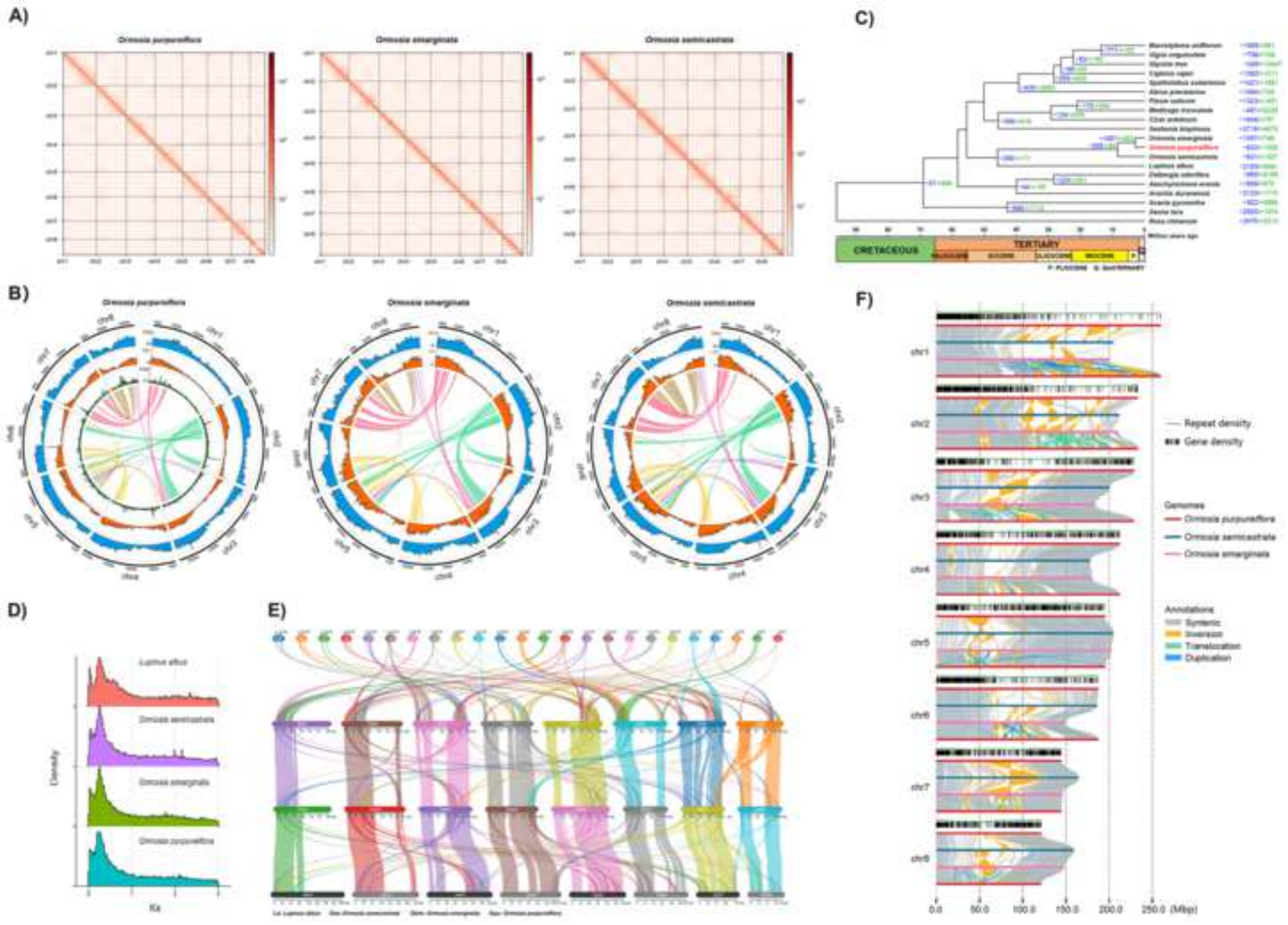

Click here to access/download
Supplementary Material
Figure_4_resized.tif

Supplementary Figure S1

Click here to access/download
Supplementary Material
Figure_S1.tif

Supplementary Figure S2

Click here to access/download
Supplementary Material
Figure_S2.tiff

Supplementary Figure S3

Click here to access/download

Supplementary Material

Figure_S3.png

Supplementary Figure S4

Click here to access/download
Supplementary Material
Figure_S4.tif

Supplementary Figure S5

Click here to access/download
Supplementary Material
Figure_S5.tif

Supplementary Figure S7

Click here to access/download
Supplementary Material
Figure_S7.tif

Supplementary Figure S8

Click here to access/download
Supplementary Material
Figure_S8.tif

Supplementary Tables

Click here to access/download
Supplementary Material
supplymentary_table_revised.xlsx

Manuscript revised-tracked

Click here to access/download
Supplementary Material
MS-revised-tracked.docx
