# Supplementary material for: Chromosome-scale assemblies of three Ormosia species: repetitive sequences distribution and structural rearrangement
Source: Gigascience. 2025 May 16;14:giaf047. doi: 10.1093/gigascience/giaf047 (PMC12083454; doi:10.1093/gigascience/giaf047)
Supplement: giaf047_Authors_Response_To_Reviewer_Comments_Original_Submission [file giaf047_authors_response_to_reviewer_comments_original_submission.pdf]

Author's Response To Reviewer Comments

GigaScience
December 12, 2024

Dear editor,
Thank you very much for your decision letter about our manuscript entitled "Chromosome-scale assemblies of three Ormosia species: Gene-repeat association architecture, structural rearrangement, and balancing selection" (No. GIGA-D-24-00350), including the comments.
We are now sending our responses to comments with our revised manuscript (both tracked and clean versions). All the modified parts in the revised manuscript are marked in red.
Our specific responses are as follows:

Comments from the reviewers:
Reviewer #1:
1. There is very little context about important information of the sequenced species such as the ploidy and the reproductive mode (sexual reproduction, autogamous or allogamous, etc). This information is critical to know the expected assembly that should be pursued. The analysis of the genomic variation dataset should include an analysis of heterozygosity per sample to know which type of assembly should be generated. In case that a phased assembly would be appropriate, I assume that this could not be made due to the error rate of nanopore reads. This error rate should be reported and the reason for not trying a phased assembly must be clearly stated in the manuscript.
>>>1) We have included information on the ploidy and reproductive mode, as suggested. Please refer to lines 87–90, 187–188, 372–376, and Table S4.
2) We have included information on the heterozygosity for Ormosia purpureiflora and the other two Ormosia species. Please refer to lines 185–187, 390–391, and 397–399.
3) We have incorporated the error rate estimation for Nanopore reads and additional details addressing the phased assembly. Please refer to lines 146–147 and 431–439.

2. The information related to the origin of the RNA-seq data is unclear. The text seems to indicate that RNA-seq was collected from 153 accessions, but then the SRA records suggest that only one accession was sequenced. More importantly, the experimental design did not seem to include biological replicates. This makes the results of differential expression meaningless. Taking into account that the manuscript is a data note, I recommend to completely remove the section "RNA-seq analysis of tissues". The RNA-seq data generated in this work is worth to aid the gene annotation process, but the experiment is not correctly designed to assess differential expression. As a small note, nothing is "highly significant". Significance is an assessment of probability that an event (a difference in many cases) is real. Hence, it should not be a term subject to gradation.
>>>We performed RNA-seq of only one O. purpureiflora individual, which was used for genome annotation. The other 153 samples underwent only whole-genome resequencing and were not subjected to RNA-seq. We have revised the text to clarify this point. Please refer to lines 153–155. In addition, we have removed references to "RNA-seq analysis of tissues" throughout the text and corrected the incorrect use of the term "highly significant," as suggested.

3. Likewise, the protocol used to obtain DNA sequencing data for the diversity panel is unclear. Regarding the samples, it is not clear if the sampling is representative for the species. Regarding the data, it is not clear which protocol was used for sequencing. The text suggests that it was WGS, but the number of reported SNPs is very low for a WGS dataset of a plant species. The methods indicate that SNPs were called with a pipeline called "dDocent". Looking at the documentation, this seems to be a pipeline for RAD-sequencing data, which probably means that it is not a good option for WGS data. This could be the main reason explaining why the number of SNPs is very small for a WGS dataset. This could also explain the reported result regarding excess of heterozygosity. If the authors want to release the resequencing data an a database of genomic variation, I recommend using more standard variant calling and genotyping tools such as NGSEP or GATK and redo the downstream analysis using the possibly more than 1 million SNPs that should be obtained from these data.

>>>1) We have clarified the sampling information in the text; please refer to lines 84–87 and 148–151. The sampling strategy was based on a thorough field investigation. Each (sub)population was randomly sampled, ensuring that sampling areas covered the entire region of each (sub)population. Sampling sizes ranged from 23 to 26 individuals per (sub)population, which are appropriate for population genetics studies.
2) The SNP-calling pipeline, dDocent (which uses Freebayes for SNP calling), is compatible with both RAD-seq and whole-genome resequencing data (please see https://www.ddocent.com/). We have clarified this point in the text; please refer to lines 311–318. Similarly, NGSEP can handle various NGS data types, including RAD-seq data (https://github.com/NGSEP/NGSEPcore).
We conducted whole-genome resequencing for each sample, and the initial SNP calling using Freebayes in the dDocent pipeline yielded 238,531,000 SNPs. However, only a small proportion of SNPs remained after filtering for low quality, Hardy-Weinberg equilibrium (HWE), and other parameters. Filtering out low-quality SNPs ensures accurate genetic diversity and structure analyses.
Additionally, we included NGSEP and GATK results in our study. However, due to the significant computational time required for GATK, we used it only to validate dDocent and NGSEP results, rather than for whole-genome SNP calling. Genetic diversity and structure analyses were then conducted using SNPs identified by all three tools. We have added a detailed explanation and schematic of this process in Supplementary Figure S1 and the "Single-nucleotide polymorphism calling" section in "Methods". Accordingly, we re-analyzed the SNP results. Please see sections of "Single-nucleotide polymorphism calling" and "Genetic diversity and genetic structure" in "Results".
3) The low number of SNPs does not affect the observed excess heterozygosity. The retained SNPs adequately reflect population genetic processes because they are neutral and randomly distributed across chromosomes. In our current research on another species (Dunnia sinensis, unpublished), we identified 144,746 SNPs (including 112,324 bi-allelic SNPs) after filtering. Using these SNPs, we found two populations with heterozygosity deficits (inbreeding coefficient Fis values of 0.9889 and 0.9895), whereas the other four populations exhibited excess heterozygosity (Fis values ranged from −0.6475 to −0.7100). In O. purpureiflora, excess heterozygosity may be explained by outcrossing and clonal reproduction. We have included an explanation for this in the text; please refer to lines 659–669.

4. About the analysis of genetic structure, if the number of sampled populations is actually 6, it does not make sense to show the results only until k=4. The resolution of figure 2 needs to be improved. I can not differentiate correctly the populations in the current PCA. If the most genetically distant population is NKS, it is weird that this population gets differentiated only until k=4. Again k=5 and k=6 are needed to evaluate the stability of this differentiation.
>>>After submission, the journal reduced the resolution of the figures to minimize the manuscript size. However, high-resolution figures can be accessed by clicking on and downloading them for review. Additionally, we have included the result for K = 6 and revised the text accordingly. Please refer to Figure 2C and lines 686–690.

Minor comments.
1. The order of the figures looks disorganized relative to the order of the text. The reason for this seems to be that the map in figure 2A is cited in the context to talk about the distribution of the species. Given that this map does not actually show much of global context, I would recommend eliminating the references to figure 2A in the context and in the methods and reorganize the figures.
>>>We have deleted the citation of Figure 2A in the Introduction, as suggested.

2. The resolution of the figures 2 and 3 is very poor. This makes it difficult to follow the results included in these figures.
>>>After submission, the journal reduced the resolution of the figures to minimize the manuscript size. However, high-resolution figures can be accessed by clicking on and downloading them for review.

3. Abstract: Please rephrase the second sentence of the background
>>>We have rephrased the texts in the background, as suggested. Please refer to lines 32–34.

4. Line 40: it is not clear what "substantial" means. It is also not clear to what Ormosia is compared in the expression "...fewer transcription factor genes"
>>>We have rephrased the statement for clarity. Please refer to lines 45 – 46.

5. Line 135. Replace "inputed into" with "given to"
>>>We have replaced the term “inputed into” and rephrased the sentence. Please refer to lines 186.

6. Lines 331 - 342. The text looks speculative and I think it is not needed for a data note.
>>>We have removed these sentences as suggested.

7. Lines 352-363. Again, I think that a data note does not require speculation about possible consequences of the genes that look missing in the assemblies.
>>>We have removed these sentences as suggested.

8. Line 392. The expression "a recent ancient WGD" looks self contradictory. More importantly, the event does not seem to be genus specific, but it just seem to be the known event in fabales. The authors could simply refer to this event.
>>>We have corrected these contradictory sentences. Please refer to line 543. For WGD, we have included the required information as suggested. Please refer to lines 544–546.

9. Line 399. It is not clear what are "TD-type genes". I assume they would be the tandem duplication, but this needs to be clear in the text.
>>>We have rephrased the words for better clarity. Please see line 553.

10. Line 401. Likewise, it is not clear what are "PD-type genes".
>>>We have rephrased the words for better clarity. Please see line 555.

11. Line 541-548. Again the text looks speculative. I recommend to remove this paragraph.
>>>We have removed these sentences, as suggested.

12. Lines 550-561. Again I think that this analysis should not be included in a data note, especially because the results do not seem to be conclusive.
>>>We have removed these sentences, as suggested.

Reviewer #2:
General comment: The current state of written English in the manuscript requires attention. A comprehensive review of the English language including sentence structure and overall flow is highly recommended to enhance the quality of the manuscript.
>>>We have used the editing service of an English language editing company, TopEdit (www.topeditsci.com), to improve quality of our manuscript. The English revised parts are marked in red in the text.

Line 40: This is not a very informative sentence to highlight the main findings. "substantial" and "fewer" are relative terms, thus substantial R genes and fewer TFs does not reveal the true extent/number of these identified genomic regions. please reframe.
>>>We have rephrased the sentences. Please refer to lines 45–46.

Line 42: what does it mean by "missed"? missed in the analysis or absent in the species?
>>> We have replaced “missed” with “absent.” Please refer to line 48.

It would be helpful to provide an account of the current status of genomic studies in the genus and may be closely related species.
>>>We have included information on the genomic studies conducted in the genus so far. Please refer to lines 101–112.

Line 102: Is the storage temperature for root tips indicated here, correct? Were the root tips incubated at subzero temperature?
>>>We have rephrased the sentences for clarity. Please refer to lines 126–128.

Figure 1A: Karyotype picture its loosing its relevance in the inset. Please consider reordering it.

>>>We have redrawn the picture and included the karyotype result as a separate supplementary file, Figure S2.

Lines 108-127: The authors have not provided details regarding the preparation of the DNA/RNA samples and sequencing libraries, which is a critical omission. Given that the study focuses on a less-studied plant species that is not well-characterized, this information is especially important for ensuring the reproducibility of the results and aiding future studies. I highly recommend including a clear description of the library preparation methods.
>>>The details of sequencing information, including DNA/RNA preparation and library construction (except Nanopore ultra-long sequencing), have been provided in our previous studies. We have cited our previous studies and added details for Nanopore ultra-long sequencing. Please refer to lines 162–176.

It is unclear whether biological replicates were employed in the RNA sequencing. Please include these details.
>>>We did not use replicates and thus have removed RNA expression analysis as suggested by the other reviewer.

Line 129-138: Although, authors have reported the followed pipeline and softwares used for read processing and genome assembly, it is important to include details on the parameters used for each employed software. This is universally applicable to all the analysis softwares mentioned throughout the different portions of the study. These parameters should be fully described.
>>>We have used the default setting for programs during analyses. For additional parameters, we have described the detailed information in the revised manuscript. Please refer to lines 177–178.

Line 166-167: It is not clear how the redundancy in repeats identified through two different programs, EDTA and RED were taken care of.
>>>In combination, both overlapped and nonoverlapped repetitive regions of EDTA and RED were merged to form overall repetitive regions in the assembly. We have revised the information for better clarity. Please refer to lines 226–230.

Line 219-229: Identification of R genes has not been described.
>>>We have included the description in the text. Please refer to lines 295–300.

Line 291-293: I was expecting to see some information about how application of HiC has improved the previous reported assemblies of O. emarginata and O. semicastrata.
>>>We have included this information as suggested. Please refer to lines 396–397, 400–404, and Table 2.

Line 294-299: BUSCO indicates completeness of genic regions of the assemblies which are often not the most difficult genome regions to map and assemble. Authors should evaluate the integrity and completeness of the assembly through other metrics. My suggestions would be performing analysis of LTR assembly index (https://academic.oup.com/nar/article/46/21/e126/5068908?login=false), mapping back the short and long-read DNA/RNA sequences onto to assembly to correlate mapping percentages with genome continuity.
>>>As suggested, we have included more quality evaluations in the assemblies and replaced Table 2. Please refer to lines 211–216, 413–421, and Table 2.

Line 312-316: What is the length distribution of the genes predicted in the analysis. Have an exploratory analysis done to ascertain the quality of the predicted genes? How does the authors justify only 70% of their predicted genes being annotated.
>>>1) We have updated Table 3 to include the length distribution information. Please refer to Table 3, lines 455-457.
2) To assess gene quality in our studied species, we have included analyses using BUSCO, OMArk, and Fabaceae-represented gene matches. Please see lines 244–254 and 468–494.
3) We compared our annotation rates with those of the other genomes. Annotation rates vary across species, with similarly low rates observed in some cases. This could be attributed to the presence of unknown or novel genes. We have incorporated this information in the text; please refer to lines 460–467.

Line 327: "By also removing possible LTR-mediated genes and reciprocally excluding Helitron- or TIR-mediated genes, we finally retained 7216 possible Helitron-mediated genes and 2728 possible TIR-mediated genes." Does the author means LTRs were nested in the helitron and TIR repeats? and by removing these putative regions 7216 genes were retained from 21641? This statement is not clear.
>>>We have removed the whole paragraph as suggested by the other reviewer.

Line 361: This statement sounds very speculative. have the authors done any validation to make sure these genes are entirely missing from the assembly or have left undetected by the used pipeline?
>>>We have removed the whole paragraph as suggested by the other reviewer. These genes were not annotated in O. purpureiflora when gene annotation was performed using the InterPro database.

Table 2: Authors should consider elaborating the annotation details for the assemblies in the main Table which bring more visibility to the reader.
>>>We have replaced Table 2 and included this information as suggested. Please see Table 2 and Table 3, Table S6.

The manuscript does not describe and provide information about data validation and quality control.
>>>We have included this information as suggested, including additional information on experiments, genome assembly, genome and predicted gene quality evaluations, and SNP quality control. Please see above responses from two reviewers. In addition, all raw sequencing data are available in GenBank (please see “Data Availability”) and the results are available in Figshare (https://doi.org/10.6084/m9.figshare.26826466.v14). The results of synteny and the other comparative genomics among three Ormosia species confirmed their sequencing data are reliable.

Miscellaneous:
1) Because there are references added and removed, the order of references in the revised manuscript are changed. Please see revised citing numbers corresponding to the reference list in the text and “References” section.
2) Because revision, we also remove and replace the tables/figures in the text and supplementary materials.
3) We reanalyze some of the results as suggested, and the results are revised correspondingly.

Please let us know if there are any additional concerns about our manuscript after we have offered these corrections and responses, as we are happy to address any continued issues or proposed changes to the manuscript. Thank you for your time.

Sincerely yours,

Zheng-Feng Wang
South China Botanical Garden
Chinese Academy of Sciences
Guangzhou, 510650
China

Cookie Preference Center

We use cookies which are necessary to make our site work. We may also use additional cookies to analyze, improve and personalize our content and your digital experience. For more information, see our Cookie Policy.

You may choose not to allow some types of cookies. However, blocking some types may impact your experience of our site and the services we are able to offer. See the different category headings below to find out more or change your settings.

Allow all

Manage Consent Preferences

Strictly Necessary Cookies

Always active

These cookies are necessary for the website to function and cannot be switched off in our systems. They are usually only set in response to actions made by you which amount to a request for services, such as setting your privacy preferences, logging in or filling in forms. You can set your browser to block or alert you about these cookies, but some parts of the site will not then work. These cookies do not store any personally identifiable information.

Functional Cookies

These cookies enable the website to provide enhanced functionality and personalisation. They may be set by us or by third party providers whose services we have added to our pages. If you do not allow these cookies then some or all of these services may not function properly.

Performance Cookies

These cookies allow us to count visits and traffic sources so we can measure and improve the performance of our site. They help us to know which pages are the most and least popular and see how visitors move around the site.

Targeting Cookies

These cookies may be set through our site by our advertising partners. They may be used by those companies to build a profile of your interests and show you relevant adverts on other sites. If you do not allow these cookies, you will experience less targeted advertising.

Cookie List

Clear

Apply Cancel
Consent Leg.Interest

Confirm my choices

Powered by onetrust
