# Supplementary material for: Chromosome-scale assemblies of three Ormosia species: repetitive sequences distribution and structural rearrangement
Source: Gigascience. 2025 May 16;14:giaf047. doi: 10.1093/gigascience/giaf047 (PMC12083454; doi:10.1093/gigascience/giaf047)
Supplement: giaf047_Authors_Response_To_Reviewer_Comments_Revision_1 [file giaf047_authors_response_to_reviewer_comments_revision_1.pdf]

Author's Response To Reviewer Comments

Dear editor,
Thank you very much for your decision letter about our manuscript entitled “Chromosome-scale assemblies of three Ormosia species: Gene-repeat association architecture, structural rearrangement, and balancing selection” (No. GIGA-D-24-00350R1), including the comments.
We are now sending our responses to comments with our revised manuscript (both tracked and clean versions). All the modified parts in the revised manuscript are marked in red.
Our specific responses are as follows:

Comments from the reviewers:
Reviewer #1:
The manuscript describes the chromosome-level genome assembly and annotation of three new genomes, as well as WGS population genomics data for 6 populations of O. purpureiflora. I appreciate the effort taken by the authors to address properly most of my previous concerns. I think that the resource provided by the authors will be of great value for research in Fabales. However, I still have a some concerns related to the analysis of repetitive elements and the SNPs chosen for population genomics. These are my detailed comments:
1. I think that there is an issue with the analysis of repeat density, leading to a wrong interpretation of the result. It is inconsistent to state that 75% percent of the genome is composed of repetitive elements to later say that most pericentromeric regions have low repeat density. If the latter statement would be correct, one question that would need to be addressed is which kind of DNA is present in those regions (if it is not repeats or genes). Also, to the best of my knowledge, this would be the first report in the plant kingdom (and possibly in the whole tree of life) in which gene density has positive correlation with repeat density.
My guess is that the error lies in the definition of density used by the authors. Density is usually not measured in number of annotations, but in the percentage of the genome covered by such annotations. Based on the results reported in the text and shown in the supplementary figure 4 and supplementary table 5, my guess is that there is an overcounting of annotations of very small TIR and Helitron elements, which may be located close to genes, but that only contribute to about 4% of the genome. In contrast, the large LTR elements have numbers comparable to TIRs, but take 62% of the genome, probably spanning most pericentromeric regions, as suggested in the supplementary figure 4. The circos plots in figure 3 need to be corrected showing density as percentage of the genome covered. This probably will provide a more clear and correct interpretation of the distribution of repetitive elements across the genome.
>>>We agree with the reviewer. We have included repeat densities measured by their length proportions on the chromosomes and rephrased the corresponding sentences in the context. Please see lines 42-46, 222-225, 434-439, 1046, 1048, 1059-1060, 1116 and Fig. 3B.

2. The second major concern is related to the SNP filtering procedure and its consequence on the genotype calling of the selected SNPs. The authors report a surprisingly high (> 0.85) observed average heterozygosity (supplementary table 1). This means that on average more that 85% of genotype calls for each SNP are heterozygous. Probably this is also true for each individual. This is highly unusual for diploid organisms, and it will make nearly impossible to cluster individuals in populations. This can not be explained just by outcrossing or clonal reproduction. The only species in which I have seen such genome wide heterozygosities is the decaploid sugar cane. If this would be the case, then allelic dosages need to be calculated for each datapoint to obtain a dataset useful to make genetic clustering. This result also suggest that the HWE filter was not applied or it was not effective because the maximum expected heterezygosity in diploid individuals is 0.5 and a SNP genotyped in more than 100 individuals with OH>0.85 for sure will fail HWE. The only possible biological explanation that I can figure out would be that the population is just 153 genetic clones and then most SNPs are just the heterozygous sites of only one true genetic individual in the population.
Again, I think that the problem is technical and it can be easily solved. The most likely explanation for the current outcome is that several SNPs are located in regions with transposable elements, for which no analysis pipeline for Illumina reads can provide reliable genotyping. The most important filter to apply in this case is to remove SNPs within annotated transposable elements. The resulting SNPs will not be evenly

distributed in the genome, but they will be enriched in single copy (and gene rich) regions. However, genotype calls will accurate and hence the SNPs will be more reliable for sample clustering. Genome-wide low heterozygosity can easily be explained by inbreeding, but genome-wide high heterozygosity is very rare, especially in entire populations.
One more time I think it is worth to calculate and present the heterozygosity per individual (this is different than the OH). Please also calculate and show the distribution of Minor allele frequency, global and per population. Both distributions can be calculated with the summary statistics of NGSEP or with VCFTools. Interpreting these distributions helps to identify technical issues and to assess the effect of population structure in allele frequencies and diversity.
>>>The problem occurs at the step of HWE filtering. We have re-calculated the results. New results show that the Ho, He and Fis values range from 0.146-0.196, 0.122-0.154, -0.268-0.058, respectively. According to the new results, we have revised the manuscript title and rephrased the sentences accordingly. Please see lines 52-53, 312-313, 320-324, 600-617, 629-647, title, Table 1, Fig. 2B-D and Fig. 3B.

3. It is not clear which kind of selection was tested or to which environment refers the result regarding the SNPs predicted as "adaptive".
>>>In the selection analysis, we only detected the “outlier” of SNPs, and no environmental variants were included. We have rephrased them as “outlier”, and do not refer them as “adaptive”. Please see lines 57, 328, 337-338, 603-605.

Minor comments:
1. I could not find Figure 4 in the revised version
>>>We wrongly uploaded Figure 4 as supplementary file last time. We have corrected that.

2. Line 64. The word "their" should not be capitalized
>>>We have corrected that. Please see line 67.

3. Line 88. It should read: "… and it is primarily …"
>>>We have corrected that. Please see line 88.

4. Lines 108-112. I do not think it is needed to list every single new genome assembly in literature and it is definitely not mandatory for this paper to include in the analysis all genomes sequenced to date. If that would be the case, then I think that, for example, several new genomes of Phaseolus and Vigna species are missing. Talking about future work in the introduction is strange. I would just remove these sentences, but if needed, they may better go to the conclusions.
>>>We have remove these contents as suggested.

5. Lines 226-230. The sentence is confusing. Please take some more time and space to explain the procedure to merge the calls of transposable elements.
>>>We remove the sentences that did not state clearly, and have included a figure to illustrate the procedure. Please see lines 221-222 and Supplementary Fig. S1.

6. Lines 316-318. I would eliminate these sentences. The goal of the paper is not to defend a particular pipeline. The cited literature does not help to make the point. Most papers cited are very old for the field, some do not use good measures of quality for benchmarking of variant callers (Yao et al., 2020 for example) and even in Schilbert et al., 2020 the reported performance for Freebayes is among the worst (See table 1). Even in this paper, the raw number of SNPs given by Freebayes means that there is a SNP every 5bp, which is unrealistically high. Leaving to the filtering step the task to achieve good quality data is not a good attibute for a variant caller.
>>>We agree with the reviewer. We abandon both the results by Freebayes and GATK, and only use the SNPs called by NGSEP. Please see lines 312-314.

7. Lines 330-339. I am not sure what would be the effect of running the recalling step of GATK on VCFs generated using other tools, but my guess is that it may not be a good idea. Different tools have different models to estimate genotype likelihoods and even report those values using different optional fields. For this reason it is very likely that the GATK genotype recalling would only do fine for VCFs generated by

GATK. This could be another technical reason for the inflated observed heterozygosity. I would calculate summary statistics before and after this step to see if they actually produce an important change in the genotype calls (and in which direction). Rather than that, it is not mandatory to include steps of the GATK pipeline if this tool could not be used for computational efficiency reasons.
>>>We agree with the reviewer. We only use the SNPs called by NGSEP. Please see lines 312-314.

8. Line 495. As a side note to the concern 1, the authors should describe the procedure to calculate correlation and run a significance test for correlation.
>>>We have wrongly interpreted the associations between gene and repeat density previously, and removed the sentences. We include the repeat density in length proportion as described in Major Comment 1 above, and rephrased the sentences accordingly. Please see lines 434-439.

9. Line 543. Again, I would not call "recent" a WGD of more than 50 million years.
>>>We have corrected and rephrased the sentence. Please see line 522.

10. Lines 594 and 595. Please describe which measure of correlation was used and which statistical test was applied to determine strong correlation. Moreover, the sentence says that the rearrangements colocate with tegions of high repeat density, but the circos figures (3B) suggest that the regions with rearrangements have low repeat density.
>>>We have removed these contents which are not stated clearly. Previously, we only presented the repeat density in their numbers. Repeat densities in “Circos figures (3B)” were the results of total numbers of different repetitive elements. Fig. 4 presented the density distribution of each element in numbers. They were not stated clearly and caused confusion.
We now remove the sentences and include the repeat density results in both length proportions (percentage of length coverage on the chromosome) and numbers, and rephrase the sentences to make the statement clear. Please also see answer for Major Comment 1.

11. Line 606 and all other instances of the word "significant". Please describe the statistical test and report the p-value to determine in this case that rearrangements are a significant driving force of evolution.
>>> We have removed the paragraph which are not stated clearly.

12. Lines 615 and 625. Please clarify the meaning of the terms "other species" and "all the other species". A high number of predicted LRR genes and/or TFs can just be caused by false positive calls.
>>>We have included words to make the statement clearly. Please see lines 575 and 591. To more accurately predict R and transcription factor genes, we add new results from Resistify (Smith et al. 2025) and TransFacPred (Patiyal et al. 2024), more precise and comprehensive R and transcription factor genes annotation tool. Please see lines 296-301, 306-308, 576-580, 594-596, Table S27 and S28.

Reference:
Smith M, Jones JT, Hein I. Resistify: A novel NLR classifier that reveals Helitron-associated NLR expansion in Solanaceae. Bioinform Biol Insights 2025;19:11779322241308944. https://doi.org/10.1177/11779322241308944.
Patiyal S, Tiwari P, Ghai M, et al. A hybrid approach for predicting transcription factors. Front Bioinform 2024;4:1425419. https://doi.org/10.3389/fbinf.2024.1425419.

13. Lines 626-628. The percentage is low compared to what?. A likely explanation for this percentage is that the number of total genes is on the high side for a diploid species, It may be worth double checking if gene annotation was performed with a masked genome.
>>> We have rephrased these contents to make clear. Please see line 593.
We have checked our results. During the gene annotation procedure, we used the masked genome.

Miscellaneous:
1) Because there are references added and removed, the order of references in the revised manuscript are changed. Please see revised citing numbers corresponding to the reference list in the text and “References” section.
2) Because revision, we correct some errors and also remove and replace the tables/figures in the text and supplementary materials.

3) We reanalyze some of the results as suggested, and the results are revised correspondingly.

Please let us know if there are any additional concerns about our manuscript after we have offered these corrections and responses, as we are happy to address any continued issues or proposed changes to the manuscript. Thank you for your time.

Sincerely yours,

Zheng-Feng Wang
South China Botanical Garden
Chinese Academy of Sciences
Guangzhou, 510650
China

Editorial Manager®

Cookie Preference Center

We use cookies which are necessary to make our site work. We may also use additional cookies to analyze, improve and personalize our content and your digital experience. For more information, see our Cookie Policy.

You may choose not to allow some types of cookies. However, blocking some types may impact your experience of our site and the services we are able to offer. See the different category headings below to find out more or change your settings.

Allow all

Manage Consent Preferences

Strictly Necessary Cookies

Always active

These cookies are necessary for the website to function and cannot be switched off in our systems. They are usually only set in response to actions made by you which amount to a request for services, such as setting your privacy preferences, logging in or filling in forms. You can set your browser to block or alert you about these cookies, but some parts of the site will not then work. These cookies do not store any personally identifiable information.

Functional Cookies

These cookies enable the website to provide enhanced functionality and personalisation. They may be set by us or by third party providers whose services we have added to our pages. If you do not allow these cookies then some or all of these services may not function properly.

Performance Cookies

These cookies allow us to count visits and traffic sources so we can measure and improve the performance of our site. They help us to know which pages are the most and least popular and see how visitors move around the site.

Targeting Cookies

These cookies may be set through our site by our advertising partners. They may be used by those companies to build a profile of your interests and show you relevant adverts on other sites. If you do not allow these cookies, you will experience less targeted advertising.

Cookie List

Clear

Apply Cancel

Consent Leg.Interest

Confirm my choices

Powered by onetrust
