# Supplementary material for: Chromosome-scale assemblies of three Ormosia species: repetitive sequences distribution and structural rearrangement
Source: Gigascience. 2025 May 16;14:giaf047. doi: 10.1093/gigascience/giaf047 (PMC12083454; doi:10.1093/gigascience/giaf047)
Supplement: giaf047_Authors_Response_To_Reviewer_Comments_Revision_2 [file giaf047_authors_response_to_reviewer_comments_revision_2.pdf]

Author's Response To Reviewer Comments

Dear editor,
Thank you very much for your decision letter about our manuscript entitled “Chromosome-scale assemblies of three Ormosia species: Repetitive sequences distribution and structural rearrangement” (No. GIGA-D-24-00350R2), including the comments.
We are now sending our responses to comments with our revised manuscript (both tracked and clean versions). All the modified parts in the revised manuscript are marked in red.
Our specific responses are as follows:

Comments from the reviewers:
Reviewer #1:
The authors made several improvements in the manuscript to address my previous concerns. After a final round of review, I have some minor comments that I think that the authors can solve without further rounds of review. My main comment is related to the new text between lines 629-637. Lack of SNPs in repetitive regions is probably just a technical effect of the filters applied for ancestry analysis, especially deviation from HWE and LD. Repetitive regions usually have less selective pressures than regions encoding for exons, and hence they should have more diversity. In any case, a diversity analysis across regions of the genome must be done from a variation dataset with less filters than that used to make PCA or Admixture analysis. Taking into account that this analysis is not central for the description of the resource, my advice is to remove these sentences.
>>>We remove the paragraph as suggested. Because Fig. 2B is removed subsequently in the paragraph, Fig. 2C and Fig. 2D in the following paragraph are then renamed as Fig. 2B and Fig. 2C. Please see Fig. 2 and lines 629 and 634.

These are other minor comments:
Line 576-580. The text has some grammatical mistakes that make it difficult to understand the message. The second sentence should not start with the word "While" and probably "then" should be replaced with "the". Please rewrite the paragraph to improve the description of the number of NLR genes.
>>>We have rephrased the sentences as suggested. Please see lines 576-580.

Lines 594-596. Please also rephrase the description of this result. The paragraph should not finish with the word "then".
>>>We have rephrased the sentences as suggested. Please see lines 594-595.

Line 610: the word "diversity" should not be written in plural.
>>>We have corrected that. Please see line 609.

Lines 629 and 631: The word "length" should be removed.
>>>We have removed the whole paragraph as suggested in Major Comment above.

Miscellaneous:
Because there are references removed, the order of references in the revised manuscript are changed. Please see revised citing numbers corresponding to the reference list in the text (line 1005) and “References” section (lines 997-998).

Please let us know if there are any additional concerns about our manuscript after we have offered these corrections and responses, as we are happy to address any continued issues or proposed changes to the manuscript. Thank you for your time.

Sincerely yours,

Zheng-Feng Wang
South China Botanical Garden
Chinese Academy of Sciences

Guangzhou, 510650
China

Cookie Preference Center

We use cookies which are necessary to make our site work. We may also use additional cookies to analyze, improve and personalize our content and your digital experience. For more information, see our Cookie Policy.

You may choose not to allow some types of cookies. However, blocking some types may impact your experience of our site and the services we are able to offer. See the different category headings below to find out more or change your settings.

Allow all

Manage Consent Preferences

Strictly Necessary Cookies

Always active

These cookies are necessary for the website to function and cannot be switched off in our systems. They are usually only set in response to actions made by you which amount to a request for services, such as setting your privacy preferences, logging in or filling in forms. You can set your browser to block or alert you about these cookies, but some parts of the site will not then work. These cookies do not store any personally identifiable information.

Functional Cookies

These cookies enable the website to provide enhanced functionality and personalisation. They may be set by us or by third party providers whose services we have added to our pages. If you do not allow these cookies then some or all of these services may not function properly.

Performance Cookies

These cookies allow us to count visits and traffic sources so we can measure and improve the performance of our site. They help us to know which pages are the most and least popular and see how visitors move around the site.

Targeting Cookies

These cookies may be set through our site by our advertising partners. They may be used by those companies to build a profile of your interests and show you relevant adverts on other sites. If you do not allow these cookies, you will experience less targeted advertising.

Cookie List

Clear

Apply Cancel
Consent Leg.Interest

Confirm my choices

Powered by onetrust
