# Supplementary material for: Chromosome-scale assemblies of three Ormosia species: repetitive sequences distribution and structural rearrangement
Source: Gigascience. 2025 May 16;14:giaf047. doi: 10.1093/gigascience/giaf047 (PMC12083454; doi:10.1093/gigascience/giaf047)
Supplement: giaf047_Reviewer_1_Report_Original_Submission — Jorge Duitama -- 9/20/2024 [file giaf047_reviewer_1_report_original_submission.pdf]

Reviewer Report

Title: Chromosome-scale assemblies of three Ormosia species: Repetitive sequences distribution and structural rearrangement

Version: Original Submission **Date:** 9/20/2024

Reviewer name: Jorge Duitama

Reviewer Comments to Author:

The manuscript describes chromosome level genome assemblies for three species of the genus Ormosia, one of them completely sequenced for this study. They also seem to have sequenced RNA from fruit, seed, leafs and flowers and they also seem to have sequenced DNA (not clear which protocol) from 153 individuals. The genome assembly and annotation look fine for current standards. However, I have some concerns about methods as follows:1. There is very little context about important information of the sequenced species such as the ploidy and the reproductive mode (sexual reproduction, autogamous or allogamous, etc). This information is critical to know the expected assembly that should be pursued. The analysis of the genomic variation dataset should include an analysis of heterozygosity per sample to know which type of assembly should be generated. In case that a phased assembly would be appropriate, I assume that this could not be made due to the error rate of nanopore reads. This error rate should be reported and the reason for not trying a phased assembly must be clearly stated in the manuscript.2. The information related to the origin of the RNA-seq data is unclear. The text seems to indicate that RNA-seq was collected from 153 accessions, but then the SRA records suggest that only one accession was sequenced. More importantly, the experimental design did not seem to include biological replicates. This makes the results of differential expression meaningless. Taking into account that the manuscript is a data note, I recommend to completely remove the section "RNA-seq analysis of tissues". The RNA-seq data generated in this work is worth to aid the gene annotation process, but the experiment is not correctly designed to assess differential expression. As a small note, nothing is "highly significant". Significance is an assessment of probability that an event (a difference in many cases) is real. Hence, it should not be a term subject to gradation.3. Likewise, the protocol used to obtain DNA sequencing data for the diversity panel is unclear. Regarding the samples, it is not clear if the sampling is representative for the species. Regarding the data, it is not clear which protocol was used for sequencing. The text suggests that it was WGS, but the number of reported SNPs is very low for a WGS dataset of a plant species. The methods indicate that SNPs were called with a pipeline called "dDocent". Looking at the documentation, this seems to be a pipeline for RAD-sequencing data, which probably means that it is not a good option for WGS data. This could be the main reason explaining why the number of SNPs is very small for a WGS dataset. This could also explain the reported result regarding excess of heterozygosity. If the authors want to release the resequencing data an a database of genomic variation, I recommend using more standard variant calling and genotyping tools such as NGSEP or GATK and redo the downstream analysis using the possibly more than 1 million SNPs that should be obtained from these data.4. About the analysis of genetic structure, if the number of sampled populations is actually 6, it does not make sense to show the results only until k=4. The resolution of figure 2 needs to

be improved. I can not differentiate correctly the populations in the current PCA. If the most genetically distant population is NKS, it is weird that this population gets differentiated only until k=4. Again k=5 and k=6 are needed to evaluate the stability of this differentiation.Minor comments.1. The order of the figures looks disorganized relative to the order of the text. The reason for this seems to be that the map in figure 2A is cited in the context to talk about the distribution of the species. Given that this map does not actually show much of global context, I would recommend eliminating the references to figure 2A in the context and in the methods and reorganize the figures.2. The resolution of the figures 2 and 3 is very poor. This makes it difficult to follow the results included in these figures.3. Abstract: Please rephrase the second sentence of the background4. Line 40: it is not clear what "substantial" means. It is also not clear to what Ormosia is compared in the expression "...fewer transcription factor genes"5. Line 135. Replace "inputed into" with "given to"6. Lines 331 - 342. The text looks speculative and I think it is not needed for a data note.7. Lines 352-363. Again, I think that a data note does not require speculation about possible consequences of the genes that look missing in the assemblies.8. Line 392. The expression "a recent acient WGD" looks self contradictory. More importantly, the event does not seem to be genus specific, but it just seem to be the known event in fabales. The authors could simply refer to this event.9. Line 399. It is not clear what are "TD-type genes". I assume they would be the tandem duplication, but this needs to be clear in the text.10. Line 401. Likewise, it is not clear what are "PD-type genes".11. Line 541-548. Again the text looks speculative. I recommend to remove this paragraph.12. Lines 550-561. Again I think that this analysis should not be included in a data note, especially because the results do not seem to be conclusive.

Level of Interest

Please indicate how interesting you found the manuscript: Choose an item.

Quality of Written English

Please indicate the quality of language in the manuscript: Choose an item.

Declaration of Competing Interests

Please complete a declaration of competing interests, considering the following questions:

- Have you in the past five years received reimbursements, fees, funding, or salary from an organisation that may in any way gain or lose financially from the publication of this manuscript, either now or in the future?
- Do you hold any stocks or shares in an organisation that may in any way gain or lose financially from the publication of this manuscript, either now or in the future?
- Do you hold or are you currently applying for any patents relating to the content of the manuscript?
- Have you received reimbursements, fees, funding, or salary from an organization that holds or has applied for patents relating to the content of the manuscript?

- Do you have any other financial competing interests?
- Do you have any non-financial competing interests in relation to this paper?

If you can answer no to all of the above, write 'I declare that I have no competing interests' below. If your reply is yes to any, please give details below.

I declare that I have no competing interests

I agree to the open peer review policy of the journal. I understand that my name will be included on my report to the authors and, if the manuscript is accepted for publication, my named report including any attachments I upload will be posted on the website along with the authors' responses. I agree for my report to be made available under an Open Access Creative Commons CC-BY license (http://creativecommons.org/licenses/by/4.0/). I understand that any comments which I do not wish to be included in my named report can be included as confidential comments to the editors, which will not be published.

Choose an item.

To further support our reviewers, we have joined with Publons, where you can gain additional credit to further highlight your hard work (see: https://publons.com/journal/530/gigascience). On publication of this paper, your review will be automatically added to Publons, you can then choose whether or not to claim your Publons credit. I understand this statement.

Yes Choose an item.
