# Supplementary material for: Chromosome-scale assemblies of three Ormosia species: repetitive sequences distribution and structural rearrangement
Source: Gigascience. 2025 May 16;14:giaf047. doi: 10.1093/gigascience/giaf047 (PMC12083454; doi:10.1093/gigascience/giaf047)
Supplement: giaf047_Reviewer_1_Report_Revision_1 — Jorge Duitama -- 12/30/2024 [file giaf047_reviewer_1_report_revision_1.pdf]

Reviewer Report

Title: Chromosome-scale assemblies of three Ormosia species: Repetitive sequences distribution and structural rearrangement

Version: Revision 1 **Date:** 12/30/2024

Reviewer name: Jorge Duitama

Reviewer Comments to Author:

The manuscript describes the chromosome-level genome assembly and annotation of three new genomes, as well as WGS population genomics data for 6 populations of O. purpureiflora. I appreciate the effort taken by the authors to address properly most of my previous concerns. I think that the resource provided by the authors will be of great value for research in Fabales. However, I still have a some concerns related to the analysis of repetitive elements and the SNPs chosen for population genomics. These are my detailed comments:1. I think that there is an issue with the analysis of repeat density, leading to a wrong interpretation of the result. It is inconsistent to state that 75% percent of the genome is composed of repetitive elements to later say that most pericentromeric regions have low repeat density. If the latter statement would be correct, one question that would need to be addressed is which kind of DNA is present in those regions (if it is not repeats or genes). Also, to the best of my knowledge, this would be the first report in the plant kingdom (and possibly in the whole tree of life) in which gene density has positive correlation with repeat density.My guess is that the error lies in the definition of density used by the authors. Density is usually not measured in number of annotations, but in the percentage of the genome covered by such annotations. Based on the results reported in the text and shown in the supplementary figure 4 and supplementary table 5, my guess is that there is an overcounting of annotations of very small TIR and Helitron elements, which may be located close to genes, but that only contribute to about 4% of the genome. In contrast, the large LTR elements have numbers comparable to TIRs, but take 62% of the genome, probably spanning most pericentromeric regions, as suggested in the supplementary figure 4. The circos plots in figure 3 need to be corrected showing density as percentage of the genome covered. This probably will provide a more clear and correct interpretation of the distribution of repetitive elements across the genome.2. The second major concern is related to the SNP filtering procedure and its consequence on the genotype calling of the selected SNPs. The authors report a surprisingly high (> 0.85) observed average heterozygosity (supplementary table 1). This means that on average more that 85% of genotype calls for each SNP are heterozygous. Probably this is also true for each individual. This is highly unusual for diploid organisms, and it will make nearly impossible to cluster individuals in populations. This can not be explained just by outcrossing or clonal reproduction. The only species in which I have seen such genome wide heterozygosities is the decaploid sugar cane. If this would be the case, then allelic dosages need to be calculated for each datapoint to obtain a dataset useful to make genetic clustering. This result also suggest that the HWE filter was not applied or it was not effective because the maximum expected heterezygosity in diploid individuals is 0.5 and a SNP genotyped in more than 100 individuals with OH>0.85 for sure will fail HWE. The only possible biological explanation that I can figure out would be

that the population is just 153 genetic clones and then most SNPs are just the heterozygous sites of only one true genetic individual in the population.Again, I think that the problem is technical and it can be easily solved. The most likely explanation for the current outcome is that several SNPs are located in regions with transposable elements, for which no analysis pipeline for Illumina reads can provide reliable genotyping. The most important filter to apply in this case is to remove SNPs within annotated transposable elements. The resulting SNPs will not be evenly distributed in the genome, but they will be enriched in single copy (and gene rich) regions. However, genotype calls will accurate and hence the SNPs will be more reliable for sample clustering. Genome-wide low heterozygosity can easily be explained by inbreeding, but genome-wide high heterozygosity is very rare, especially in entire populations.One more time I think it is worth to calculate and present the heterozygosity per individual (this is different than the OH). Please also calculate and show the distribution of Minor allele frequency, global and per population. Both distributions can be calculated with the summary statistics of NGSEP or with VCFTools. Interpreting these distributions helps to identify technical issues and to assess the effect of population structure in allele frequencies and diversity.3. It is not clear which kind of selection was tested or to which environment refers the result regarding the SNPs predicted as "adaptive".Minor comments:1. I could not find Figure 4 in the revised version2. Line 64. The word "their" should not be capitalized3. Line 88. It should read: "… and it is primarily …"4. Lines 108-112. I do not think it is needed to list every single new genome assembly in literature and it is definitely not mandatory for this paper to include in the analysis all genomes sequenced to date. If that would be the case, then I think that, for example, several new genomes of Phaseolus and Vigna species are missing. Talking about future work in the introduction is strange. I would just remove these sentences, but if needed, they may better go to the conclusions.5. Lines 226-230. The sentence is confusing. Please take some more time and space to explain the procedure to merge the calls of transposable elements.6. Lines 316-318. I would eliminate these sentences. The goal of the paper is not to defend a particular pipeline. The cited literature does not help to make the point. Most papers cited are very old for the field, some do not use good measures of quality for benchmarking of variant callers (Yao et al., 2020 for example) and even in Schilbert et al., 2020 the reported performance for Freebayes is among the worst (See table 1). Even in this paper, the raw number of SNPs given by Freebayes means that there is a SNP every 5bp, which is unrealistically high. Leaving to the filtering step the task to achieve good quality data is not a good attibute for a variant caller.7. Lines 330-339. I am not sure what would be the effect of running the recalling step of GATK on VCFs generated using other tools, but my guess is that it may not be a good idea. Different tools have different models to estimate genotype likelihoods and even report those values using different optional fields. For this reason it is very likely that the GATK genotype recalling would only do fine for VCFs generated by GATK. This could be another technical reason for the inflated observed heterozygosity. I would calculate summary statistics before and after this step to see if they actually produce an important change in the genotype calls (and in which direction). Rather than that, it is not mandatory to include steps of the GATK pipeline if this tool could not be used for computational efficiency reasons.8. Line 495. As a side note to the concern 1, the authors should describe the procedure to calculate correlation and run a significance test for correlation.9. Line 543. Again, I would not call "recent" a WGD of more than 50 million years.10. Lines 594 and 595. Please describe which measure of correlation was used and which statistical test was applied to determine strong correlation. Moreover, the sentence says that the rearrangements colocate with tegions of high repeat density, but

the circos figures (3B) suggest that the regions with rearrangements have low repeat density.11. Line 606 and all other instances of the word "significant". Please describe the statistical test and report the p-value to determine in this case that rearrangements are a significant driving force of evolution.12. Lines 615 and 625. Please clarify the meaning of the terms "other species" and "all the other species". A high number of predicted LRR genes and/or TFs can just be caused by false positive calls.13. Lines 626-628. The percentage is low compared to what?. A likely explanation for this percentage is that the number of total genes is on the high side for a diploid species, It may be worth double checking if gene annotation was performed with a masked genome.

Level of Interest

Please indicate how interesting you found the manuscript: Choose an item.

Quality of Written English

Please indicate the quality of language in the manuscript: Choose an item.

Declaration of Competing Interests

Please complete a declaration of competing interests, considering the following questions:

- Have you in the past five years received reimbursements, fees, funding, or salary from an organisation that may in any way gain or lose financially from the publication of this manuscript, either now or in the future?
- Do you hold any stocks or shares in an organisation that may in any way gain or lose financially from the publication of this manuscript, either now or in the future?
- Do you hold or are you currently applying for any patents relating to the content of the manuscript?
- Have you received reimbursements, fees, funding, or salary from an organization that holds or has applied for patents relating to the content of the manuscript?
- Do you have any other financial competing interests?
- Do you have any non-financial competing interests in relation to this paper?

If you can answer no to all of the above, write 'I declare that I have no competing interests' below. If your reply is yes to any, please give details below.

I declare that I have no competing interests

I agree to the open peer review policy of the journal. I understand that my name will be included on my report to the authors and, if the manuscript is accepted for publication, my named report including any attachments I upload will be posted on the website along with the authors' responses. I agree for my report to be made available under an Open Access Creative Commons CC-BY license

(http://creativecommons.org/licenses/by/4.0/). I understand that any comments which I do not wish to be included in my named report can be included as confidential comments to the editors, which will not be published.

Choose an item.

To further support our reviewers, we have joined with Publons, where you can gain additional credit to further highlight your hard work (see: https://publons.com/journal/530/gigascience). On publication of this paper, your review will be automatically added to Publons, you can then choose whether or not to claim your Publons credit. I understand this statement.

Yes Choose an item.
