# Supplementary material for: Chromosome-scale assemblies of three Ormosia species: repetitive sequences distribution and structural rearrangement
Source: Gigascience. 2025 May 16;14:giaf047. doi: 10.1093/gigascience/giaf047 (PMC12083454; doi:10.1093/gigascience/giaf047)
Supplement: giaf047_Reviewer_1_Report_Revision_2 — Jorge Duitama -- 2/12/2025 [file giaf047_reviewer_1_report_revision_2.pdf]

Reviewer Report

Title: Chromosome-scale assemblies of three Ormosia species: Repetitive sequences distribution and structural rearrangement

Version: Revision 2 **Date:** 2/12/2025

Reviewer name: Jorge Duitama

Reviewer Comments to Author:

The authors made several improvements in the manuscript to address my previous concerns. After a final round of review, I have some minor comments that I think that the authors can solve without further rounds of review. My main comment is related to the new text between lines 629-637. Lack of SNPs in repetitive regions is probably just a technical effect of the filters applied for ancestry analysis, especially deviation from HWE and LD. Repetitive regions usually have less selective pressures than regions encoding for exons, and hence they should have more diversity. In any case, a diversity analysis across regions of the genome must be done from a variation dataset with less filters than that used to make PCA or Admixture analysis. Taking into account that this analysis is not central for the description of the resource, my advice is to remove these sentences.These are other minor comments:Line 576-580. The text has some grammatical mistakes that make it difficult to understand the message. The second sentence should not start with the word "While" and probably "then" should be replaced with "the". Please rewrite the paragraph to improve the description of the number of NLR genesLines 594-596. Please also rephrase the description of this result. The paragraph should not finish with the word "then".Line 610: the word "diversity" should not be written in plural.Lines 629 and 631: The word "length" should be removed.

Level of Interest

Please indicate how interesting you found the manuscript: Choose an item.

Quality of Written English

Please indicate the quality of language in the manuscript: Choose an item.

Declaration of Competing Interests

Please complete a declaration of competing interests, considering the following questions:

- Have you in the past five years received reimbursements, fees, funding, or salary from an organisation that may in any way gain or lose financially from the publication of this manuscript, either now or in the future?
- Do you hold any stocks or shares in an organisation that may in any way gain or lose financially from the publication of this manuscript, either now or in the future?

- Do you hold or are you currently applying for any patents relating to the content of the manuscript?
- Have you received reimbursements, fees, funding, or salary from an organization that holds or has applied for patents relating to the content of the manuscript?
- Do you have any other financial competing interests?
- Do you have any non-financial competing interests in relation to this paper?

If you can answer no to all of the above, write 'I declare that I have no competing interests' below. If your reply is yes to any, please give details below.

I declare that I have no competing interests

I agree to the open peer review policy of the journal. I understand that my name will be included on my report to the authors and, if the manuscript is accepted for publication, my named report including any attachments I upload will be posted on the website along with the authors' responses. I agree for my report to be made available under an Open Access Creative Commons CC-BY license (http://creativecommons.org/licenses/by/4.0/). I understand that any comments which I do not wish to be included in my named report can be included as confidential comments to the editors, which will not be published.

Choose an item.

To further support our reviewers, we have joined with Publons, where you can gain additional credit to further highlight your hard work (see: https://publons.com/journal/530/gigascience). On publication of this paper, your review will be automatically added to Publons, you can then choose whether or not to claim your Publons credit. I understand this statement.

Yes Choose an item.
