# Supplementary material for: Chromosome-scale assemblies of three Ormosia species: repetitive sequences distribution and structural rearrangement
Source: Gigascience. 2025 May 16;14:giaf047. doi: 10.1093/gigascience/giaf047 (PMC12083454; doi:10.1093/gigascience/giaf047)
Supplement: giaf047_Reviewer_2_Report_Original_Submission — Heena Ambreen -- 9/30/2024 [file giaf047_reviewer_2_report_original_submission.pdf]

Reviewer Report

Title: Chromosome-scale assemblies of three Ormosia species: Repetitive sequences distribution and structural rearrangement

Version: Original Submission **Date:** 9/30/2024

Reviewer name: Heena Ambreen

Reviewer Comments to Author:

General comment: The current state of written English in the manuscript requires attention. A comprehensive review of the English language including sentence structure and overall flow is highly recommended to enhance the quality of the manuscript.Line 40: This is not a very informative sentence to highlight the main findings. "substantial" and "fewer" are relative terms , thus substantial R genes and fewer TFs does not reveal the true extent/number of these identifed genomic regions. please reframe.Line 42: what does it mean by "missed"? missed in the analysis or absent in the species?It would be helpful to provide an account of the current status of genomic studies in the genus and may be closely related species.Line 102: Is the storage temperature for root tips indicated here, correct? Were the root tips incubated at subzero temperature?Figure 1A: Karyotype picture its loosing its relevance in the inset. Please consider reordering it.Lines 108-127: The authors have not provided details regarding the preparation of the DNA/RNA samples and sequencing libraries, which is a critical omission. Given that the study focuses on a less-studied plant species that is not well-characterized, this information is especially important for ensuring the reproducibility of the results and aiding future studies. I highly recommend including a clear description of the library preparation methods.It is unclear whether biological replicates were employed in the RNA sequencing. Please include these details.Line 129-138: Although, authors have reported the followed pipeline and softwares used for read processing and genome assembly, it is important to include details on the parameters used for each employed software. This is universally applicable to all the analysis softwares mentioned throughout the different portions of the study. These parameters shoudl be fully described.Line 166-167: It is not clear how the redundancy in repeats identified through two different programs, EDTA and RED were taken care of.Line 219-229: Identification of R genes has not been described.Line 291-293: I was expecting to see some information about how application of HiC has improved the previous reported assemblies of O. emarginata and O. semicastrataLine 294-299: BUSCO indicates completeness of genic regions of the assemblies which are often not the most difficult genome regions to map and assemble. Authors should evaluate the integrity and completeness of the assembly through other metrics. My suggestions would be performing analysis of LTR assembly index (https://academic.oup.com/nar/article/46/21/e126/5068908?login=false), mapping back the short and long-read DNA/RNA sequences onto to assembly to correlate mapping percentages with genome continuity.Line 312-316: What is the length distribution of the genes predicted in the analysis. Have an exploratory analysis done to ascertain the quality of the predicted genes? How does the authors justify only 70% of their predicted genes being annotated.Line 327: "By also removing possible LTR-mediated genes and reciprocally excluding Helitron- or TIR-mediated genes, we finally retained 7216 possible

Helitron-mediated genes and 2728 possible TIR-mediated genes." Does the author means LTRs were nested in the helitron and TIR repeats? and by removing these putative regions 7216 genes were retained from 21641? This statement is not clear.Line 361: This statement sounds very speculative. have the authors done any validation to make sure these genes are entirely missing from the assembly or have left undetected by the used pipeline?Table 2: Authors should consider elaborating the annotation details for the assemblies in the main Table which bring more visibility to the reader.The manuscript does not describe and provide information about data validation and quality control.

Level of Interest

Please indicate how interesting you found the manuscript: Choose an item.

Quality of Written English

Please indicate the quality of language in the manuscript: Choose an item.

Declaration of Competing Interests

Please complete a declaration of competing interests, considering the following questions:

- Have you in the past five years received reimbursements, fees, funding, or salary from an organisation that may in any way gain or lose financially from the publication of this manuscript, either now or in the future?
- Do you hold any stocks or shares in an organisation that may in any way gain or lose financially from the publication of this manuscript, either now or in the future?
- Do you hold or are you currently applying for any patents relating to the content of the manuscript?
- Have you received reimbursements, fees, funding, or salary from an organization that holds or has applied for patents relating to the content of the manuscript?
- Do you have any other financial competing interests?
- Do you have any non-financial competing interests in relation to this paper?

If you can answer no to all of the above, write 'I declare that I have no competing interests' below. If your reply is yes to any, please give details below.

I declare that I have no competing interests'

I agree to the open peer review policy of the journal. I understand that my name will be included on my report to the authors and, if the manuscript is accepted for publication, my named report including any attachments I upload will be posted on the website along with the authors' responses. I agree for my report to be made available under an Open Access Creative Commons CC-BY license (http://creativecommons.org/licenses/by/4.0/). I understand that any comments which I do not wish to

be included in my named report can be included as confidential comments to the editors, which will not be published.

Choose an item.

To further support our reviewers, we have joined with Publons, where you can gain additional credit to further highlight your hard work (see: https://publons.com/journal/530/gigascience). On publication of this paper, your review will be automatically added to Publons, you can then choose whether or not to claim your Publons credit. I understand this statement.

Yes Choose an item.
